# Altered pattern of proteolysis in rhegmatogenous retinal detachment by mining of N-termini datasets from vitreous humor proteome

**DOI:** 10.1038/s41598-025-19857-z

**Published:** 2025-10-14

**Authors:** Gabriele Antonio Zingale, Sara Giammaria, Irene Pandino, Luca Placentino, Guido Ripandelli, Grazia Raffaella Tundo, Giuseppe Grasso, Alessio Bocedi, Peter A. Bell, Tommaso Rossi, Diego Sbardella

**Affiliations:** 1 IRCCS Fondazione Bietti ONLUS, Rome, Italy; 2 Department of Clinical Sciences and Translational Medicine, University of Rome Tor Vergata, Rome, Italy; 3 Department of Chemical Sciences, University of Catania, Viale Andrea Doria 6, 95125 Catania, Italy; 4 Department of Chemical Sciences and Technology, University of Rome Tor Vergata, Rome, Italy; 5 Center for Blood Research, University of British Columbia, Vancouver, Canada; 6 Department of Oral Biological and Medical Sciences, University of British Columbia, Vancouver, Canada

**Keywords:** Vitreous humor, Proteomics, N-termini, Proteolysis, Angiogenesis, Biochemistry, Proteases, Proteolysis, Proteomics, Predictive markers

## Abstract

Rhegmatogenous Retinal Detachment (RRD) is a severe ocular condition characterized by the detachment of the neurosensory retina from the retinal pigment epithelium and caused by retinal tears. Pars Plana Vitrectomy (PPV) is the standard surgical procedure for RRD, and is intended to remove the vitreous gel, which shapes the eye, and provides mechanical and nutritional support to the retina. The study of the vitreous proteome isolated from RRD patients may help decipher the pathobiology of the disease and that of its complications, such as proliferative vitreo-retinopathy (PVR), which predispose to recurrent retinal detachment (observed in 20% of cases), a sight threatening condition. Herein, we set up a pilot shot-gun proteomics study (Data are available via ProteomeXchange with identifier PXD057155) to investigate the perturbations of vitreous proteome comparing RRD patients (*n* = 8) to patients affected by idiopathic Epiretinal Membranes (ERM), used as controls (*n* = 8). Spectra were first searched and analyzed to identify proteome perturbations. Thereafter, starting from the hypothesis that RRD could be sustained by altered proteolytic processing of structural and non-structural elements of vitreous gel, N- and C-termini were mined to uncover endogenous proteolytic events. Our search revealed a comprehensive array of proteolytic events and sites across numerous proteins. While some of these were previously documented in different biological samples (like plasma, cell models, etc.), our findings point to new, potentially specific cleavage sites on macromolecular components unique to the vitreous fluid and retinal layers (e.g., interphotoreceptor matrix proteoglycan 1 and 2). Comparison between the N- and C-termini landscapes and the perturbations of global proteome highlighted robust alterations of the repertoire of cleaved proteins between RRD patients and reference control subjects. Additional immunoblotting studies on a selection of proteins envisage that RRD is characterized by unbalanced proteolysis of structural and non-structural components involved in the regulation of immune processes, proteolytic control and, particularly, angiogenesis.

**Supplementary Information:**

The online version contains supplementary material available at 10.1038/s41598-025-19857-z.

## Introduction

Rhegmatogenous Retinal Detachment (RRD) is a severe condition characterized by the detachment of the neurosensory retina from the Retinal Pigment Epithelium (RPE) due to the presence of retinal tears^1–4^. The incidence of RRD is 7 to 12/10.000 cases per year^1^ with a guarded prognosis and a heavy burden for the patients’ quality of life and health care expenditures^5^. Recurrent retinal detachment occurs in up to 20% of cases, leading to severe visual loss and often requiring multiple surgery^1^.

Proliferative Vitreoretinopathy (PVR) is the primary cause of recurrent retinal detachment and subsequent severe visual loss. It is characterized by the growth and contraction of cellular aggregates on the retinal surface and within the vitreous chamber^6–8^.

A hallmark of PVR is the Epithelial-to-Mesenchymal Transition (EMT), where retinal pigment epithelial (RPE) cells acquire mesenchymal characteristics. This transformation leads to altered blood hemodynamics, changes in vessel microarchitecture, fibrosis, and ultimately, retinal contraction^2,6^. While the molecular footprint and cascade of events driving EMT and the associated pathological retinal remodeling in PVR have been extensively studied, they are still not fully understood.

In this context, the ultrastructural and microarchitectural composition of extracellular matrices (ECM) plays a central role in cellular and tissue homeostasis. Beyond providing mechanical, structural, and nutritional support, the ECM also serves as a reservoir of bioactive peptides that potently regulate cell behavior and function^9–11^.

Accordingly, previous studies have demonstrated significant changes in RPE cell metabolism, mRNA transcription, and translation in relation to the stiffness of Bruch’s membrane^12^. Bruch’s membrane, the specialized ECM of the RPE, itself exhibits altered properties in various retinal pathological processes.

Consistent with this scenario, dysregulation of photoreceptor metabolism, cell adhesiveness and polarization, extracellular matrix turnover and remodeling, immune system crosstalk, redox imbalance, angiogenesis, and inflammation have also been documented during PVR^2,6,13–15^.

Pars Plana Vitrectomy (PPV) is the standard surgical procedure for complex RRDs and allows the collection of vitreous (VH) and aqueous (AH) humor samples^16^. Research activities on these fluids are intrinsically complex since protein concentration is typically very low and identification of relevant proteins is hidden by abundant contaminating proteins (e.g., albumin, IgG etc.), often requiring sample pooling procedures and off-line separation or depletion of these proteins.

Previous relevant proteomics studies applied to the human vitreous and specifically to RRD patients suggested novel molecular perspectives on RRD and PVR pathobiology^17–21^. However, to date, post-translational modification (PTM) profiling of VH samples has not yet (to the best of our knowledge) been performed. PTMs such as phosphorylation, glycosylation, and proteolytic processing are able to profoundly alter the structure and function of a protein^22,23^. In this regard, we reasoned that retinal detachment could be linked to the dysregulation of physiological PTMs and, in particular, endogenous proteolytic events carried out by the different classes of enzymes populating the VH.

The present study reports the results of a pilot shotgun Label Free Quantification (LFQ) proteomic characterization of vitreous samples collected from a relatively small cohort of primary RRD patients (*n* = 8), compared to Controls (*n* = 8) who underwent vitreoretinal surgery for formation of epiretinal membranes (ERM). Without immunodepleting for most abundant proteins and working on a single-subject scale, using a moderated Bayesian t-test we identified a total 93 differentially expressed proteins (DEPs) between RRD and Control VH, uncovering precise alterations of key pathways for cell metabolism and tissue homeostasis. Thereafter, using proteomics analysis tools,^22,24^ we explored the repertoire of endogenous N-termini (and C-termini). This approach identified several known and unknown sites of proteolysis in structural and non-structural VH components and significant alterations on proteins serving roles for immune system regulation, proteolytic balance and, particularly, angiogenesis. This scenario introduces the perspective that the pathogenesis of RRD progresses through unbalanced extracellular proteolysis.

## Methods

### Ethics approval – Study design

The study (P.R.O. project) was submitted by the proponent institution, IRCCS Fondazione Bietti, to the local ethic committee, Territorial Ethics Committee Lazio Area 5 (P.R.O. Project, first approved - ifo_058.IFO_AOO.REGISTRO UFFICIALE.0014544, approved on 11-15-2023; updated version approved - ifo_058.IFO_AOO.REGISTRO UFFICIALE.0000158, approved on 01-02-2024). The main goal of the study is to collect vitreous and aqueous humour to investigate pathogenic determinants and potential biomarkers of most prevalent retinal disorders, including RRD, by proteomics approaches.

Samples collected are stored in an internal biobank of the institute.

In accordance to the tenets of the Declaration of Helsinki, enrolled patients were asked to read and sign an informed consent form. The authors affirm that human research participants (or their legal guardians) provided informed consent, for publication of the identifying demographic and clinical details in Table 1. Inclusion criteria for cases: age > 18 years, diagnosis of primary RRD requiring PPV repair in phakic or pseudophakic patients.

**Table 1:** Demographic and clinical parameters of subjects’ enrolled in the study.

ID	Gender	Age*	Comorbidity	Systemic Therapy	Fundus Oculi	Eye	Visual Acuity**	Pseudophakia
RRD #1	M	68	Systemic Hypertension, Gilbert syndrome	β-blocker, Ca^2+^ channel blocker, ARB	Supero-Temporal RRD, Vitreal Proliferation Signs, Macula Off, normal optic disc	R	Motu Manu	No
RRD #2	M	74	/	/	Infero-Temporal RRD, Vitreal Proliferation Signs, Macula Off, normal optic disc	R	Motu Manu	Yes
RRD #3	M	73	Systemic Hypertension, myocardial infarction	Sartan, β-blocker, Xa Factor inhibitor, Antiarrhythmic	Supero-Temporal RRD, Vitreal Proliferation Signs, Macula Off, normal optic disc	R	Light Perception	No
RRD #4	M	67	Systemic Hypertension	β-blocker	Supero-Temporal RRD, Vitreal Proliferation Signs, Macula Off, normal optic disc	L	Light Perception	No
RRD #5	F	73	Systemic Hypertension, Osteoporosis	Hydrochlorothiazide + sartan, Vitamin D	Subtotal RRD, Macula Off, no signs of vitreal proliferation, normal optic disc	R	1/20	Yes
RRD #6	F	62	Systemic Hypertension, myocardial infarction, deep vein thrombosis	β-blocker, Xa Factor inhibitor	Infero-Temporal RRD, Macula On, no signs of vitreal proliferation, normal optic disc	R	3/10	No
RRD #7	F	67	/	/	Inferior RRD, Macula Off, no signs of vitreal proliferation, normal optic disc	L	Motu Manu	Yes
RRD #8	M	65	Systemic Hypertension	β-blocker, ACE Inhibitor	Inferior RRD, Vitreal Proliferation Signs, Macula Off, normal optic disc	R	Motu Manu	Yes
CONTROL #1	M	80	Systemic Hypertension, Dysthyroidism, Hypercholesterolemia, Benign prostatic hypertrophy	β-blocker, levothyroxine, 5 alpha reductase inhibitor, Statin	Epiretinal Membrane, normal optic disc, no peripheral vitreoretinal lesions	R	5/10	Yes
CONTROL #2	M	81	/	/	Epiretinal Membrane, normal optic disc, no peripheral vitreoretinal lesions	L	3/10	No
CONTROL #3	F	82	/	/	Epiretinal Membrane, normal optic disc, no peripheral vitreoretinal lesions	L	4/10	No
CONTROL #4	F	78	Systemic Hypertension, Atrial fibrillation	Proton pump inhibitor, β-blocker, ACE inhibitor	Dislocated IOL in vitreous chamber. Normal macula and optic disc, no peripheral vitreoretinal lesions	L	1/20	No
CONTROL #5	M	72	/	/	Epiretinal Membrane, normal optic disc, no peripheral vitreoretinal lesions	L	1/10	No
CONTROL #6	F	72	Systemic Hypertension, Hypercholesterolemia, Osteoporosis	Hydrochlorothiazide + Sartan, Statin, Vitamin D	Macular Hole, Epiretinal Membrane, normal optic disc, no peripheral vitreoretinal lesions	R	1/10	No
CONTROL #7	M	68	/	/	Macular Hole, Epiretinal Membrane, normal optic disc, no peripheral vitreoretinal lesions	L	1/10	No
CONTROL #8	M	68	/	/	Epiretinal Membrane, normal optic disc, no peripheral vitreoretinal lesions	L	1/10	No

*Years; **Decimals; RRD = Rhegmatogenous Retinal Detachment; IOL = Intraocular lens.

Inclusion criteria for controls: age > 18 years, patients undergoing PPV with a diagnosis of idiopathic ERM, both phakic and pseudophakic.

Exclusion criteria (apply to both experimental groups): any ocular co-morbidity including age related macular degeneration, high myopia exceeding 6 diopters, eyes receiving intraocular therapies for any reason, including vascular occlusion, glaucoma, concomitant neurodegenerative, inflammatory or infective eye diseases and systemic conditions including diabetes, collagenopathies (e.g. Ehlers-Danlos Syndrome), cancer (local and systemic), vitreous hemorrhages, alterations of the electrophoretic profile of the gamma-globulin band.

Vitreous samples were obtained from patients undergoing PPV for either RRD or ERM peeling (Control). Initially, *n* = 9 RRD cases and *n* = 9 Controls were enrolled in the study and analysed by mass spectrometry (MS). One subject per group was then filtered out from analysis for technical issues during sample preparation. Raw mass spectrometry data of these samples were uploaded (corresponding to original samples #6 and #16) in the PRIDE repository (see “Data availability statement”) together with all samples effectively analysed and part of the study.

Selection of samples was based on the best matching of demographic and clinical characteristics of the subjects. These data are introduced in the results section and further reported in Table 1.

Approximately 1 mL of vitreous fluid was collected using a sterile 5 mL syringe and poured into a sterile 1.7 mL Eppendorf test tube. Vitreous samples were then immediately cleared by centrifugation to remove eventual debris, cells or aggregates. Protein concentration of individual vitreous samples was then quantified by bicinchoninic acid assay (BCA) following manufacturer’s instruction either for measurements and for preparation of the calibration curve (Fisher Scientific, Waltham, MA, USA). Thereafter, samples were subdivided into different aliquots and stored at -80 °C until use. No freeze/thaw cycles were applied for proteomics or Western blotting (Wb) studies, since individual aliquots were used for the two approaches.

All samples discussed in the study were processed and analysed in parallel. Anonymity of subjects was guaranteed throughout the entire study.

### Mass spectrometry settings and search parameters

In the case of proteomic analysis, samples (100 µg of VH proteins) were dehydrated in a SpeedVacuum system, reconstituted in denaturing buffer (6 M guanidine-HCl, 50 mM Hepes, pH 7.8) supplemented with inhibitors of peptidases and proteases (Merck, Darmstadt, Germany).

Proteins were then reduced [5 mM dithiothreitol, 45 min, room temperature (r.t.)], alkylated (10 mM iodoacetamide, 30 min, r.t) and digested with trypsin (1:50 enzyme: protein ratio, overnight, 37 °C) (Fisher Scientific, Waltham, MA, USA).

Trypsin digestion was quenched with 0.4% trifluoroacetic acid (TFA) and peptides cleaned using Stage-Tips (C18 resin) (Fisher Scientific, Waltham, MA, USA).

Thereafter, eluted peptides were dried by SpeedVacuum system and resuspended in 2% Acetonitrile, 0.05% Trifluoracetic Acid for mass spectrometry injection.

Proteomic analysis was performed injecting (twice) 1 µg peptides for each experimental conditions into an Orbitrap Exploris 240 mass spectrometer coupled to an Ultimate 3000 nano-ultra high performance liquid chromatography (nano-UHPLC) system. Solvent A: 100% H_2_O, 0.1% Formic Acid; Solvent B: 80% Acetonitrile, 0.1% Formic Acid. UHPLC Gradient (minutes - %B): 0–6.7; 2–6.7; 62–34.4; 67–55.5; 72–100; 80–100; 82 − 6.7; 88 − 6.7. Column oven temperature: 45 °C. Run time: 88 min. Loading Pump flowrate: 30 µL/min. NC Pump flowrate: 250 nL/min. Data acquisition was conducted in Data Dependent Acquisition (DDA) mode.

Orbitrap Resolution: 120,000; Scan Range (m/z): 375–1650; RF Lens (%): 80; Normalized AGC Target (%): 300. ddMS² was triggered using the following filters: Isolation Window (m/z): 2; Normalized HCD Collision Energy (%): 30; Orbitrap Resolution: 15,000; Normalized AGC Target (%): 50. Protein and peptides were searched using Proteome Discoverer (PD) software (v. 2.5, Thermo Fisher Scientific) against a UniProt human protein FASTA database including protein isoforms. Sequest implemented with the Inferys rescoring algorithm was used and a concatenated target-decoy strategy applied for determination of the proteins False Discovery Rate (strict FDR ≤ 0.01 and relaxed FDR ≤ 0.05).

Trypsin (full) was set as enzyme, 10 ppm precursor mass tolerance and 0.02 Da fragment mass tolerance. Carbamidomethylation of cysteines (+ 57.021) was set as static modification, whereas oxidation on methionine (+ 15.995) as dynamic modification per peptides according to the requirements of Inferys algorithm.

The search of N-termini and C-termini was instead run using FragPipe/MsFragger (v. 22.0, https://github.com/Nesvilab/FragPipe/releases), as reported elsewhere^23^.

### Western blotting studies

A selection of proteins identified by the proteomic approach was further analyzed by denaturing and reducing Western blotting (Wb) using an aliquot of VH sample different from the one used for MS analysis. In fact, guanidine-HCl denaturation step, which is required for N-termini MS studies, is incompatible with SDS-PAGE electrophoresis. For Wb studies, the vitreous fluid of the same subjects enrolled in the MS analysis (*n* = 8 per experimental group) was used without introducing any dehydration or chemical manipulation.

In all cases, 5 µg of vitreous proteins per subject were heat-denatured and reduced in Laemmli buffer 1x supplemented with 1 mM dithiothreitol (DTT). Thereafter, 4–20% acrylamide pre-cast gels (Bio-Rad, Hercules, CA, USA) were used to separate proteins by SDS-PAGE. After separation, proteins were transferred to a HyBond-ECL nitrocellulose filters (Bio-Rad, Hercules, CA, USA), stained with Ponceau S, which is now often preferred for normalization procedures, and probed with the antibodies indicated^25^.

Primary antibodies raised against anti-Dickkopf homolog 3 protein (DKK3, cat. #10365-1-AP, rabbit ), anti-osteopontin (OPN, cat. #22952-1-AP, rabbit), anti-vitronectin (VNT, cat. 15833-1-AP, rabbit) were purchased from ProteinTech (Rosemont, IL, USA). The anti-interphotoreceptor matrix proteoglycan 1 (IMPG1 – SPACR, sc-377366, cat. # lot C1220, mouse) antibody was purchased from Santa Cruz Technology (Dallas, Texas, USA). The anti-interphotoreceptor matrix proteoglycan 2 (IMPG2, cat. #PACO61546, rabbit) was purchased from AssayGenie (Dublin, Ireland).

All primary antibodies were diluted 1:3000 in 0.1% Tween-PBS 0.1% fat-free milk and with a horseradish peroxidase-conjugated anti-rabbit or anti-mouse IgG antibody (Cell Signaling Technologies, cat. #7074P2 and cat. #7076P2, respectively), diluted 1:10.000 in 0.1% Tween-PBS 0.1% fat-free milk.

Proteins were developed by ECL chemiluminescence and recorded in a iBright 1500 (ThermoFisher scientific). Band intensity, including Ponceau S pattern, was done using the iBright analysis software incorporated in the instrumentation. Uncropped Wb data are provided in Supplementary Fig. 4.

### Statistical analysis

Identifications and quantification of proteins and peptides was done using in-house built R Studio scripts (v. 4.3.1, https://posit.co/download/rstudio-desktop/). Raw data were first analysed using a pre-built method configured in PD software based on pairwise median ratio comparison between all combinations of subjects of the two groups, followed by a background based Student’s t test and Benjamini-Hochberg (BH) correction.

To address the limited sample size of the study, a class-specific quantile normalization on non-normalized protein intensities computed by PD processing, was applied. Imputation of missing values was done using the Classification and Regression Tree Method (CART) implemented in mice package in R Studio. A moderated Bayesian t-test (LIMMA) was then used to identify differentially expressed proteins (DEPs) between the two experimental groups. The analysis was performed in R using the *limma* and *Q* packages. Significance was set at p.mod value ≤ 0.05 with FDR control (q.mod) ≤ 0.05.

Since this approach and the values obtained (p.mod and q.mod by Storey’s test for FDR control) show optimized robustness for limited sample size studies, the data discussed in this paper are based on this approach. Nevertheless, the PD approach provided a very similar outcome.

Additional details regarding the differential expression of proteins and peptides between RRD and control groups are described throughout the results section.

Gene Ontology (GO) analysis was performed using the R package *ClusterProfiler* and setting a FDR ≤ 0.05 threshold after BH correction.

Histograms and statistical tests of Western blotting data were generated using GraphPad Prism software (v8.0). A non-parametric Mann-Whitney test was used to compute the p-value, with the significance threshold set at *p* ≤ 0.05.

## Results

### Subjects enrollment and study design

Vitreous samples were obtained from patients undergoing PPV for either RRD (*n* = 8, mean age 69 ± 4 years) or idiopathic ERM (*n* = 8, mean age 75 ± 6 years), enrolled as controls (Control). Additional demographic, epidemiological and clinical data of enrolled patients are summarized in Table 1. Systemic hypertension was the prevalent co-morbidity across enrolled subjects. A heterogeneous localization (supero-temporal and inferior) of the retinal detachment was documented in the RRD group.

**Fig. 1:**
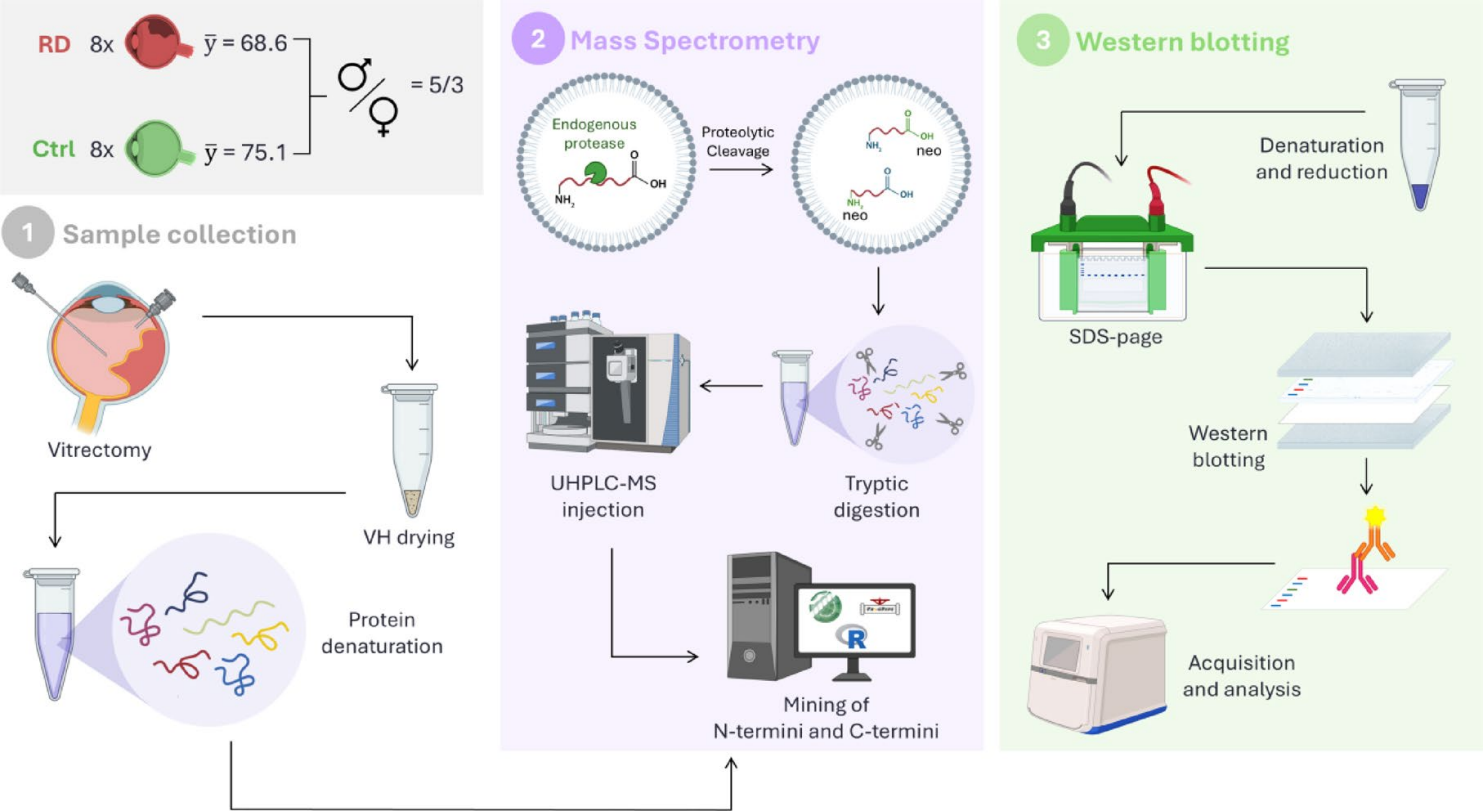
Schematic representation of the experimental design and study workflow. Created with icons from BioRender.com. For step “3” (Western blotting), a different aliquot of the original VH sample was used without drying the sample. The graphical abstract was created with icons from BioRender.com.

An equal quantity (µg) of VH protein for each enrolled sample was digested with trypsin and subjected to proteomics analysis. A workflow of the study is summarized in Fig. 1.

### Dysregulation of metabolic pathways and structural components of the ECM in VH from RRD patients

MS spectra were searched against a human FASTA database and results were then filtered for Master proteins, as they are defined in PD glossary, setting as thresholds proteins identified with ≥ 1 unique peptides and FDR ≤ 0.05. Technical contaminants (e.g., trypsin, keratin, etc.) were filtered out.

**Fig. 2:**
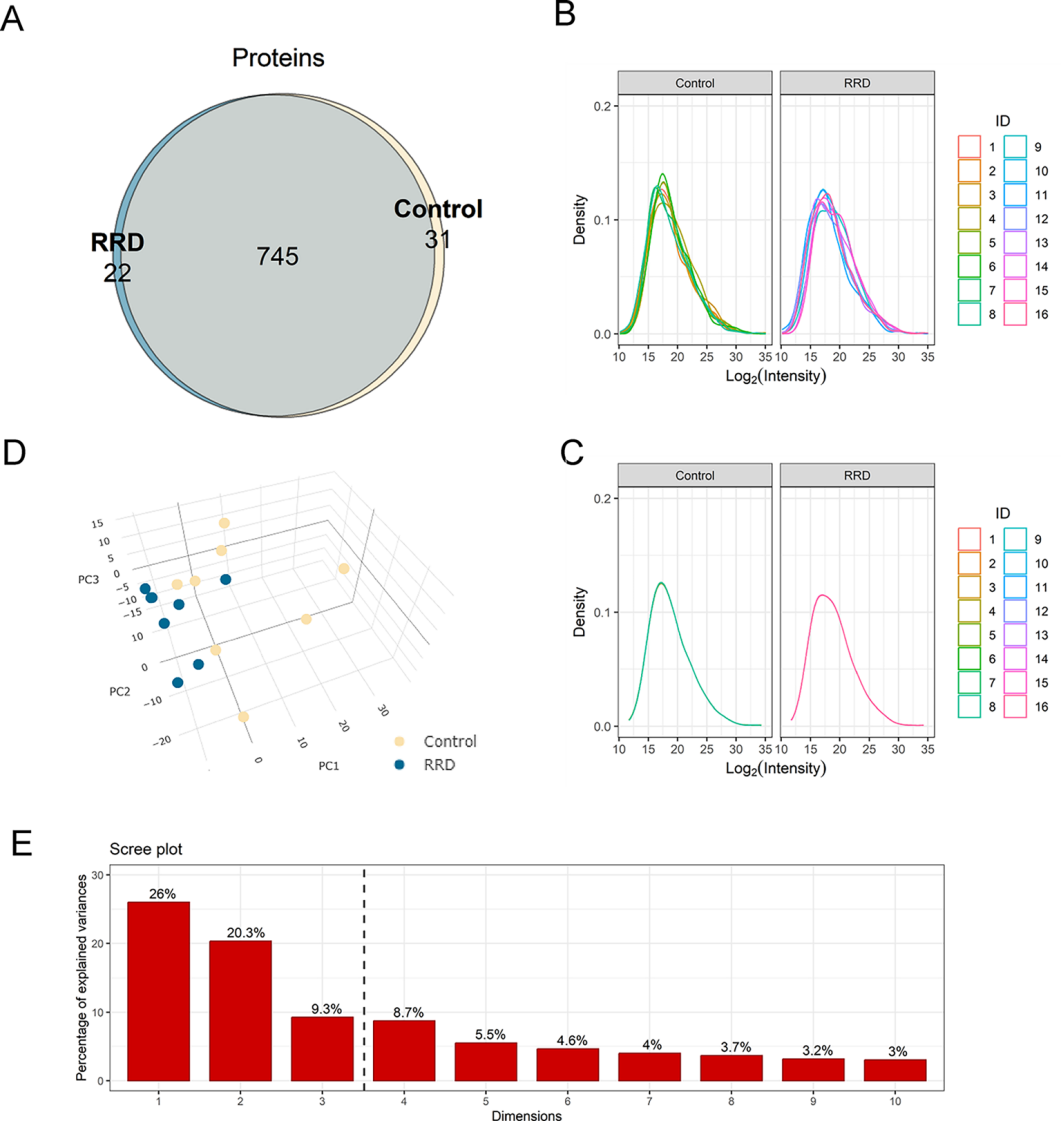
(**A**) Venn diagram showing the number of proteins identified, either common to RRD and Control, or specific to one of the two experimental groups; (**B**) Density plot of log_2_ transformed protein intensities without applying quantile normalization; (**C**) Density plot of log_2_ transformed protein intensities after application of class-specific quantile normalization; (**D**) 3D PCA analysis of Control (yellow) and RRD (blue) data. The two groups showed a good 3D separation across the three main components (i.e., PC1, PC2 and PC3); (E) Scree plot of the PCA analysis. Histograms report the contribution of each PC dimension to explaining the variance between the two groups. The dashed line is set between the PC3 and PC4 dimensions. The 3 main PCs explains > 50% variance.

In total, 798 proteins were identified (624 with FDR ≤ 0.01) and they showed

a robust overlap between RRD and Control groups: 745 proteins were common to the two experimental groups, 22 proteins were documented as exclusive of RRD and 31 of Controls. Discussion of exclusive proteins is limited to those identified and quantified in ≥ 50% subjects/group (prior to missing value imputation) to strengthen the consistency of their observation (Fig. 2A). Supplementary Tables 1 and 2 report the list of proteins exclusive for RRD and Control groups, respectively.

The distribution of protein intensities was verified upon log_2_ transformation (Fig. 2B). Density-plots of non-normalized intensities, though highlighting moderate intragroup variability, suggested a comparable intra-group and inter-group distribution for both RRD and Control datasets. Therefore a class-specific quantile normalization strategy was deemed applicable^26^. The experimental variability improved, as shown in the post-quantile normalization density plot (Fig. 2C). Thereafter, missing values were imputed using Classification and regression trees (CART) approach^27^.

Data were then analyzed by Principal Component Analysis (PCA) to further check for global differences between RRD and Control experimental groups; separation across the three principal components (PC1, PC2, PC3) suggested that RRD and Control proteins had indeed different features (Fig. 2D). A scree plot highlighted that the 3 PCs explained more than 50% of features between the two groups (Fig. 2E), confirming this hypothesis.

**Fig. 3:**
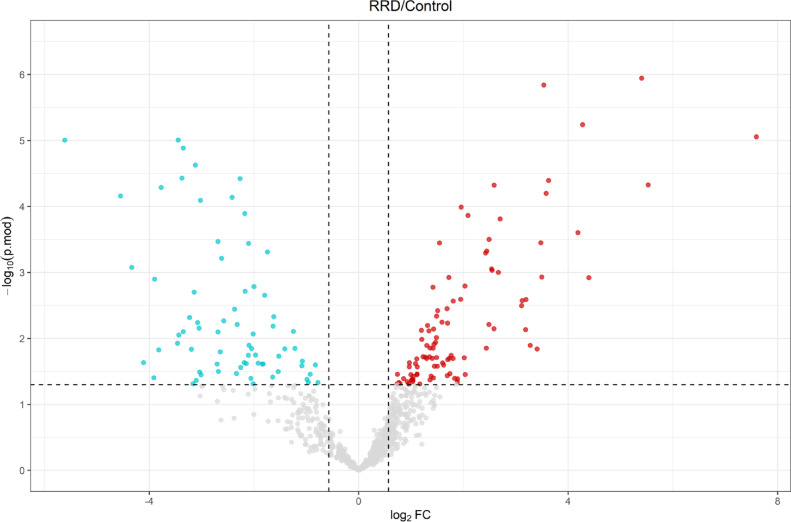
Volcano plot showing DEPs. X-axis reports the fold change expressed as log_2_ fold-change (Log_2_FC); Y-axis reported the -log_10_ p.mod-value computed by Limma. Statistical significance was set for p.mod ≤ 0.05. Dashed lines highlighted the cut-off set for log_2_FC and log_10_ of p.mod significance, namely: ±0.57 and 1.3, respectively. Proteins upregulated and downregulated in RRD vs. Control VH are represented in red and turquoise, respectively. A.

Differentially Expressed Proteins (DEPs) between RRD and Control subjects were then analyzed using a moderate Bayesian t test LIMMA statistical approach (log_2_FC ≤ 0.57 and log_2_FC≤-0.57). To address the impact of the relatively small sample size, we then analyzed data using a robust statistical method that employs the evaluation of the moderated p.values (p.mod), which are calculated borrowing variance estimates from all proteins. FDR control of DEPs was then run using the Storey’s q.value (q.mod value) (Fig. 3).

**Fig. 4:**
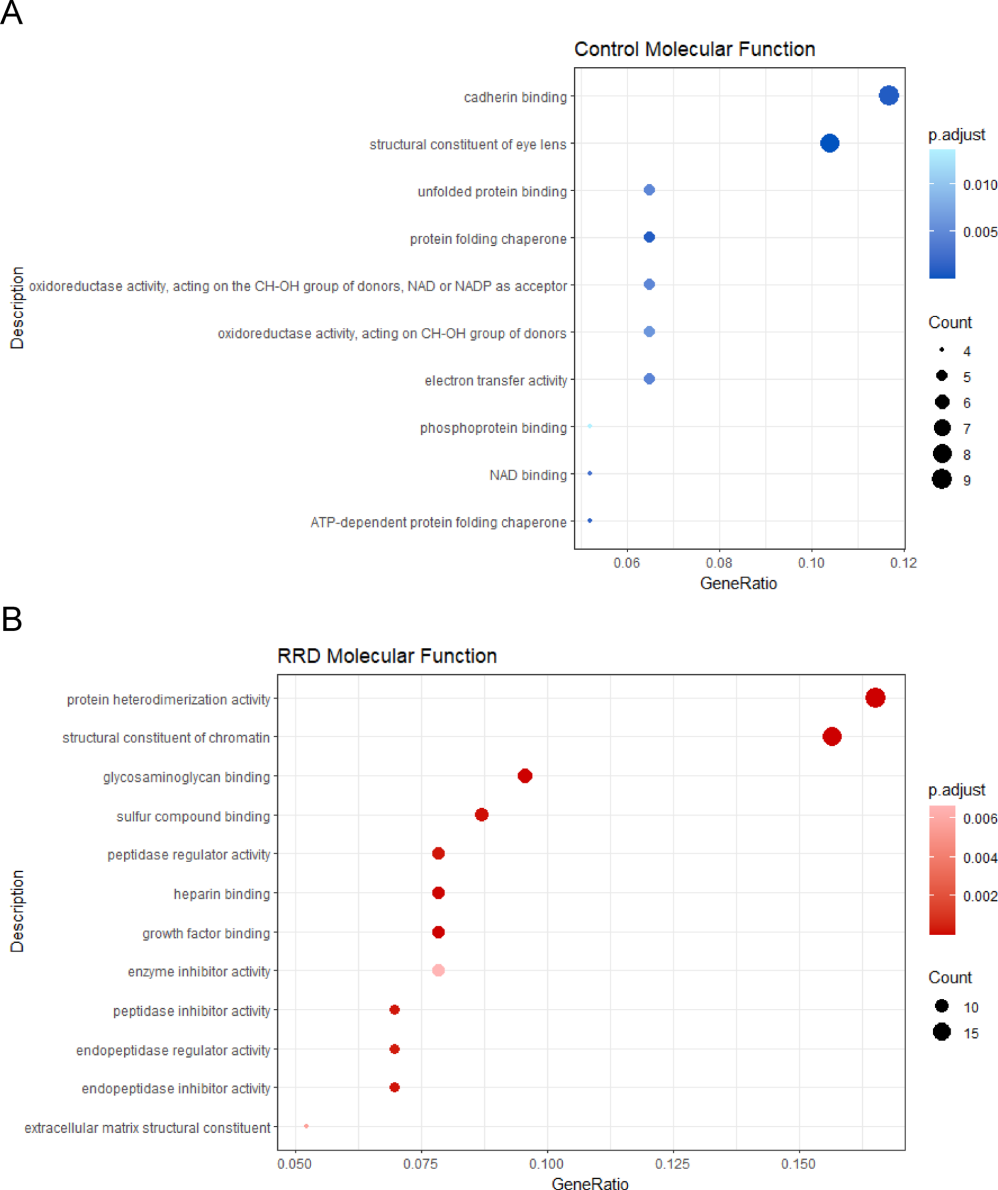
MF chart showing the terms enriched by submitting proteins upregulated or exclusive of Control (**A**) and RRD (B) VH to GO. GeneRatio, which corresponds to the proportion of genes the dataset annotated with GOTERM, was calculated and data filtered for p_adj_≤0.05 after BH correction for multiple comparisons (GO with a count ≥ 4 and ≥ 6 are shown for (**A**) and (**B**), respectively.

In total, 52 proteins were found upregulated and 41 downregulated in the RRD group, compared to Controls (RRD/Control ratio); the entire list of upregulated and downregulated proteins in RRD and Control VH is shown in Tables 2 and 3, respectively. To better cluster and rationalize data, DEPs discussed above and group-exclusive proteins identified and quantified were submitted to gene ontology (GO) to infer statistically enriched (*p* ≤ 0.05 after Benjamini-Hochberg correction for multiple testing) molecular function (MF) (Fig. 4A, B), cellular components (CC) (Suppl. Figure 1), and biological processes (BP) charts (Suppl. Figure 2).

**Table 2:** Proteins upregulated in RRD VH (vs. Control). The table shows the accession number (UniProt), the log2FC calculated for the RRD/control ratio, the q.mod value computed by LIMMA analysis, the protein description, the number of peptides identified and the FDR of protein identification from MS spectra.

Accession	logFC	q.mod	Description	#Peptides	FDR
P01011	1.316	0.030	Alpha-1-antichymotrypsin	31	≤ 0.01
A0A1B0GWE8	1.439	0.046	Cathepsin D	29	≤ 0.01
P10523	4.277	0.000	S-arrestin	27	≤ 0.01
P36222	2.538	0.008	Chitinase-3-like protein 1	26	≤ 0.01
B4DPQ0	1.341	0.033	Complement subcomponent C1r	25	≤ 0.01
P02788	2.487	0.029	Lactotransferrin	22	≤ 0.01
Q17R60	3.535	0.000	Interphotoreceptor matrix proteoglycan 1	22	≤ 0.01
A0A7I2V2D2	1.431	0.032	Plasma protease C1 inhibitor	20	≤ 0.01
P62873	3.122	0.016	Guanine nucleotide-binding protein G(I)/G(S)/G(T) subunit beta-1	19	≤ 0.01
P02042	3.110	0.019	Hemoglobin subunit delta	15	≤ 0.01
P08571	1.418	0.012	Monocyte differentiation antigen CD14	15	≤ 0.01
P07858	1.488	0.025	Cathepsin B	12	≤ 0.01
P69905	3.407	0.048	Hemoglobin subunit alpha	12	≤ 0.01
Q9BZV3	3.479	0.004	Interphotoreceptor matrix proteoglycan 2	12	≤ 0.01
P01033	1.686	0.020	Metalloproteinase inhibitor 1	11	≤ 0.01
Q6EMK4	2.422	0.005	Vasorin	11	≤ 0.01
P07602	1.946	0.016	Prosaposin	10	≤ 0.01
Q92743	1.469	0.044	Serine protease HTRA1	10	≤ 0.01
P08779	1.364	0.048	Keratin, type I cytoskeletal 16	9	≤ 0.01
P61769	1.301	0.046	Beta-2-microglobulin	9	≤ 0.01
P63211	5.403	0.000	Guanine nucleotide-binding protein G(T) subunit gamma-T1	7	≤ 0.01
Q96JP9	2.445	0.005	Cadherin-related family member 1	6	≤ 0.01
D6RHI9	1.484	0.038	Ribonuclease T2 (Fragment)	5	≤ 0.01
P07205	2.584	0.032	Phosphoglycerate kinase 2	5	≤ 0.01
P55058	2.086	0.002	Phospholipid transfer protein	5	≤ 0.01
Q8N114	3.579	0.001	Protein shisa-5	4	≤ 0.01
Q9HCQ7	2.668	0.008	Pro-FMRFamide-related neuropeptide VF	4	≤ 0.01
B3KRD8	3.275	0.046	Section 14-like 2 (S. cerevisiae), isoform CRA_c	3	≤ 0.01
E9PEK4	2.488	0.004	Receptor protein-tyrosine kinase	3	≤ 0.01
O95897	2.549	0.008	Noelin-2	3	≤ 0.01
P29279	1.957	0.002	CCN family member 2	3	≤ 0.01
Q14974	3.193	0.016	Importin subunit beta-1	3	≤ 0.01
Q8NBJ4	1.542	0.004	Golgi membrane protein 1	3	≤ 0.01
A0A7P0T9A7	3.187	0.032	Adenylosuccinate lyase	2	≤ 0.01
B0QYH5	3.626	0.001	Seizure 6-like protein	2	≤ 0.01
H0YBL1	3.495	0.009	Inositol-1-monophosphatase (Fragment)	2	≤ 0.01
P17936	1.198	0.032	Insulin-like growth factor-binding protein 3	2	≤ 0.01
P30740	4.397	0.009	Leukocyte elastase inhibitor	2	≤ 0.01
Q9NP84	2.029	0.012	Tumor necrosis factor receptor superfamily member 12 A	2	≤ 0.01
Q9UHI8	1.696	0.029	A disintegrin and metalloproteinase with thrombospondin motifs 1	2	≤ 0.01
A0A2R8YFQ7	1.507	0.021	Lambda-crystallin homolog	1	≤ 0.05
A0A8Q3WKS4	1.206	0.040	Lactadherin	1	≤ 0.01
E9PK73	5.529	0.001	Succinate dehydrogenase [ubiquinone] cytochrome b small subunit	1	≤ 0.05

**Table 3:** Proteins downregulated in RRD VH (vs. Control). The table shows the accession number (UniProt), the log2FC calculated for the RRD/control ratio, the q.mod value computed by LIMMA analysis, the protein description, the number of peptides identified and the FDR of protein identification from MS spectra.

Accession	logFC	q.mod	Description	# Peptides	FDR
P00352	-3.349	0.033	Aldehyde dehydrogenase 1A1	42	≤ 0.01
P53674	-3.459	0.045	Beta-crystallin B1	42	≤ 0.01
P48637	-3.230	0.026	Glutathione synthetase	31	≤ 0.01
P05813	-3.819	0.048	Beta-crystallin A3	27	≤ 0.01
P29401	-3.434	0.035	Transketolase	25	≤ 0.01
Q00796	-3.047	0.032	Sorbitol dehydrogenase	18	≤ 0.01
P53672	-4.549	0.001	Beta-crystallin A2	17	≤ 0.01
P04792	-3.898	0.010	Heat shock protein beta-1	11	≤ 0.01
Q5TDP6	-3.195	0.048	Lengsin	10	≤ 0.01
Q93088	-3.076	0.029	Betaine–homocysteine S-methyltransferase 1	8	≤ 0.01
Q96GW7	-2.168	0.013	Brevican core protein	8	≤ 0.01
P15121	-3.143	0.013	Aldo-keto reductase family 1 member B1	7	≤ 0.01
P30041	-3.447	0.000	Peroxiredoxin-6	7	≤ 0.01
P00390	-2.319	0.029	Glutathione reductase, mitochondrial	6	≤ 0.01
P07900	-2.578	0.028	Heat shock protein HSP 90-alpha	6	≤ 0.01
P0DOX3	-3.374	0.001	Immunoglobulin delta heavy chain	5	≤ 0.01
P22061	-2.093	0.046	Protein-L-isoaspartate(D-aspartate) O-methyltransferase	5	≤ 0.01
P38606	-2.371	0.020	V-type proton ATPase catalytic subunit A	4	≤ 0.01
P13798	-2.618	0.006	Acylamino-acid-releasing enzyme	3	≤ 0.01
P40227	-3.349	0.001	T-complex protein 1 subunit zeta	3	≤ 0.01
E9PQW4	-1.622	0.025	Mitogen-activated protein kinase	2	≤ 0.01
O75347	-1.741	0.005	Tubulin-specific chaperone A	2	≤ 0.01
P07737	-2.689	0.004	Profilin-1	2	≤ 0.01
P13489	-2.265	0.001	Ribonuclease inhibitor	2	≤ 0.01
Q14764	-3.119	0.001	Major vault protein	2	≤ 0.01
Q96C23	-1.999	0.012	Galactose mutarotase	2	≤ 0.01
A0A0A0MS98	-3.022	0.001	Band 3 anion transport protein	1	≤ 0.05
B1AK87	-1.412	0.048	F-actin-capping protein subunit beta	1	≤ 0.05
D6RA82	-5.612	0.000	Annexin	1	≤ 0.05
D6RAY0	-2.175	0.002	Alcohol dehydrogenase class-3	1	≤ 0.01
E9PLN1	-2.688	0.033	UPF0686 protein C11orf1	1	≤ 0.05
H0Y8C6	-2.046	0.048	Importin-5 (Fragment)	1	≤ 0.01
O00515	-3.770	0.001	Ladinin-1	1	≤ 0.05
P01601	-1.796	0.015	Immunoglobulin kappa variable 1D-16	1	≤ 0.05
P26447	-2.418	0.001	Protein S100-A4	1	≤ 0.05
P49773	-2.017	0.034	Adenosine 5’-monophosphoramidase HINT1	1	≤ 0.05

With regard to proteins upregulated in RRD VH, it is worth mentioning extracellular matrix components such as interphotoreceptor matrix proteoglycan 1 and 2 (Q17R60 and Q9BZV3, respectively). Moreover, proteins involved in TGF-β signaling and pro-inflammatory cascades or angiogenic processes such as Tumor necrosis factor receptor superfamily member 12 A (Q9NP84), prosaposin (P07602), noelin-2 (O95897), vasorin (Q6EMK4) were found upregulated together with complement factors and natural inhibitors of peptidase and protease activity, such as metalloproteinase inhibitor 1 (P01033), α1-antichymotrypsin (P01011) and S-arrestin (P10523).

Accordingly, GO term analysis identified enrichment (p_adjBH_≤0.05) of terms generally associated with inflammatory and pro-fibrotic environments, in accordance with the transformation of the RRD vitreous, namely: glycosaminoglycan binding (GO:0005539), collagen-containing extracellular matrix (GO:006023), photoreceptor outer segment and cell cilium (GO:0001750 and GO:0097733, respectively), collagen (GO:0005518) (GO:0005518) and fibronectin binding (GO:0001968), enzyme inhibitor activity (GO:0004857) peptidase inhibitor and endopeptidase regulatory activity (GO:0030414 and GO:0061135, respectively). Upregulation of these terms potentially highlighted a tight interplay between unbalanced proteolysis, structural breakdown and aberrant deposition of matrix components, altered cell-to-cell contact and adhesiveness and damage to photoreceptor layers.

Conversely, proteins downregulated in RRD VH were mostly involved in proteostasis regulation and energetic/metabolic cycles, including aldehyde dehydrogenase 1A1 (P00352), transketolase (P29401 ), sorbitol dehydrogenase (Q00796), aldo-keto reductase family 1 member B1 (P15121), glutathione synthetase and reductase (P48637 and P00390, respectively), stress proteins, such as heat shock protein 90 (P07900), β-crystallins A2, A3 and B1 (P53672, P05813, P53674, respectively).

Accordingly, GO charts highlighted an enrichment in terms referable to mechanisms of proteostasis regulation and energetic metabolism such as NAD binding, (GO:0051287), protein folding chaperone (GO:0044183), oxidoreductase activities (GO:0016616, GO:0016614). Therefore, the enrichment pathways and charts associated with the downregulated proteins pointed to the dysregulation of central metabolic and proteostasis-related pathways which are suggestive of metabolic dysfunction and impaired cellular resilience in the context of RRD.

### Mining of N-termini highlights dysregulation of proteolysis in vitreous humor from RRD patients

Given the enrichment of peptidases and inhibitors observed in functional enrichment analysis of DEPs, we hypothesized that RRD patients may suffer from unbalanced extracellular proteolysis. To test this hypothesis, FragPipe/MSFragger software was used to search for semi-tryptic peptides indicative of proteolytic processing, with the specific aim of identifying N-termini and C-termini. A peptide-level FDR ≤ 0.01 was applied, and the main criteria for N-termini identification were set for the absence of a lysine (K) or arginine (R) residue at the N-terminal flanking position of the peptide. This last search parameters were set to filter out peptides probably generated by the trypsin digestion of samples, thus not representing *bona fide* endogenous N-termini.

The search parameters chosen for the analysis enabled the identification of two classes of termini: mature N-termini, which, in most cases, originate from the enzymatic processing (often intracellular) of the signal peptide during protein secretion, and neo N-termini, which typically correspond to enzymatic cleavage of mature proteins.

**Fig. 5:**
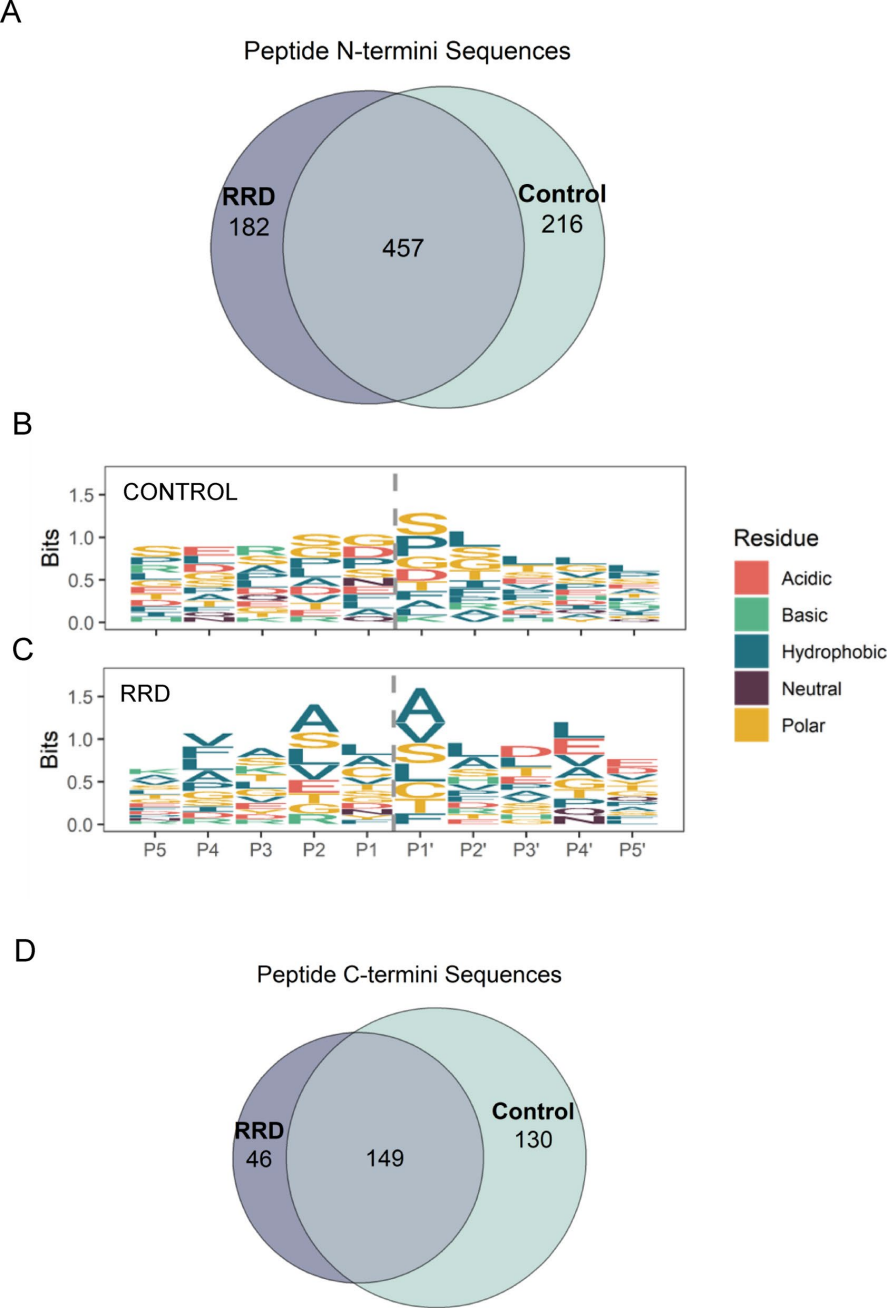
(**A**) Venn diagrams showing the distribution of N-termini identified in Control and RRD VH; Sequence logo plot generated by analyzing the aminoacidic preferences of neo N-termini of Control (**B**) and RRD (**C**). Dashed line (P1, P1’ position) indicates the cleavage site. (**D**) Venn diagrams showing the distribution of C-termini identified in Control and RRD VH.

Upon further filtering for N-termini identified in at least 3 out of 8 subjects/group (i.e., this threshold was chosen for statistical testing), the search identified a total of 457 peptides common to RRD and Control VH, 182 exclusive of RRD and 216 of Control VH samples (Fig. 5A). Neo N-termini were more represented than mature N-termini in both experimental groups.

N-termini documented as exclusive of either one of the two groups, and N-termini upregulated or downregulated (LIMMA test, Supplementary Fig. 3) were submitted (separately for each group) to TopFINDer (v. 4.1, https://topfind.clip.msl.ubc.ca/) to search for known cleavage sites of proteins identified and putative enrichment of specific proteases (the complete name of enzyme discussed below is summarized in Table 4).

**Table 4:** Lists of enzymes and their abbreviations.

Acronym	Enzyme Name
MMP2, MMP9	Matrix Metalloproteinase 2, 9 (Gelatinases)
MMP3, MMP7	Matrix Metalloproteinase 3, 7
MMP12	Matrix Metalloproteinase 12
BMP1	Bone Morphogenic Protein 1
CATH-D, -S, -L, -B	Cathepsin-D, -S, -L, -B
KLK	Kallikrein
MEP1B	Meprin 1B
ADAMTS-10, -17	A Disintegrin and Metalloproteinase with Thrombospondin motifs-10, -17
BACE	Beta-secretase 1

Several previously characterized neo N-termini were identified as downregulated (-0.57 Log_2_FC, p.mod ≤ 0.05) in the RRD/Control ratio or specific (assigned for those identified and quantified in ≥ 75% subjects) of Control VH samples (Supplementary Table 3). They included: (i) several (*n* = 15) known neo-terminal fragments of crystallin αB released by MMP9 shedding; (ii) a wide array of proteolytic fragments of additional crystallins, such as γ-crystallins (isoforms 2 − 1, 4 and S, P07315, P07320 and P22914, respectively), β-crystallin (P43320) and heat shock protein β4 (P02489); (iii) a neo N-terminus of serpin-B9; (iv) a previously unknown neo N-terminus of protein bassoon (Q9UPA5), which is a presynaptic protein and major component of photoreceptor ribbon. In this case, the potential cleavage site was mapped between ^1228^↓Tyr^1229^; (v) a neo N-terminus of osteopontin (P10451-4). This fragment was reported to be generated by cleavage of the protein at ^185^↓Ala^186^ by MMP3/MMP7^28^.

Interestingly, although the observation did not reach statistical significance, a previously unknown neo N-terminus of interphotoreceptor matrix proteoglycan 2 (IMPG2) (Q9BZV3) was found enriched in Control VH (Supplementary Table 4).

With regard to mature N-termini enriched in Control VH, it is worth documenting the identification of Wnt1 inhibitory factor (Q9Y5W5), secreted frizzled-related protein 3 (DKK3) (Q92765), spondin-1 (Q9HCB6), oligodendrocyte myelin glycoprotein (Q5SSB8), APP amyloid-beta precursor protein (P05067) and isoform 2 of fibrinogen alpha chain (A0A0S2Z3E8).

In the case of VH samples of RRD subjects, although a very limited number of upregulated N-termini (RRD/Control ratio, ≥ 0.57 Log_2_FC, p.mod ≤ 0.05) was identified, mature N-termini of several enzyme inhibitors and immune system components were documented (Supplementary Table 5): (i) C3 and PZP-like alpha-2-macroglobulin domain-containing protein 5 (P01023); (ii) α-1-antichymotrypsin (P0101); (iii) complement factor B (P00751); (iv) cystatin-3 (P01034); (v) CSF1 receptor (P07333); (vi) retinol binding protein (P10745); (vii) insulin binding proteins-4 (P22692); vi) serum amyloid A protein (P0DJI8). In this last case, the peptide was previously reported to be released by the CATL1, MMP3 and CATB cut at residues^22^↓Ser^23^.

Looking beyond the neo-termini which were specific of RRD VH group (focusing on those identified and quantified in ≥ 50% subjects/group) highlighted several known and unknown peptides serving roles for the regulation of cell adhesiveness and, most notably, angiogenesis (Supplementary Table 5), yielding insights into the proteolytic landscape of vitreous humor: (i) several proteolytic fragments of plasma proteins such as Albumin (P02768), including a previously unknown fragment generated by MEP1B and BACE2 cut between residues ^174^↓Ala^175^; (ii) serotransferrin (P02787) identified by two legumain cleavage sites mapped between residue ^601^↓Asn^602^ and ^602^↓Pro^603^; (iii) a fragment of complement C1r subcomponent (P00736), with a cleavage site located between residues ^186^↓His^187^; (iv) a fragment of clusterin (isoform 2, P10909-2) for which a cleavage fragment generated by MMP-3 and MMP12 at residues ^309^↓Met^310^ was documented; (v) calsyntenin-3 (isoform 2, Q9BQT9-2) reporting a cleavage site of ADAMTS-10 and − 17 at residues ^824^↓Val^825^; (vi) apolipoprotein A1 showing a MMP7 and MMP12 cleavage site at residues ^224^↓Ser^225^; (vii) fibronectin A - isoform2 for which two cleavage sites of MMP2, MMP8, MMP13 at residues^19^↓Ala^20^ and ^20^↓Asp^21^ were documented; (viii) basement membrane-specific heparan sulfate proteoglycan core protein (P98160). In this case, a peptide spanning across residue 4196–4221 within the C-terminal portion of the molecule and a potential cleavage site between residues ^4195^↓Glu^4196^ was identified. The fragment, called endorepellin, has been previously characterized to be released by BMP1 and to possess potent antiangiogenic activities^29^; (ix) vitronectin (P04004), with a potential cleavage site not previously reported and located between residues ^422^↓Asp^423^ and falling within the cell adhesion domain (hemopexin domain 4).

To circumstantiate further these findings, cleavage sites derived from protein termini of RRD and Control groups were analyzed by a sequence logo plot (Fig. 5B, C).

A net prevalence of polar residues, in particular glycine (G) and serine (S) was observed in P1 and P1’ position in the case of Control VH (Fig. 5B).

Conversely, RRD VH samples show a robust prevalence of polar residues such as alanine (A) and valine (V) over the same positions (Fig. 5C).

These preferences were confirmed also proceeding toward the P5 position, suggesting that different proteases may contribute to the proteolytic events of VH between RRD and Control subjects.

Mining of proteolytic events was then implemented with the search of C-termini by applying again a peptide-level FDR ≤ 0.01. In this case, the main criteria for identification were the absence of a K or R residue at the C-terminus of the peptide.

Mining of C-termini further expanded coverage of the VH degradome. In total, 149 C-termini were found as common to both the experimental groups, 46 exclusive of RRD and 130 exclusive of Control groups respectively (Fig. 5D). Whilst no significantly upregulated or downregulated C-termini were identified, in the case of Control VH, C-termini for several crystallin proteins were detected, including a previously characterized MMP9 cleavage site within crystallin αB which is complementary to one of the neo N-termini identified for the same protein and discussed above. The full list of C-termini identified is provided in Supplementary Table 6.

**Fig. 6:**
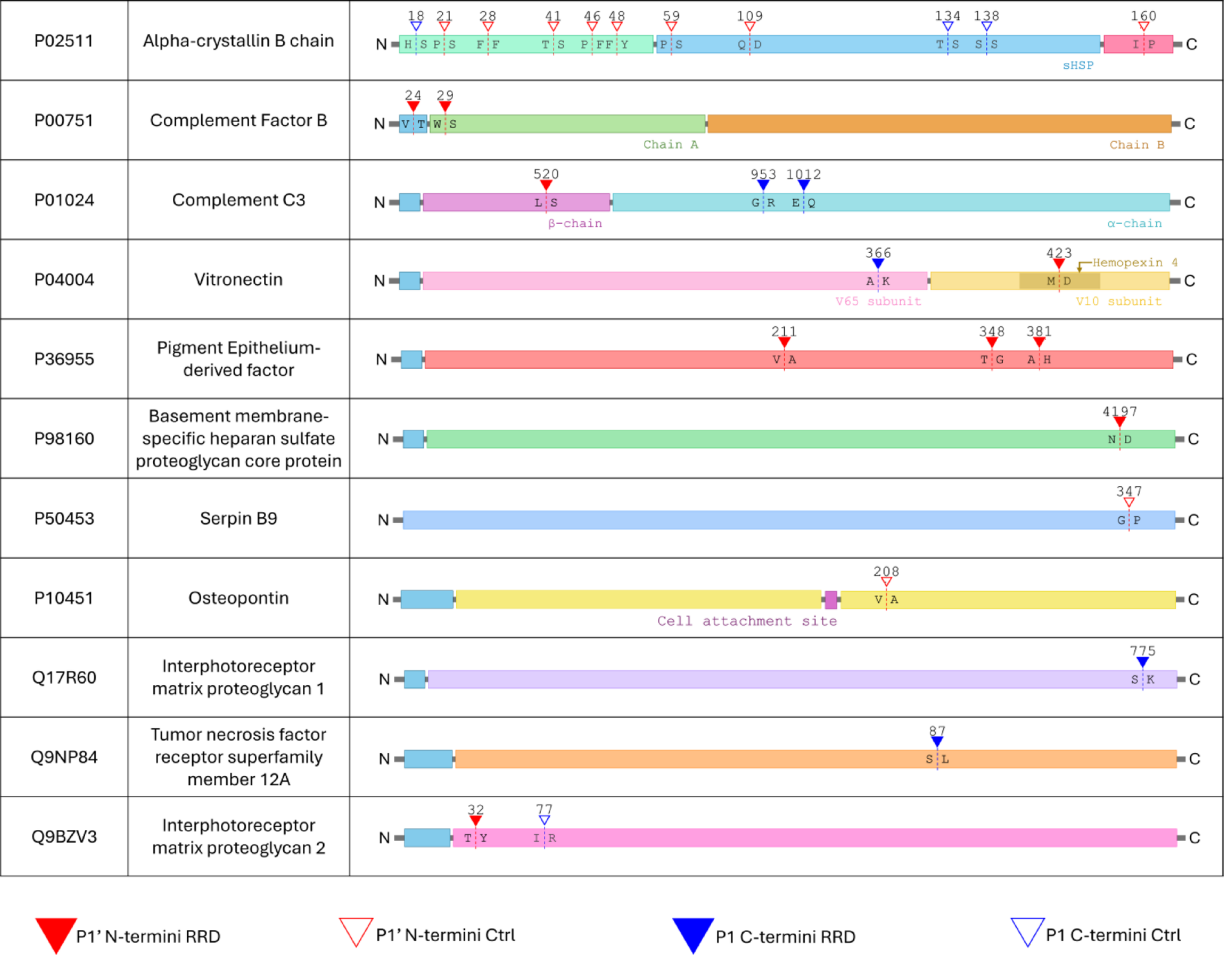
Schematic representation of the main N-terminal and C-terminal cleavage sites identified in this study.

Figure 6 shows a schematic representation of biologically relevant N-termini and C-termini cleavages sites within the domain of proteins identified.

### Western blot validation of protein identification and proteolytic processing of osteopontin and vitronectin

**Fig. 7:**
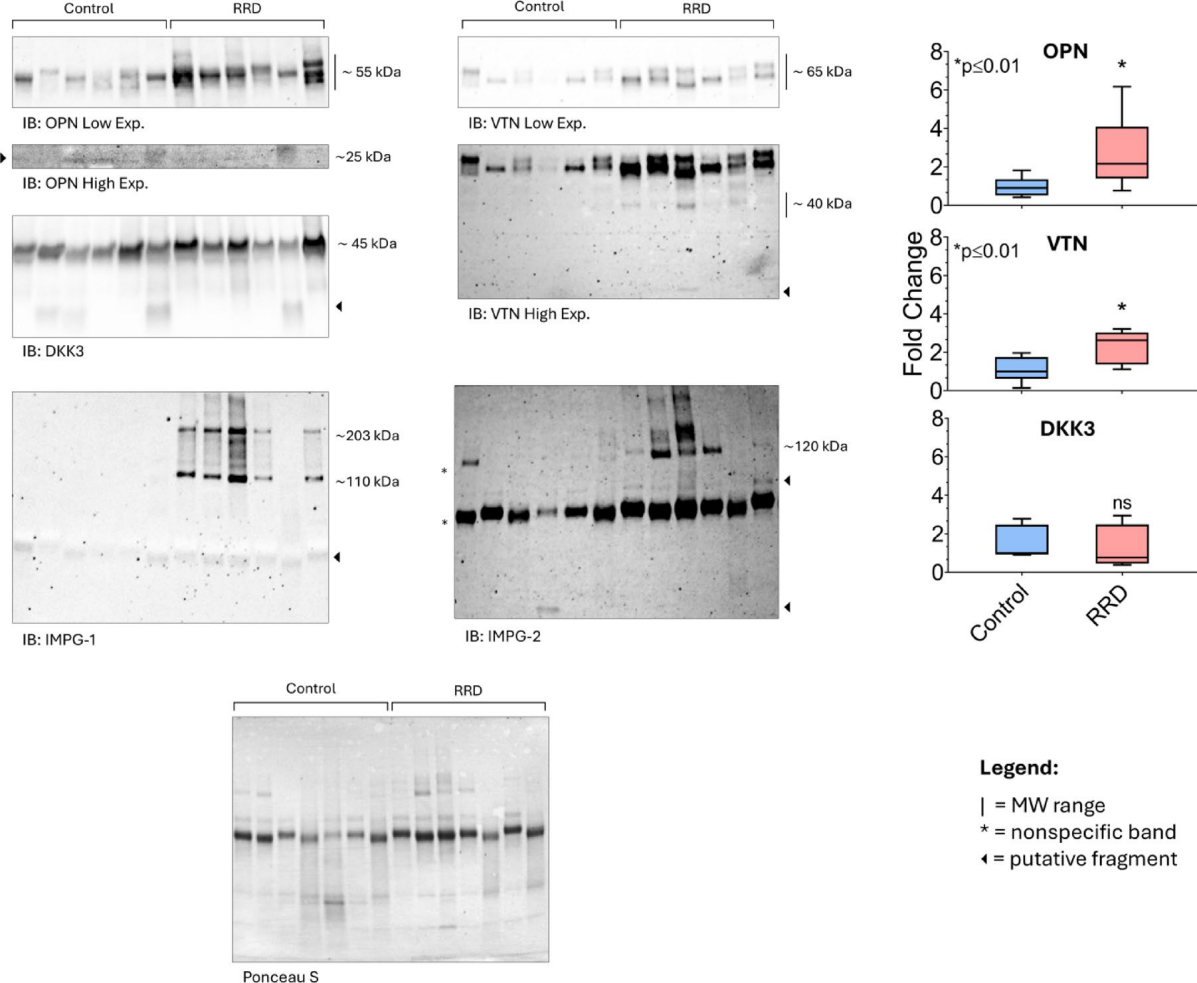
Western blotting panel showing a representative immunostaining of a panel of VH proteins of interest, namely: OPN, VTN, Interphotoreceptor Matrix Proteoglycans 1 and 2 (IMPG-1 and − 2, respectively), DKK3. Black arrows indicate putative proteolytic fragments of the protein of interest. Asterisks indicate non-specific bands. Histograms report the mean ± SD of band intensities normalized to total protein content stained by Ponceau S. All samples enrolled in the MS study (*n* = 8 for RRD and Control groups) were analyzed by Wb in technical duplicate. A nominal value of 1 was attributed to the band intensity displaying the median value of intensities distribution (across all samples). Thereafter, the fold change was computed by comparing the intensity of each band vs. this sample. Non-parametric Mann-Whitney test. Significance was set for *p* ≤ 0.05.

We validated the proteolytic processing of selected proteins using Western blot analysis, focusing on those with suitable electrophoretic properties (excluding perlecan due to its complexity) (Fig. 7).

For each sample, we loaded 5 µg of vitreous humor (VH) proteins from each sample enrolled in the MS analysis and probed them with antibodies against osteopontin (OPN), vitronectin (VTN), interphotoreceptor matrix proteoglycan 1 (IMPG1), interphotoreceptor matrix proteoglycan 2 (IMPG2), and DKK3. To enable semi-quantitative comparisons between the RRD and Control groups, and acknowledging the lack of genuine loading controls for extracellular fluid like VH, we normalized the intensity of the full-length protein bands to the total protein pattern stained by Ponceau S. Immunostaining for OPN revealed the typical multi-band pattern of the full-length protein. While a semi-quantitative analysis showed possible increase (*p* ≤ 0.01) in full-length OPN intensity in RRD VH compared to Control, a longer exposure of the Wb filters highlighted a faint ∼30 kDa band exclusively in the Control samples. This fragment was consistent with a potential cleavage site identified through N-terminal proteomic analysis and has been previously reported to result from proteolytic shedding of OPN by MMPs^28^.

Similar to OPN, the full-length VTN also turned out to increase (*p* ≤ 0.01) in the RRD VH group, compared to Controls. Notably, the anti-VTN antibody also immunostained several putative fragments in the lower molecular weight regions of the blot, observed exclusively in the RRD samples. The Wb patterns for IMPG1 and IMPG2 were unequivocal. Both full-length proteoglycans were detected solely in RRD samples, precluding quantitative comparison via histograms. Interestingly, an additional ∼50 kDa band for IMPG1 was identified across all samples (both RRD and Controls). For IMPG2, several immunoreactive bands were documented at molecular weights lower than the full-length protein and even smaller than the well-characterized fragments generated by cleavage within its sperm protein, enterokinase, and agrin (SEA) domain.

This may support the novel N-terminal cleavage observed in our proteomic data.

With regard to DKK3, whilst the band intensity of the full-length protein showed comparable between samples, different potential cleavage patterns were observed especially for Control VH samples.

## Discussion

In this pilot proteomics study, we coupled the characterization of VH proteome by LFQ DDA approaches with mining of natural N-termini (and C-termini), to introduce novel perspectives on the pathobiology of RRD based on the identification of global perturbations of the proteome and of endogenous proteolytic events. Our analysis of the VH proteome was performed on a single subject scale (rather than pooled samples) and without immunodepleting for contaminating proteins (e.g., albumin, immunoglobulins). Despite the technical challenges of identifying low-abundance proteins within a high dynamic range proteome, we identified a robust number of proteins consistent with existing literature, confirming key findings and introducing novel observations^17,18,30^. Acknowledging the inherent limitations of a relatively small sample size and its implications for statistical power, the results presented here consistently identified numerous proteins and functionally enriched pathways and terms potentially associated with RRD, compared to ERM subjects, who served as a control group, given the ethical and practical challenges of obtaining samples from genuinely healthy individuals.

However, it’s important to note that the VH of these subjects is not without its own complexities. In fact, the VH of ERM subjects is characterized by a complex interplay of inflammatory (e.g., cytokines and chemokines), fibrotic (e.g., TGF-β, Fibroblast Growth Factor, collagen deposition), and cellular proliferative pathways (e.g., pathways involved in Proliferative Vitreoretinopathy, PVR)^12,31,32^. This cautious consideration applies also to the possibility that co-presence of cataract, or prior cataract surgery, may have in the modification of the VH proteome of enrolled subjects and their inflammatory status.

In this regard, previous studies on RRD and, in particular, PVR, agreed on a multifaceted dysregulation of energetic metabolism, redox unbalance, immune system regulation and growth factors bioavailability^17,18,30^.

These processes are often initiated or exacerbated by changes in the vitreous structure itself, and thus, the data of control subjects here enrolled should be interpreted with caution when generalizing findings to a truly healthy population.

Taking the important caveats mentioned above into consideration, our pilot study identified proteins involved in TGF-β signaling (a key pro-fibrotic cytokine) and pro-inflammatory cascades (e.g., TNF receptors) as significantly enriched in the RRD VH. Several proteins involved in TGF signaling, which is the main pro-fibrotic cytokine, or in pro-inflammatory cascades, such as TNF receptors, were also identified as markedly enriched in the RRD VH proteome.

Interestingly, the polarization of macrophages towards the M2 phenotype has already been proposed as a driver of fibrosis and a major stimulus of RPE EMT during PVR; molecular cascades were effectively linked to TGFβ signaling^15^.

A deeper examination of terms of CC, MF and BP enrichment charts, generated by submitting proteins upregulated and exclusive in RRD VH to GO, highlighted further mechanisms that are very likely to underscore RRD pathogenesis.

A repertoire of terms describing mechanisms of turnover and remodeling of the extracellular matrix, with particular emphasis on the photoreceptor outer segment (interphotoreceptor matrix proteoglycans), structural proteins (collagen, fibronectin) and receptors involved in cell adhesiveness and motility (e.g., selectins) were documented significantly enriched. In addition, mechanisms or regulation of peptidase and protease activity were observed, this being in accordance with previous findings, indeed, and posing a rationale for subsequent exploration of endogenous proteolytic events^20^.

In addition to yielding insights into the global VH proteome, our dataset also allowed us to mine evidence of post-translational modification of VH proteins by proteolytic processing.

Natural endogenous N- and C-termini represent a minor fraction (~ 5%) of identifiable peptides within a proteome. Prior to the relatively recent release of ultrafast search algorithms such as MSFragger, a good coverage of these peptides was only achieved by adopting enrichment strategies, such as N-TAILS^22^. Although this enrichment procedure would almost certainly boost the numbers of identified N-termini, the informatic pipeline adopted by us is recognized to retrieve a robust coverage of the N-terminome^23^. Effectively, we here identified and quantified > 500 potential N-termini and > 300 C-termini. In most cases, they represent neo N-termini of structural and non-structural components of the VH that have not been reported before likely because the endogenous proteolysis of this fluid is an unexplored topic. However, several known cleavage sites were also identified. All together these data point out to specific events which may characterize the patho-physiological remodeling of the vitreous fluid.

To test for specific enrichment of one or more proteases, data were submitted to TopFINDer (v. 4.1, https://topfind.clip.msl.ubc.ca/)^33^, a software specifically developed to characterize proteolytic events. This search retrieved evidence of the proteolytic activities of MMP9, CATD, CATS, MMP3 and MMP7 in Control VH. A much broader array of enzymatic activities, including MMP12, MMP3, legumain, MEP1B, BMP1, were documented in RRD VH. Although none of these proteases reached the statistical threshold (q ≤ 0.05) for being considered dominant, and upregulated and downregulated N-termini were defined by p.mod values (and not q.mod), the possibility of unbalanced proteolysis in RRD VH, in our opinion, is robustly supported by four unrelated findings, namely: i) abundant VH proteins, such as crystallins, albumin and serotransferrin show different trends. Whilst neo N-termini of crystallins were significantly enriched in Control VH, this being consistent with the tryptic datasets, neo N-termini of albumin and serotransferrin were more represented in RRD VH in the absence of any obvious protein accumulation, at least based on inspection of the tryptic dataset; ii) the distribution of cleavage preferences between RRD and Control samples. Hydrophobic residues ranked first across P5 to P1’ position of neo N-termini of Control VH, whereas polar residues were mostly represented over the same sequence in the case of RRD VH neo N-termini; iii) several mature and neo N-termini of natural inhibitors of proteases, such as α1-protease inhibitor, α1-antichymotrypsin, were identified in RRD VH; iv) the pattern of neo N-termini of structural and non-structural elements was fairly different between the two experimental groups.

With respect to this last point, the total repertoire of N-termini (including RRD and Controls) suggests that proteolysis may in particular affect mechanisms of immune system regulation and cell adhesiveness/angiogenesis.

Regarding the immune system, it is worth pointing out that mature and neo-termini, either C- or N-, were identified for complement components together with a previously identified neo N-terminus of natural killer cell-enhancing factor B in RRD VH samples. Considering also the findings retrieved by the tryptic searches discussed above, it is likely that dysregulation of immune system polarization progressively develops in RRD subjects.

Discussion of proteolysis-mediated mechanisms of angiogenesis regulation, instead, is enriched with several interesting findings.

A neo N-termini stretching across the 186–193 residues of osteopontin (assigned to isoform 4 by FragPipe but shared with wild type osteopotin) and previously described to be released by a MMP3/MMP7 cut at ^185^↓Ala^186^ was identified in Control VH.

The cleavage of osteopontin was reinforced by the identification of a further C-terminus of the protein (in both Control and RRD VH) and by the Wb approach, which highlighted the presence of a faint 30 kDa fragment, but compatible with that released by MMP3 and MMP7^28^. Osteopontin is a sialoprotein serving key roles for the physiological composition of extracellular matrix^34^. Importantly, the fragment here detected was reported to have bioactive properties for cell adhesiveness, migration and angiogenesis.

Based on search on tryptic proteome and on semi-quantitative Wb data, osteopontin was documented upregulated in the RRD VH, (log_2_FC: 1.1, p.mod: 0.035, q.mod: 0.1) suggesting that these subjects may experience an increase in protein level through reduced proteolysis. In a previous study, protein levels were strongly increased in the VH of PVR subjects, compared to subjects without this complication. This finding stimulated the authors to associate osteopontin levels to RRD, probably through impaired angiogenesis^14,35^.

In coherence with this scenario, two major structural glycoproteins, such as basement membrane-specific heparan sulfate proteoglycan core protein, also called perlecan, and vitronectin were identified with neo N-termini in RRD VH.

Perlecan, is a major component of the inner limiting membrane (ILM) and VH body. Correct synthesis and deposition of this glycoprotein, as well as its turnover, are key for the homeostasis of these two ECM components of the eye^36,37^. In our study, a perlecan neo N-terminus (4196–4222 residues) was found to stretch across the C-terminal fragment of the protein. This fragment, called endorepellin, is released by BMP1 cleavage at ^4195^↓Glu^4196^ and was reported to have strong angiostatic activities by inhibiting endothelial cell adhesion to fibronectin and type I collagen^29,38^.

Vitronectin, instead, is a glycoprotein synthesized and released by photoreceptors, but also a component of plasma which play pivotal roles in ECM stabilization as well as regulation of vessels genesis and sprouting across the matrix layers^39^. Vitronectin fragments were identified as neo N- and C-termini in RRD VH and a possible fragmentation pattern of the protein was observed also by Wb, although, the abundance of the full length protein, by this approach, was higher in RRD vs. Control VH. Nevertheless, dysregulated vitronectin levels were documented by a proteomic characterization of the VH isolated from patients with blood veins occlusions, confirming that even in the retina, the glycoprotein is expected to serve roles in vessels microarchitecture and, thereby blood hemodynamics. As widely discussed throughout the manuscript, these are both patho-physiological processes considered altered in RRD and PVR subjects^40–42^.

In addition to these main findings, a proteolysis-based dysregulation of angiogenesis is supported by additional evidence, including detection of neo N-terminus of cystatin 3, which promotes angiogenesis, and of PEDF (serpin F1), in RRD VH.

Among N-termini identified, it is worth commenting the case of IMPG1 and IMPG2. These are (both) secreted and membrane glycoproteins that serve key role for the physiological composition and homeostasis of the interphotoreceptor matrix which surround the inner and outer segment of photoreceptors. Both IMPG1 and IMPG2 play vital roles in the structural organization of the inner photoreceptor matrix, the growth and maintenance of photoreceptor outer segments, and overall retinal adhesion^43^. Interestingly, the biological activity of these glycoproteins is dependent on the proteolytic activation of the immature protein by an enzymatic cut within the SEA domain. Mutations in the SEA domain that impair proteolytic processing of IMPG1 and IMPG2 are associated with retinitis pigmentosa^43^.

In this study, IMPG1 and IMPG2 were documented as robustly upregulated (Log_2_FC > 3, q.mod ≤ 0.05) in RRD VH, compared to Control VH, when the search was run using the tryptic dataset, confirming previous studies^18,21^and exclusive of RRD VH, at least for the sensitivity of the approach, by Wb. By deeply examining the PSMs, it is remarkable that, in the case of IMPG2, 12 high confident (FDR ≤ 0.01) tryptic peptides were observed in RRD VH, and only 3 in Control VH.

However, in the case of IMPG2, a neo N-terminus was instead identified as robustly downregulated in RRD VH, whilst a neo C-terminus of the same protein was exclusively identified in Control VH. The discrepancy between the tryptic and neo termini datasets envisages the possibility that accumulation of IMPG2 in RRD VH, which is likely caused by photoreceptor degeneration, is not accompanied by their proteolytic processing, which is, instead, key for their biological function. This finding, although speculative at this stage, envisages the existence of alternative proteolytic pathways for IMPG2 that are decreased or absent in RRD. These fragments that cover portion outside the SEA domain could represent products of normal physiological turnover or part of a dynamic remodeling process within the ERM which is worth being further investigated to address the impact for RRD pathology.

This study has some limitations to acknowledge. Pitfalls of present study include the previously commented relatively small sample size and the absence of longitudinal data limiting commentary on their link to disease progression, in particular PVR. Larger well-controlled studies are required to confirm these pilot findings.

An additional hypothesis that demands careful attention for the interpretation of this set of data and the additional ones that will be generated by forthcoming studies is that some of the proteins observed may display divergent compartmentalization within the same fluid. This is the case of extracellular vesicles (e.g., exosomes), which are released as a consequence of the degeneration the photoreceptors partially undergo during the acute phase of RRD^44^. Sequestration of a protein inside a vesicle shields it from digestion and identification.

Furthermore, many of the proteins here identified are heavily glycosylated under physiological conditions. Glycosylation is a PTM which critically protects them from proteolytic digestion in vivo and in vitro. Therefore, to obtain a thorough picture the glycosylation patterns should be investigated too.

## Conclusions

In conclusion, our findings introduce the working hypothesis that RRD VH is characterized by altered proteolysis of structural and non-structural components. Some of the findings here reported, in particular those supporting a proteolysis-based alteration of angiogenic processes may represent a molecular rationale for the morphological and functional abnormalities of vessels microarchitecture RRD subjects have been reported to develop. Conceptually, it is very difficult to speculate whether the alterations observed contribute to RRD onset or are a consequence of the disease. In this last case, it may be relevant to investigate whether they predispose to recurrent RRD and PVR.

Therefore, further studies on a larger cohort of samples and using additional enrichment strategies are demanded to clarify the pathogenic role of the proteolytic alterations here identified.

In this regard, another limitation of the study concerns the cohort of control subjects, who cannot be considered *bona fide* healthy subjects, and the frequency of previous cataract surgery, which can potentially introduce different patterns of pro-inflammatory proteins. However, all studies on VH are burdened by similar issues, since this fluid can be collected only through invasive surgery and a genuinely healthy population cannot be enrolled for investigating eye fluids perturbations under disease conditions.

Regarding the N-terminome, although many of the N-termini of high interest were found exclusive of RRD VH, attention should be paid to interpreting their clinical relevance.

We nonetheless believe that the approach here undertaken has the merit to pose a scientific question that may foster novel pathogenetic perspectives on RRD pathobiology and, more in general the processes of ECM remodeling within the retinal layers. Hopefully, new therapeutic targets and potential biomarkers to predict the recurrence of RRD and/or PVR can be obtained through approaches like this.

In this regard, a further clinical translation of these findings may be then obtained reproducing the data in eye fluids others than VH, such as humor aqueous, which can be harvested through minimally invasive procedures and are scalable to screening on wider populations (e.g., cataract subjects).

## Supplementary Information

Below is the link to the electronic supplementary material.

Supplementary Material 1

Supplementary Material 2

## Data Availability

“The mass spectrometry proteomics data have been deposited to the ProteomeXchange Consortium via the PRIDE partner repository with the dataset identifier PXD057155 and 10.6019/PXD057155"^45^.

## Abbreviations

AH: Aqueous humor
BCA: Bicinchoninic acid assay
BP: Biological process
CART: Classification and regression trees
CC: Cellular component
DDA: Data dependent acquisition
DEP: Differentially expressed protein
DTT: Dithiothreitol
EMT: Epithelial to mesenchymal transition
ERM: Epiretinal membranes
GO: Gene ontology
ILM: Inner limiting membrane
LFQ: Label free quantification
MF: Molecular function
OPN: Osteopontin
PCA: Principal component analysis
PD: Proteome discoverer
PPV: Pars plana vitrectomy
PTM: Post-translational modification
PVR: Proliferative vitreo-retinopathy
RRD: Rhegmatogenous retinal detachment
RPE: Retinal pigment epithelium
SEA: Sperm protein, enterokinase and agrin
TFA: Trifluoroacetic acid
Ub: Ubiquitin
UHPLC: Ultra high performance liquid chromatography
VH: Vitreous humor
VTN: Vitronectin
