# Supplementary material for: Altered pattern of proteolysis in rhegmatogenous retinal detachment by mining of N-termini datasets from vitreous humor proteome
Source: Sci Rep. 2025 Oct 14;15:35848. doi: 10.1038/s41598-025-19857-z (PMC12521590; doi:10.1038/s41598-025-19857-z)
Supplement: Supplementary file 1 — Supplementary Material 1 [file 41598_2025_19857_MOESM1_ESM.docx]

**SUPPLEMENTARY TABLES**

**Supplementary Table 1**. Proteins identified as exclusive of Control VH (based on identification in ≥50% subjects). Table reports the UniProt accession number, the Protein Description, the number of peptides (at least 1 unique peptide) and the FDR of MS identification.

| **Accession** | **Description** | **# Peptides** | **FDR** |
| --- | --- | --- | --- |
| A0A7P0T9D8 | ATP-dependent (S)-NAD(P)H-hydrate dehydratase | 2 | ≤0.01 |
| P09417 | Dihydropteridine reductase | 2 | ≤0.01 |
| P16157 | Ankyrin-1 | 2 | ≤0.01 |
| P23141 | Liver carboxylesterase 1 | 2 | ≤0.01 |
| P50991 | T-complex protein 1 subunit delta | 2 | ≤0.01 |
| Q15124 | Phosphoglucomutase-like protein 5 | 2 | ≤0.01 |
| Q6GMV2 | Histone-lysine N-trimethyltransferase SMYD5 | 2 | ≤0.01 |
| A0A1B0GVF4 | Neurexin-1-beta | 1 | ≤ 0.05 |
| A0A3B3IRL5 | Inosine-5'-monophosphate dehydrogenase (Fragment) | 1 | ≤ 0.05 |
| A0A7I2YQV1 | Transforming protein RhoA | 1 | ≤ 0.05 |
| A8MRB1 | Protein S100-B | 1 | ≤0.01 |
| C9JBI3 | Phosphoserine phosphatase (Fragment) | 1 | ≤ 0.05 |
| C9JH92 | Quinone oxidoreductase (Fragment) | 1 | ≤0.01 |
| E5RI53 | Bifunctional epoxide hydrolase 2 (Fragment) | 1 | ≤ 0.05 |
| E7EWM1 | Nuclear receptor coactivator 2 | 1 | ≤ 0.05 |
| F8W9U3 | Putative protein-lysine deacylase ABHD14B | 1 | ≤ 0.05 |
| J3QS45 | L-xylulose reductase (Fragment) | 1 | ≤ 0.05 |
| K7ERQ8 | PCAF_N domain-containing protein (Fragment) | 1 | ≤ 0.05 |
| M0QXH4 | Hyaluronan synthase 1 | 1 | ≤ 0.05 |
| M0R261 | 6-phosphogluconolactonase (Fragment) | 1 | ≤ 0.05 |
| O00487 | 26S proteasome non-ATPase regulatory subunit 14 | 1 | ≤ 0.05 |
| O95168 | NADH dehydrogenase [ubiquinone] 1 beta subcomplex subunit 4 | 1 | ≤ 0.05 |
| P05451 | Lithostathine-1-alpha | 1 | ≤ 0.05 |
| P13716 | Delta-aminolevulinic acid dehydratase | 1 | ≤ 0.05 |
| P21695 | Glycerol-3-phosphate dehydrogenase [NAD(+)], cytoplasmic | 1 | ≤ 0.05 |
| P28072 | Proteasome subunit beta type-6 | 1 | ≤ 0.05 |
| P34896 | Serine hydroxymethyltransferase, cytosolic | 1 | ≤ 0.05 |
| Q02790 | Peptidyl-prolyl cis-trans isomerase FKBP4 | 1 | ≤0.01 |
| Q5TZA2 | Rootletin | 1 | ≤ 0.05 |
| Q92820 | Gamma-glutamyl hydrolase | 1 | ≤ 0.05 |
| Q9GZM5 | Protein YIPF3 | 1 | ≤ 0.05 |

**Supplementary Table 2**. Proteins identified as exclusive of RRD VH (based on identification in ≥50% subjects). Table reports the UniProt accession number, the Protein Description, the number of peptides (at least 1 unique peptide) and the FDR of MS identification.

| **Accession** | **Description** | **# Peptides** | **FDR** |
| --- | --- | --- | --- |
| A0A0B4J1X8 | Immunoglobulin heavy variable 3-43 | 5 | ≤0.01 |
| C9JM50 | Keratin, type I cytoskeletal 19 (Fragment) | 5 | ≤0.01 |
| A0A5F9ZHM4 | L-lactate dehydrogenase | 2 | ≤0.01 |
| A0A7P0TAE1 | Endoplasmin | 2 | ≤0.01 |
| O43490 | Prominin-1 | 2 | ≤0.01 |
| P26022 | Pentraxin-related protein PTX3 | 2 | ≤0.01 |
| P35913 | Rod cGMP-specific 3',5'-cyclic phosphodiesterase subunit beta | 2 | ≤0.01 |
| Q02413 | Desmoglein-1 | 2 | ≤0.01 |
| Q96KN2 | Beta-Ala-His dipeptidase | 2 | ≤0.01 |
| Q9GZZ8 | Extracellular glycoprotein lacritin | 2 | ≤0.01 |
| A1L4H1 | Soluble scavenger receptor cysteine-rich domain-containing protein SSC5D | 1 | ≤ 0.05 |
| J3QRR0 | von Willebrand factor A domain-containing protein 1 (Fragment) | 1 | ≤ 0.05 |
| O95633 | Follistatin-related protein 3 | 1 | ≤ 0.05 |
| O95968 | Secretoglobin family 1D member 1 | 1 | ≤ 0.05 |
| P01833 | Polymeric immunoglobulin receptor | 1 | ≤ 0.05 |
| Q14982 | Opioid-binding protein/cell adhesion molecule | 1 | ≤ 0.05 |
| Q7Z7H5 | Transmembrane emp24 domain-containing protein 4 | 1 | ≤ 0.05 |
| Q93091 | Ribonuclease K6 | 1 | ≤ 0.05 |
| Q96FE5 | Leucine-rich repeat and immunoglobulin-like domain-containing nogo receptor-interacting protein 1 | 1 | ≤ 0.05 |
| Q96FE7 | Phosphoinositide-3-kinase-interacting protein 1 | 1 | ≤ 0.05 |
| Q9NZ53 | Podocalyxin-like protein 2 | 1 | ≤ 0.05 |
| Q9UGM3 | Deleted in malignant brain tumors 1 protein | 1 | ≤0.01 |

**Supplementary Table 3.** Mature and neo N-termini identified (FDR≤0.01) as exclusive of Control VH (based on identification in ≥50% subjects) or downregulated (highlighted in light blue) in RRD VH (RRD/Control ratio Log_2_FC≤0.57, p.mod≤0.05). Table reports the UniProt accession number, the protein description, the aminoacidic sequence of the peptide identified, the P10 to P1 residues, the P1’ position, protein length, previous evidence of proteolysis by a protease (when available) and peptide features (mature vs neo).

| **Accession** | **Description** | **Peptide Sequence** | **P10 to P1** | **P1' Position** | **Protein length (aa)** | **Proteases** | **N-terminal Features** |
| --- | --- | --- | --- | --- | --- | --- | --- |
| A0A075B6K4 | Immunoglobulin lambda variable 3-10 | SYELTQPPSVSVSPGQTAR | LLTFCTVSEA | 19 | 115 |  | MATURE |
| A0A087WSY6 | Immunoglobulin kappa variable 3D-15 | SNLAWYQQKPGQAPR | LSCRASQSVS | 50 | 115 |  | NEO |
| A0A0C4DH68 | Immunoglobulin kappa variable 2-24 | DIVMTQTPLSSPVTLGQPASISCR | LMLWVPGSSG | 20 | 120 |  | MATURE |
| O60575 | Serine protease inhibitor Kazal-type 4 | GKLPFSRMPICEHMVESPTCSQMSNLVCGTDGLTYTNECQLCLAR | VVDREVPVAA | 27 | 86 |  | MATURE |
| O94985-2 | CSTN1-Isoform2_HUMAN | AAQPQFVHPEHR | ANPMEHANHM | 816 | 971 |  | NEO |
|  |  | PQFVHPEHR | MEHANHMAAQ | 819 | 971 |  | NEO |
| P00352 | Aldehyde dehydrogenase, cytosolic | DLPVLLTDLK | MSSSGTP | 8 | 501 |  | NEO |
|  |  | LPVLLTDLK | MSSSGTPD | 9 | 501 |  | NEO |
|  |  | RIFVEESIYDEFVR | HQGQCCIAAS | 308 | 501 |  | NEO |
| P00558 | Cell migration-inducing gene 10 protein | PAAGSVILLENLR | GPEVEKACAN | 111 | 417 |  | NEO |
| P01034 | Cystatin-3 | DFAVGEYNKASNDMYHSR | VEEEGVRRAL | 54 | 146 | CATD | MATURE |
| P01834 | Ig kappa chain C region | SQESVTEQDSKDSTYSLSSTLTLSK | KVDNALQSGN | 52 | 107 |  | NEO |
| P02489 | Heat shock protein beta-4 Short=HspB4 | CGPKIQTGLDATHAER | SLSADGMLTF | 142 | 173 |  | NEO |
|  |  | DDHGYISREFHR | EIHGKHNERQ | 105 | 173 |  | NEO |
|  |  | DHGYISREFHR | IHGKHNERQD | 106 | 173 |  | NEO |
|  |  | DLLPFLSSTISPYYR | QFFGEGLFEY | 35 | 173 |  | NEO |
|  |  | DRDKFVIFLDVK | LDSGISEVRS | 67 | 173 |  | NEO |
|  |  | FEYDLLPFLSSTISPYYR | LFDQFFGEGL | 32 | 173 |  | NEO |
|  |  | FLSSTISPYYR | EGLFEYDLLP | 39 | 173 |  | NEO |
|  |  | FRTVLDSGISEVR | TISPYYRQSL | 53 | 173 |  | NEO |
|  |  | FVEIHGK | EDLTVKVQDD | 93 | 173 |  | NEO |
|  |  | GEGLFEYDLLPFLSSTISPYYR | YPSRLFDQFF | 28 | 173 |  | NEO |
|  |  | GPFYPSR | IQHPWFKRTL | 15 | 173 |  | NEO |
|  |  | GYISREFHR | GKHNERQDDH | 108 | 173 |  | NEO |
|  |  | KFVIFLDVK | GISEVRSDRD | 70 | 173 |  | NEO |
|  |  | KRTLGPFYPSR | MDVTIQHPWF | 11 | 173 |  | NEO |
|  |  | LFEYDLLPFLSSTISPYYR | RLFDQFFGEG | 31 | 173 |  | NEO |
|  |  | LFEYDLLPFLSSTISPYYRQSLFR | RLFDQFFGEG | 31 | 173 |  | NEO |
|  |  | LLPFLSSTISPYYR | FFGEGLFEYD | 36 | 173 |  | NEO |
|  |  | LSSTISPYYR | GLFEYDLLPF | 40 | 173 |  | NEO |
|  |  | PVSREEKPTSAPSS | LDATHAERAI | 160 | 173 |  | NEO |
|  |  | RTVLDSGISEVR | ISPYYRQSLF | 54 | 173 |  | NEO |
|  |  | SSTISPYYR | LFEYDLLPFL | 41 | 173 |  | NEO |
|  |  | STISPYYR | FEYDLLPFLS | 42 | 173 |  | NEO |
|  |  | TGLDATHAER | MLTFCGPKIQ | 148 | 173 |  | NEO |
|  |  | TISPYYR | EYDLLPFLSS | 43 | 173 |  | NEO |
|  |  | VTIQHPWFKR | MD | 3 | 173 |  | NEO |
|  |  | YYRQSLFR | LPFLSSTISP | 47 | 173 |  | NEO |
| P02511 | Alpha-crystallin B chain | SLSPFYLRPPSFLR | LESDLFPTST | 42 | 175 |  | NEO |
|  |  | DEHGFISREFHR | EVHGKHEERQ | 109 | 175 |  | NEO |
|  |  | DLFPTSTSLSPFYLRPPSFLR | QFFGEHLLES | 36 | 175 | MMP9 | NEO |
|  |  | FFGEHLLESDLFPTSTSLSPFYLRPPSFLR | FHSPSRLFDQ | 27 | 175 | MMP9 | NEO |
|  |  | FGEHLLESDLFPTSTSLSPFYLRPPSFLR | HSPSRLFDQF | 28 | 175 | MMP9 | NEO |
|  |  | FYLRPPSFLR | LFPTSTSLSP | 47 | 175 | MMP9 | NEO |
|  |  | FYLRPPSFLRAPSWFDTGLSEMR | LFPTSTSLSP | 47 | 175 | MMP9 | NEO |
|  |  | GDVIEVHGKHEERQDEHGFISR | SPEELKVKVL | 95 | 175 |  | NEO |
|  |  | IAIHHPWIR | MD | 3 | 175 | MMP9 | NEO |
|  |  | LFPTSTSLSPFYLRPPSFLR | FFGEHLLESD | 37 | 175 | MMP9 | NEO |
|  |  | PFHSPSR | HHPWIRRPFF | 16 | 175 | MMP9 | NEO |
|  |  | PITREEKPAVTAAPK | KQVSGPERTI | 160 | 175 | MMP9 | NEO |
|  |  | PITREEKPAVTAAPKK | KQVSGPERTI | 160 | 175 | MMP9 | NEO |
|  |  | SDLFPTSTSLSPFYLRPPSFLR | DQFFGEHLLE | 35 | 175 | MMP9 | NEO |
|  |  | SRLFDQFFGEHLLESDLFPTSTSLSPFYLRPPSFLR | RRPFFPFHSP | 21 | 175 | MMP9 | NEO |
|  |  | STSLSPFYLRPPSFLR | HLLESDLFPT | 41 | 175 | MMP9 | NEO |
|  |  | SWFDTGLSEMR | LRPPSFLRAP | 59 | 175 | MMP9 | NEO |
|  |  | TSLSPFYLRPPSFLR | LLESDLFPTS | 42 | 175 | MMP9 | NEO |
|  |  | TSTSLSPFYLRPPSFLR | EHLLESDLFP | 40 | 175 | MMP9 | NEO |
|  |  | YLRPPSFLR | FPTSTSLSPF | 48 | 175 | MMP9 | NEO |
| P02647 | Apolipoprotein A1 | PLGEEMRDR | KLHELQEKLS | 167 | 267 |  | NEO |
| P02671-2 | Isoform 2 of Fibrinogen alpha chain | ADSGEGDFLAEGGGVRGPR | VLSVVGTAWT | 19 | 644 |  | MATURE |
| P02766 | Transthyretin | SYSTTAVVTNPKE | YTIAALLSPY | 135 | 147 |  | MATURE |
|  |  | VAVHVFRK | DAVRGSPAIN | 48 | 147 |  | MATURE |
| P02768 | Albumin | MCTAFHDNEETFLKKYLYEIAR | PRLVRPEVDV | 146 | 609 |  | NEO |
|  |  | PFEDHVKLVNEVTEFAK | IAFAQYLQQC | 58 | 609 |  | NEO |
|  |  | PTLVEVSRNLGKVGSK | YTKKVPQVST | 444 | 609 |  | NEO |
|  |  | ASIQKFGER | VAASQAALGL | 609 | 609 |  | NEO |
|  |  | SISSKLKECCEKPLLEK | LAKYICENQD | 293 | 609 |  | NEO |
|  |  | CCHGDLLECADDRADLAK | VTDLTKVHTE | 268 | 609 |  | NEO |
|  |  | LVELVKHKPKATK | KERQIKKQTA | 552 | 609 |  | NEO |
|  |  | KTYETTLEK | YSVVLLLRLA | 374 | 609 |  | NEO |
|  |  | PELLFFAKR | IARRHPYFYA | 175 | 609 |  | NEO |
|  |  | ALVELVKHKPK | EKERQIKKQT | 552 | 609 |  | NEO |
|  |  | CFLQHKDDNPNLPR | CAKQEPERNE | 125 | 609 |  | NEO |
|  |  | FEQLGEYKFQNALLVR | QNLIKQNCEL | 419 | 609 |  | NEO |
|  |  | LGEENFK | KSEVAHRFKD | 38 | 609 |  | NEO |
|  |  | LVELVKHKPK | KERQIKKQTA | 553 | 609 |  | NEO |
|  |  | NCDKSLHTLFGDKLCTVATLR | KTCVADESAE | 85 | 609 |  | NEO |
|  |  | QRFPKAEFAEVSK | FKAWAVARLS | 245 | 609 |  | NEO |
| P02787 | Serotransferrin | APTDECKPVKWCALSHHER | RNLREGTCPE | 352 | 698 |  | NEO |
|  |  | KDSAHGFLKVPPR | SSPHGKDLLF | 314 | 698 |  | NEO |
|  |  | PSDGPSVACVKK | SFRDHMKSVI | 49 | 698 |  | NEO |
|  |  | GPSVACVKK | DHMKSVIPSD | 53 | 698 |  | NEO |
|  |  | PTDECKPVKWCALSHHERLK | NLREGTCPEA | 354 | 698 |  | NEO |
| P02790 | Hemopexin | PPTSAHGNVAEGETKPDPDVTER | CWSLAIATPL | 26 | 462 |  | NEO |
|  |  | TSAHGNVAEGETKPDPDVTER | SLAIATPLPP | 28 | 462 |  | NEO |
| P04217 | A1BG_HUMAN | ALVREDRGGR | CLAPLEGARF | 335 | 495 |  | NEO |
| P04264 | 67 kDa cytokeratin | KYEDEINKR | MQDMVEDYRN | 269 | 644 |  | NEO |
| P04406 | Peptidyl-cysteine S-nitrosylase GAPDH EC=2.6.99.- | PITIFQERDPSKIK | ENGKLVINGN | 73 | 335 | CATS | NEO |
| P05067-8 | Isoform APP751 of Amyloid-beta precursor protein | LEVPTDGNAGLLAEPQIAMFCGR | LLLAAWTARA | 17 | 751 |  | MATURE |
| P05813 | Beta-crystallin A3, isoform A1, Delta4 form Contains: | AQTNPTPGSLGPWK | ELETLPTTKM | 19 | 215 |  | NEO |
|  |  | AQTNPTPGSLGPWKITIYDQENFQGK | ELETLPTTKM | 19 | 215 |  | NEO |
|  |  | AQTNPTPGSLGPWKITIYDQENFQGKR | ELETLPTTKM | 19 | 215 |  | NEO |
|  |  | AWSGSNAYHIER | LERGEYPRWD | 98 | 215 |  | NEO |
|  |  | DYKHWREWGSHAQTSQIQSIR | YILECDHHGG | 191 | 215 |  | NEO |
|  |  | GGDYKHWR | YQYILECDHH | 189 | 215 |  | NEO |
|  |  | GGDYKHWREWGSHAQTSQIQSIR | YQYILECDHH | 189 | 215 |  | NEO |
|  |  | GPWKITIYDQENFQGK | AQTNPTPGSL | 29 | 215 |  | NEO |
|  |  | GPWKITIYDQENFQGKR | AQTNPTPGSL | 29 | 215 |  | NEO |
|  |  | GSHAQTSQIQSIRR | GGDYKHWREW | 199 | 215 |  | NEO |
|  |  | GSLGPWKITIYDQENFQGK | TKMAQTNPTP | 26 | 215 |  | NEO |
|  |  | GSLGPWKITIYDQENFQGKR | TKMAQTNPTP | 26 | 215 |  | NEO |
|  |  | HAQTSQIQSIR | DYKHWREWGS | 201 | 215 |  | NEO |
|  |  | LGPWKITIYDQENFQGK | MAQTNPTPGS | 28 | 215 |  | NEO |
|  |  | LGPWKITIYDQENFQGKR | MAQTNPTPGS | 28 | 215 |  | NEO |
|  |  | PNVSERSFDNVR | GKRMEFTSSC | 53 | 215 |  | NEO |
|  |  | PTPGSLGPWK | LPTTKMAQTN | 23 | 215 |  | NEO |
|  |  | PTPGSLGPWKITIYDQENFQGK | LPTTKMAQTN | 23 | 215 |  | NEO |
|  |  | PTPGSLGPWKITIYDQENFQGKR | LPTTKMAQTN | 23 | 215 |  | NEO |
|  |  | PWKITIYDQENFQGK | QTNPTPGSLG | 30 | 215 |  | NEO |
|  |  | PWKITIYDQENFQGKR | QTNPTPGSLG | 30 | 215 |  | NEO |
|  |  | SGAWIGYEHTSFCGQQFILER | FDNVRSLKVE | 70 | 215 |  | NEO |
|  |  | SGAWVCYQYPGYR | NNEVGSMKIQ | 165 | 215 |  | NEO |
|  |  | SKMTIFEKENFIGR | RPICSANHKE | 124 | 215 |  | NEO |
|  |  | SLGPWKITIYDQENFQGK | KMAQTNPTPG | 27 | 215 |  | NEO |
|  |  | SLGPWKITIYDQENFQGKR | KMAQTNPTPG | 27 | 215 |  | NEO |
|  |  | SMKIQSGAWVCYQYPGYR | AMGWFNNEVG | 160 | 215 |  | NEO |
|  |  | VSERSFDNVR | RMEFTSSCPN | 55 | 215 |  | NEO |
|  |  | WKITIYDQENFQGK | TNPTPGSLGP | 31 | 215 |  | NEO |
| P06727 | Apolipoprotein A4 | PLAEDVR | SAEELRQRLA | 269 | 396 |  | NEO |
| P07315 | Gamma-crystallin 2-1 | CPNLQPYFSR | QGRSYETTTD | 23 | 174 |  | NEO |
|  |  | DRFHLSEIR | ELSEDCPSIQ | 114 | 174 |  | NEO |
|  |  | SIQDRFHLSEIR | LMMELSEDCP | 111 | 174 |  | NEO |
|  |  | YLLRPQEYRR | YELPNYRGRQ | 144 | 174 |  | NEO |
| P07320 | Gamma-crystallin 4 | EIHSLNVLEGSWVLYELSNYR | SCLQDRFRFN | 120 | 174 |  | NEO |
|  |  | EIHSLNVLEGSWVLYELSNYRGR | SCLQDRFRFN | 120 | 174 |  | NEO |
|  |  | KITLYEDR | MG | 3 | 174 |  | NEO |
|  |  | LYEQPNYSGLQYFLR | SARVDSGCWM | 45 | 174 |  | NEO |
|  |  | PHSGSHR | SDSVRSCRLI | 83 | 174 |  | NEO |
|  |  | PNLQPYLSR | GRHYECSSDH | 24 | 174 |  | NEO |
|  |  | SGLQYFLR | CWMLYEQPNY | 52 | 174 |  | NEO |
|  |  | SLNVLEGSWVLYELSNYR | QDRFRFNEIH | 123 | 174 |  | NEO |
|  |  | SSDHPNLQPYLSR | RGFQGRHYEC | 20 | 174 |  | NEO |
|  |  | VLEGSWVLYELSNYRGR | FRFNEIHSLN | 126 | 174 |  | NEO |
|  |  | YEQPNYSGLQYFLR | ARVDSGCWML | 46 | 174 |  | NEO |
|  |  | YSGLQYFLR | GCWMLYEQPN | 51 | 174 |  | NEO |
| P10451-4 | Isoform D of Osteopontin | AQDLNAPSDWDSR | LNGAYKAIPV | 186 | 292 | MMP3;MMP7 | NEO |
| P13645 | Cytokeratin-10 Short=CK-10 | GGGFSSGGFSGGSFSR | LRISSSKGSL | 44 | 584 |  | NEO |
|  |  | SSGGGCFGGSSGGYGGLGGFGGGSFR | GFSGGSFSRG | 61 | 584 |  | NEO |
| P22914 | Gamma-crystallin S | ADFHTYLSR | GRRYDCDCDC | 28 | 178 |  | NEO |
|  |  | ASPAVQSFRR | EYRKPIDWGA | 166 | 178 |  | NEO |
|  |  | DFHTYLSR | RRYDCDCDCA | 29 | 178 |  | NEO |
|  |  | DFSGQMYETTEDCPSIMEQFHMR | QYKIQIFEKG | 103 | 178 |  | NEO |
|  |  | FAGYMYILPQGEYPEYQR | GTWAVYERPN | 55 | 178 |  | NEO |
|  |  | FHTYLSR | RYDCDCDCAD | 30 | 178 |  | NEO |
|  |  | FYEDKNFQGR | MSKTGTKIT | 10 | 178 |  | NEO |
|  |  | GGQYKIQIFEK | SSCRAVHLPS | 91 | 178 |  | NEO |
|  |  | GQYKIQIFEK | SCRAVHLPSG | 92 | 178 |  | NEO |
|  |  | GQYKIQIFEKGDFSGQMYETTEDCPSIMEQFHMR | SCRAVHLPSG | 92 | 178 |  | NEO |
|  |  | GTKITFYEDKNFQGR | MSKT | 5 | 178 |  | NEO |
|  |  | GTWAVYERPNFAGYMYILPQGEYPEYQR | SRCNSIKVEG | 45 | 178 |  | NEO |
|  |  | ILPQGEYPEYQR | ERPNFAGYMY | 61 | 178 |  | NEO |
|  |  | KITFYEDKNFQGR | MSKTGT | 7 | 178 |  | NEO |
|  |  | KVLEGVWIFYELPNYR | QFHMREIHSC | 131 | 178 |  | NEO |
|  |  | KVLEGVWIFYELPNYRGR | QFHMREIHSC | 131 | 178 |  | NEO |
|  |  | LLDKKEYR | ELPNYRGRQY | 151 | 178 |  | NEO |
|  |  | MYILPQGEYPEYQR | VYERPNFAGY | 59 | 178 |  | NEO |
|  |  | PAVQSFRR | RKPIDWGAAS | 168 | 178 |  | NEO |
|  |  | SIKVEGGTWAVYERPNFAGYMYILPQGEYPEYQR | DFHTYLSRCN | 39 | 178 |  | NEO |
|  |  | SIMEQFHMR | QMYETTEDCP | 117 | 178 |  | NEO |
|  |  | SPAVQSFRR | YRKPIDWGAA | 167 | 178 |  | NEO |
|  |  | TFYEDKNFQGR | MSKTGTKI | 9 | 178 |  | NEO |
|  |  | YILPQGEYPEYQR | YERPNFAGYM | 60 | 178 |  | NEO |
|  |  | YMYILPQGEYPEYQR | AVYERPNFAG | 58 | 178 |  | NEO |
| P23515 | Oligodendrocyte-myelin glycoprotein | ICPLQCICTER | LLFLTPGILC | 24 | 440 |  | MATURE |
| P26998 | Beta-B3 crystallin | SGPWLAFESR | LEKVGSIQVE | 63 | 211 |  | NEO |
| P35555 | Fibrillin-1 | ADANLEAGNVKETR | VLLASYTSHG | 25 | 2871 |  | MATURE |
| P41222 | Prostaglandin-H2 D-isomerase | DLQAAPEAQVSVQPNFQQDK | MGLALLGVLG | 19 | 190 |  | NEO |
| P43320 | Beta-crystallin Bp | DDVPSFHAHGYQEKVSSVRVQSGTWVGYQYPGYR | FTGKKMEIID | 127 | 205 |  | NEO |
|  |  | DVPSFHAHGYQEKVSSVRVQSGTWVGYQYPGYR | TGKKMEIIDD | 128 | 205 |  | NEO |
|  |  | GAPHPQVQSVR | KGDYKDSSDF | 178 | 205 |  | NEO |
|  |  | KGDYKDSSDFGAPHPQVQSVR | GYRGLQYLLE | 168 | 205 |  | NEO |
|  |  | KIILYENPNFTGK | RPIKVDSQEH | 108 | 205 |  | NEO |
|  |  | PKIIIFEQENFQGHSHELNGPCPNLK | TQAGKPQSLN | 17 | 205 |  | NEO |
|  |  | PKIIIFEQENFQGHSHELNGPCPNLKETGVEK | TQAGKPQSLN | 17 | 205 |  | NEO |
|  |  | PSFHAHGYQEK | KKMEIIDDDV | 130 | 205 |  | NEO |
|  |  | PSFHAHGYQEKVSSVR | KKMEIIDDDV | 130 | 205 |  | NEO |
|  |  | RDMQWHQR | HPQVQSVRRI | 191 | 205 |  | NEO |
|  |  | SDFGAPHPQVQSVR | LLEKGDYKDS | 175 | 205 |  | NEO |
|  |  | SFHAHGYQEKVSSVR | KMEIIDDDVP | 131 | 205 |  | NEO |
|  |  | SGTWVGYQYPGYR | QEKVSSVRVQ | 148 | 205 |  | NEO |
|  |  | SLSSLRPIKVDSQEHK | DSWTSSRRTD | 93 | 205 |  | NEO |
|  |  | SLSSLRPIKVDSQEHKIILYENPNFTGK | DSWTSSRRTD | 93 | 205 |  | NEO |
|  |  | SSLRPIKVDSQEHK | WTSSRRTDSL | 95 | 205 |  | NEO |
|  |  | TWVGYQYPGYR | KVSSVRVQSG | 150 | 205 |  | NEO |
|  |  | VPSFHAHGYQEK | GKKMEIIDDD | 129 | 205 |  | NEO |
| P50453 | Serpin B9 | PRFCADHPFLFFIR | VVAECCMESG | 347 | 376 |  | NEO |
| P53673 | Beta-A4 crystallin | DYKHFREWGSHAPTFQVQSIR | YVLECDHHSG | 172 | 196 |  | NEO |
|  |  | FTAECPSVLELGFETVR | EDGFQGRRHE | 29 | 196 |  | NEO |
|  |  | GRRHEFTAECPSVLELGFETVR | MVVWDEDGFQ | 24 | 196 |  | NEO |
|  |  | ILERGEYPSWDAWGGNTAYPAER | EHAGFQGQQY | 68 | 196 |  | NEO |
|  |  | PSVLELGFETVR | GRRHEFTAEC | 34 | 196 |  | NEO |
|  |  | PTFQVQSIR | KHFREWGSHA | 184 | 196 |  | NEO |
|  |  | SFHVHSGAWVCSQFPGYR | AMGWEGNEVG | 141 | 196 |  | NEO |
|  |  | SGDYKHFREWGSHAPTFQVQSIR | FQYVLECDHH | 170 | 196 |  | NEO |
|  |  | SVLELGFETVR | RRHEFTAECP | 35 | 196 |  | NEO |
|  |  | TAECPSVLELGFETVR | DGFQGRRHEF | 30 | 196 |  | NEO |
|  |  | WKMVVWDEDGFQGR | TLQCTKSAGP | 12 | 196 |  | NEO |
|  |  | APSLWVYGFSDR | GNTIEIQGDD | 171 | 252 |  | NEO |
|  |  | APSLWVYGFSDRVGSVK | GNTIEIQGDD | 171 | 252 |  | NEO |
|  |  | DAPSLWVYGFSDR | KGNTIEIQGD | 170 | 252 |  | NEO |
|  |  | DAPSLWVYGFSDRVGSVK | KGNTIEIQGD | 170 | 252 |  | NEO |
|  |  | DDAPSLWVYGFSDRVGSVK | FKGNTIEIQG | 169 | 252 |  | NEO |
|  |  | DTKGKGAPPAGTSPSPGTTLAPTTVPITSAK | SATVAVNPGP | 20 | 252 |  | NEO |
|  |  | FPVLATEPPK | RDKQWHLEGS | 243 | 252 |  | NEO |
|  |  | FRGEMFILEK | GPWVAFEQSN | 109 | 252 |  | NEO |
|  |  | GTTLAPTTVPITSAK | APPAGTSPSP | 36 | 252 |  | NEO |
|  |  | PGPDTKGKGAPPAGTSPSPGTTLAPTTVPITSAK | ASASATVAVN | 17 | 252 |  | NEO |
|  |  | PGTTLAPTTVPITSAK | GAPPAGTSPS | 35 | 252 |  | NEO |
|  |  | PTTVPITSAK | TSPSPGTTLA | 41 | 252 |  | NEO |
|  |  | SLWVYGFSDR | TIEIQGDDAP | 173 | 252 |  | NEO |
|  |  | SLWVYGFSDRVGSVK | TIEIQGDDAP | 173 | 252 |  | NEO |
|  |  | TKGKGAPPAGTSPSPGTTLAPTTVPITSAK | ATVAVNPGPD | 21 | 252 |  | NEO |
|  |  | TLAPTTVPITSAK | PAGTSPSPGT | 38 | 252 |  | NEO |
|  |  | TTLAPTTVPITSAK | PPAGTSPSPG | 37 | 252 |  | NEO |
|  |  | TTVPITSAK | SPSPGTTLAP | 42 | 252 |  | NEO |
|  |  | TVPITSAK | PSPGTTLAPT | 43 | 252 |  | NEO |
|  |  | WGAFQPQMQSLRR | EPGDFRHWNE | 219 | 252 |  | NEO |
|  |  | YRLVVFELENFQGR | AKAAELPPGN | 59 | 252 |  | NEO |
| P63261 | Gamma-actin | GVTHTVPIYEGYALPHAILR | TTGIVMDSGD | 158 | 375 |  | NEO |
|  |  | PSIVGRPR | AGDDAPRAVF | 32 | 375 |  | NEO |
| Q12934 | Lens fiber cell beaded-filament structural protein CP 115 Short=CP115 | GWAGATSLAALQGLGER | AAEPERPADE | 34 | 665 |  | NEO |
|  |  | PRFYVSSITAK | LVTGDANYVD | 482 | 665 |  | NEO |
|  |  | RLGELAGPEDALAR | GLRRQLDAFQ | 77 | 665 |  | NEO |
|  |  | SLAALQGLGER | PADEGWAGAT | 40 | 665 |  | NEO |
|  |  | TSLAALQGLGER | RPADEGWAGA | 39 | 665 |  | NEO |
| Q13515 | Phakinin | AVEDLGGCLVEYMAK | PAPGLERDHG | 107 | 415 |  | NEO |
|  |  | DLGGCLVEYMAK | GLERDHGAVE | 110 | 415 |  | NEO |
|  |  | PIGTGLDDILETIR | AGCELEQMDA | 263 | 415 |  | NEO |
|  |  | TGLDDILETIR | ELEQMDAPIG | 266 | 415 |  | NEO |
| Q8IZC6-3 | Isoform 3 of Collagen alpha-1(XXVII) chain | IAHPAPDS | PGSSGSAKCI | 695 | 703 |  | NEO |
| Q8N9Y4 | Protein FAM181A | PLVMASDSDVKMLLNFVNLASSDIKAALDK | PSSWKASCSG | 60 | 354 |  | NEO |
| Q9HCB6 | Spondin-1 | AFSDETLDKVPKSEGYCSR | ALALPLAAAL | 28 | 807 |  | NEO |
|  |  | LAFSDETLDKVPKSEGYCSR | LALALPLAAA | 27 | 807 |  | NEO |
| Q9NZP8 | Complement C1r subcomponent-like protein | SVLLAQELPQQLTSPGYPEPYGKGQESSTDIKAPEGFAVR | GVLQACPTRG | 40 | 487 |  | NEO |
| Q9UPA5 | Zinc finger protein 231 | YQDTTDREYGQAAQPAAEGTPASLGAAVYEEILQTSQSIVRMR | TGPRGLGSFE | 1229 | 3926 |  | NEO |
| Q9Y4C0 | Neurexin-3-alpha | LEFMGLPNQWAR | LGSLLGLCLG | 28 | 1643 |  | MATURE |

**Supplementary Table 4.** N-termini identified (FDR≤0.01) in VH (based on identification in ≥50% subjects per group) that did not reach statistical significance in the RRD/Control ratio. Table reports the UniProt accession number, the protein description, the aminoacidic sequence of the peptide identified, the P10 to P1 residues, the P1’ position, protein length, previous evidence of proteolysis by a protease (when available) and peptide features (mature vs neo).

| **Accession** | **Description** | **Peptide Sequence** | **P10 to P1** | **P1' Position** | **Protein length (aa)** | **Proteases** | **N-terminal Features** |
| --- | --- | --- | --- | --- | --- | --- | --- |
| A0A075B6P5 |  | DIVMTQSPLSLPVTPGEPASISCR |  |  |  |  | NEO |
| A0A075B6Q5 |  | EVQLVESGEGLVQPGGSLR |  |  |  |  | NEO |
| A0A075B6S9 |  | DIQLTQSPSSLSASVGDRVTITCR |  |  |  |  | NEO |
| A0A087WSY6 |  | EIVMTQSPATLSVSPGERATLSCR |  |  |  |  | NEO |
|  |  | NLAWYQQKPGQAPR |  |  |  |  | NEO |
|  |  | EIVMTQSPATLSVSPGER |  |  |  |  | NEO |
| A0A087WW87 |  | DIVMTQTPLSLPVTPGEPASISCR |  |  |  |  | NEO |
| A0A0A0MS15 |  | EVQLVESGGGLVQPGR |  |  |  |  | NEO |
|  |  | EVQLVESGGGLVQPGRSLR |  |  |  |  | NEO |
| A0A0A0MT36 |  | EIVLTQSPDFQSVTPKEKVTITCR |  |  |  |  | NEO |
|  |  | EIVLTQSPDFQSVTPK |  |  |  |  | NEO |
| A0A0B4J1U7 |  | VTPEDTAVYYCAR |  |  |  |  | NEO |
| A0A0B4J1V0 |  | EVQLVESGGGLVKPGGSLR |  |  |  |  | NEO |
| A0A0B4J1X5 |  | LVESGGGLVQPGGSLR |  |  |  |  | NEO |
|  |  | EVQLVESGGGLVQPGGSLR |  |  |  |  | NEO |
| A0A0B4J1X8 |  | EVQLVESGGVVVQPGGSLR |  |  |  |  | NEO |
| A0A0B4J2D9 |  | AIQLTQSPSSLSASVGDRVTITCR |  |  |  |  | NEO |
| A0A0C4DH38 |  | EVQLVQSGAEVK |  |  |  |  | NEO |
|  |  | EVQLVQSGAEVKKPGESLK |  |  |  |  | NEO |
| A0A0C4DH41 |  | SVTAADTAVYYCAR |  |  |  |  | NEO |
| A0A0C4DH42 |  | EVQLVESGGGLIQPGGSLR |  |  |  |  | NEO |
| A0A0C4DH69 |  | DIQLTQSPSFLSASVGDRVTITCR |  |  |  |  | NEO |
| A0A0C4DH72 |  | AIQMTQSPSSLSASVGDRVTITCR |  |  |  |  | NEO |
| A0A0C4DH73 |  | DIQMTQSPSSVSASVGDRVTITCR |  |  |  |  | NEO |
| A0A0J9YXX1 |  | EVQLVQSGAEVKKPGESLRISCK |  |  |  |  | NEO |
| O15240 | Neuroendocrine regulatory peptide-1 Short=NERP-1 Contains: | APPGRPEAQPPPLSSEHKEPVAGDAVPGPKDGSAPEVR | CLLLINGLGA | 23 | 615 |  | MATURE |
| O15240 | Neuroendocrine regulatory peptide-1 Short=NERP-1 Contains: | APPGRPEAQPPPLSSEHKEPVAGDAVPGPK | CLLLINGLGA | 23 | 615 |  | MATURE |
| O43405-2 | Isoform 2 of Cochlin | EGAAPIAITCFTR | LLLLPGPAGS | 25 | 494 |  | NEO |
| O75787-2 | Isoform 2 of Renin receptor | NEFSILKSPGSVVFR | LLALVAGVLG | 17 | 318 |  | NEO |
| O75882-2 | ATRN-Isoform2_HUMAN | AAAAAAVSGSAAAEAKECDRPCVNGGR | LLLLPCEAEA | 84 | 1272 |  | NEO |
| O94985-2 | CSTN1-Isoform2_HUMAN | MAAQPQFVHPEHR | TANPMEHANH | 815 | 971 |  | NEO |
| O94985-2 | CSTN1-Isoform2_HUMAN | AQPQFVHPEHR | NPMEHANHMA | 817 | 971 |  | NEO |
| P00439 | Phe-4-monooxygenase | GILCSALQK | ILADSINSEI | 442 | 452 |  | NEO |
| P00439 | Phe-4-monooxygenase | CSALQKIK | DSINSEIGIL | 445 | 452 |  | NEO |
| P00450 | Ferroxidase | KEKHYYIGIIETTWDYASDHGEK | LFLCSTPAWA | 20 | 1065 |  | MATURE |
| P00738 | Zonulin | VDSGNDVTDIADDGCPKPPEIAHGYVEHSVRYQCK | ALLLWGQLFA | 19 | 406 |  | MATURE |
| P00738 | Zonulin | LGGHLDAK | KNPANPVQRI | 163 | 406 | C1RL | NEO |
| P00738 | Zonulin | VDSGNDVTDIADDGCPKPPEIAHGYVEHSVR | ALLLWGQLFA | 19 | 406 |  | MATURE |
| P00738 | Zonulin | SKDYAEVGRVGYVSGWGR | NERVMPICLP | 269 | 406 |  | NEO |
| P00738 | Zonulin | KFTDHLK | VSGWGRNANF | 291 | 406 |  | NEO |
| P00738 | Zonulin | VDSGNDVTDIADDGCPKPPEIAHGYVEHSVRYQCKNYYK | ALLLWGQLFA | 19 | 406 |  | MATURE |
| P00747 | Plasmin heavy chain A Contains: | EPLDDYVNTQGASLFSVTK | LLLFLKSGQG | 20 | 810 |  | MATURE |
| P00747 | Plasmin heavy chain A Contains: | EPLDDYVNTQGASLFSVTKK | LLLFLKSGQG | 20 | 810 |  | MATURE |
| P00747 | Plasmin heavy chain A Contains: | LDDYVNTQGASLFSVTKK | LFLKSGQGEP | 22 | 810 |  | MATURE |
| P00751 | Complement factor B | SYGVKPR | CLVNLIEKVA | 302 | 764 |  | NEO |
| P00751 | Complement factor B | TPWSLARPQGSCSLEGVEIKGGSFR | LGLLSGGVTT | 26 | 764 |  | MATURE |
| P00751 | Complement factor B | ASYGVKPR | KCLVNLIEKV | 301 | 764 |  | NEO |
| P00751 | Complement factor B | TPWSLARPQGSCSLEGVEIK | LGLLSGGVTT | 26 | 764 |  | MATURE |
| P00751 | Complement factor B | WSLARPQGSCSLEGVEIKGGSFR | LLSGGVTTTP | 28 | 764 |  | MATURE |
| P00751 | Complement factor B | TTPWSLARPQGSCSLEGVEIKGGSFR | ILGLLSGGVT | 25 | 764 |  | NEO |
| P00761 |  | ATVSLPRSCAAAGTECLISGWGNTK |  |  |  |  | NEO |
| P01008 | Antithrombin-III | HGSPVDICTAKPR | LIGFWDCVTC | 33 | 464 |  | MATURE |
| P01008 | Antithrombin-III | SPVDICTAKPR | GFWDCVTCHG | 35 | 464 |  | MATURE |
| P01008 | Antithrombin-III | PMNPMCIYR | DICTAKPRDI | 48 | 464 |  | MATURE |
| P01009 | Alpha-1 protease inhibitor | GMFNIQHCK | TVKVPMMKRL | 249 | 418 |  | MATURE |
| P01009 | Alpha-1 protease inhibitor | EDPQGDAAQKTDTSHHDQDHPTFNK | LCCLVPVSLA | 25 | 418 |  | MATURE |
| P01009 | Alpha-1 protease inhibitor | IEQNTKSPLFMGK | KFNKPFVFLM | 399 | 418 | MMP7 | NEO |
| P01009 | Alpha-1 protease inhibitor | ASLHLPK | KFLENEDRRS | 308 | 418 |  | NEO |
| P01009 | Alpha-1 protease inhibitor | EDPQGDAAQKTDTSHHDQDHPTFNKITPNLAEFAFSLYR | LCCLVPVSLA | 25 | 418 |  | MATURE |
| P01009 | Alpha-1 protease inhibitor | DAAQKTDTSHHDQDHPTFNKITPNLAEFAFSLYR | PVSLAEDPQG | 30 | 418 |  | MATURE |
| P01011 | Alpha-1-antichymotrypsin | NSPLDEENLTQENQDR | GFCPAVLCHP | 26 | 423 |  | MATURE |
| P01011 | Alpha-1-antichymotrypsin | HPNSPLDEENLTQENQDR | AAGFCPAVLC | 24 | 423 |  | MATURE |
| P01011 | Alpha-1-antichymotrypsin | NRPFLMIIVPTDTQNIFFMSK | LVETRTIVRF | 396 | 423 |  | NEO |
| P01024 | C3 and PZP-like alpha-2-macroglobulin domain-containing protein 1 | SPMYSIITPNILRLESEETMVLEAHDAQGDVPVTVTVHDFPGKK | LLTHLPLALG | 23 | 1663 |  | MATURE |
| P01024 | C3 and PZP-like alpha-2-macroglobulin domain-containing protein 1 | SPMYSIITPNILRLESEETMVLEAHDAQGDVPVTVTVHDFPGK | LLTHLPLALG | 23 | 1663 |  | MATURE |
| P01024 | C3 and PZP-like alpha-2-macroglobulin domain-containing protein 1 | SPMYSIITPNILR | LLTHLPLALG | 23 | 1663 |  | MATURE |
| P01033 | Metalloproteinase inhibitor 1 | CTCVPPHPQTAFCNSDLVIR | LLWLIAPSRA | 24 | 207 |  | MATURE |
| P01034 | Cystatin-3 | VSPAAGSSPGKPPRLVGGPMDASVEEEGVRR | LLAILAVALA | 21 | 146 |  | NEO |
| P01034 | Cystatin-3 | VSPAAGSSPGKPPRLVGGPMDASVEEEGVR | LLAILAVALA | 21 | 146 |  | NEO |
| P01034 | Cystatin-3 | GKPPRLVGGPMDASVEEEGVRR | AVSPAAGSSP | 30 | 146 |  | MATURE |
| P01034 | Cystatin-3 | SPGKPPRLVGGPMDASVEEEGVR | ALAVSPAAGS | 28 | 146 |  | MATURE |
| P01034 | Cystatin-3 | SSPGKPPRLVGGPMDASVEEEGVR | VALAVSPAAG | 27 | 146 |  | MATURE |
| P01034 | Cystatin-3 | SSPGKPPRLVGGPMDASVEEEGVRR | VALAVSPAAG | 27 | 146 |  | MATURE |
| P01034 | Cystatin-3 | KPPRLVGGPMDASVEEEGVRR | VSPAAGSSPG | 31 | 146 |  | MATURE |
| P01034 | Cystatin-3 | SPGKPPRLVGGPMDASVEEEGVRR | ALAVSPAAGS | 28 | 146 |  | MATURE |
| P01034 | Cystatin-3 | KPPRLVGGPMDASVEEEGVR | VSPAAGSSPG | 31 | 146 |  | MATURE |
| P01034 | Cystatin-3 | AVPWQGTMTLSK | RKAFCSFQIY | 129 | 146 | CATD | NEO |
| P01042-2 | KNG1-Isoform2_HUMAN | GEIKEETTSHLR | GFSPFRSSRI | 394 | 427 |  | NEO |
| P01344 | Somatomedin-A | AYRPSETLCGGELVDTLQFVCGDRGFYFSRPASR | FLAFASCCIA | 25 | 180 |  | MATURE |
| P01593 | Ig kappa chain V-I region AG | NYLNWYQQKPGKAPK | ITCQASQDIS | 53 | 117 |  | NEO |
| P01597 | Ig kappa chain V-I region DEE | DIQMTQSPSSLSASVGDR | LLLWLRGARC | 23 | 117 |  | MATURE |
| P01597 | Ig kappa chain V-I region DEE | DIQMTQSPSSLSASVGDRVTITCR | LLLWLRGARC | 23 | 117 |  | MATURE |
| P01602 | Ig kappa chain V-I region HK102 | LAWYQQKPGKAPK | CRASQSISSW | 55 | 117 |  | MATURE |
| P01602 | Ig kappa chain V-I region HK102 | DIQMTQSPSTLSASVGDRVTITCR | LLLWLPGAKC | 23 | 117 |  | MATURE |
| P01602 | Ig kappa chain V-I region HK102 | WLAWYQQKPGKAPK | TCRASQSISS | 54 | 117 |  | MATURE |
| P01619 | Ig kappa chain V-III region B6 | EIVLTQSPGTLSLSPGERATLSCR | LLLWLPDTTG | 21 | 116 |  | MATURE |
| P01619 | Ig kappa chain V-III region B6 | YLAWYQQKPGQAPR | CRASQSVSSS | 53 | 116 |  | MATURE |
| P01619 | Ig kappa chain V-III region B6 | IVLTQSPGTLSLSPGERATLSCR | LLWLPDTTGE | 22 | 116 |  | MATURE |
| P01619 | Ig kappa chain V-III region B6 | IVLTQSPGTLSLSPGER | LLWLPDTTGE | 22 | 116 |  | MATURE |
| P01619 | Ig kappa chain V-III region B6 | EIVLTQSPGTLSLSPGER | LLLWLPDTTG | 21 | 116 |  | MATURE |
| P01619 | Ig kappa chain V-III region B6 | SYLAWYQQKPGQAPR | SCRASQSVSS | 52 | 116 |  | MATURE |
| P01619 | Ig kappa chain V-III region B6 | LAWYQQKPGQAPR | RASQSVSSSY | 54 | 116 |  | MATURE |
| P01714 | Ig lambda chain V-III region SH | SELTQDPAVSVALGQTVR | LTLCIGSVVS | 21 | 112 |  | MATURE |
| P01767 | Ig heavy chain V-III region BUT | EVQLVETGGGLIQPGGSLR | LVAISKGVQC | 20 | 116 |  | MATURE |
| P01772 | Ig heavy chain V-III region KOL | QVQLVESGGGVVQPGR | LVALLRGVQC | 20 | 117 |  | NEO |
| P01772 | Ig heavy chain V-III region KOL | LVESGGGVVQPGR | LLRGVQCQVQ | 23 | 117 |  | NEO |
| P01857-1 |  | PAPIEKTISK |  |  |  |  | NEO |
|  |  | PAPIEKTISKAK |  |  |  |  | NEO |
|  |  | GGTAALGCLVK |  |  |  |  | NEO |
|  |  | GTAALGCLVK |  |  |  |  | NEO |
|  |  | CPAPELLGGPSVFLFPPKPKDTLMISR |  |  |  |  | NEO |
|  |  | SGGTAALGCLVK |  |  |  |  | NEO |
|  |  | WYVDGVEVHNAK |  |  |  |  | NEO |
| P01857-1 |  | SVMHEALHNHYTQK |  |  |  |  | NEO |
|  |  | CPAPELLGGPSVFLFPPKPK |  |  |  |  | NEO |
| P01859-1 |  | FGTQTYTCNVDHKPSNTKVDKTVER |  |  |  |  | NEO |
|  |  | TYTCNVDHKPSNTK |  |  |  |  | NEO |
| P01861-1 |  | PSQEEMTKNQVSLTCLVK |  |  |  |  | NEO |
| P01876-1 |  | SPKDVLVR |  |  |  |  | NEO |
| P02647 | Apolipoprotein A1 | ALKENGGAR | LRQRLAARLE | 204 | 267 |  | NEO |
| P02647 | Apolipoprotein A1 | AARLEALKENGGAR | PYSDELRQRL | 199 | 267 |  | NEO |
| P02647 | Apolipoprotein A1 | ARLEALKENGGAR | YSDELRQRLA | 200 | 267 |  | NEO |
| P02647 | Apolipoprotein A1 | KPALEDLR | EHLSTLSEKA | 232 | 267 |  | NEO |
| P02647 | Apolipoprotein A1 | TEHLSTLSEK | ARLAEYHAKA | 221 | 267 | MMP12 | NEO |
| P02647 | Apolipoprotein A1 | DEPPQSPWDRVK | GSQARHFWQQ | 25 | 267 | BMP1 | MATURE |
| P02647 | Apolipoprotein A1 | DEPPQSPWDRVKDLATVYVDVLK | GSQARHFWQQ | 25 | 267 | BMP1 | MATURE |
| P02647 | Apolipoprotein A1 | DEPPQSPWDR | GSQARHFWQQ | 25 | 267 | BMP1 | MATURE |
| P02649 | APOE_HUMAN | LRDADDLQKR | SHLRKLRKRL | 167 | 317 |  | NEO |
| P02649 | APOE_HUMAN | KVEQAVETEPEPELR | LVTFLAGCQA | 19 | 317 |  | MATURE |
| P02654 | APOC1_HUMAN | DVSSALDKLKEFGNTLEDK | EGPAPAQGTP | 29 | 83 |  | MATURE |
| P02654 | APOC1_HUMAN | TPDVSSALDKLKEFGNTLEDK | VLEGPAPAQG | 27 | 83 |  | MATURE |
| P02656 | Apolipoprotein C3 | SEAEDASLLSFMQGYMK | LLALLASARA | 21 | 99 |  | MATURE |
| P02671-2 | FIBA-Isoform2_HUMAN | DEAGSEADHEGTHSTKRGHAK | TFESKSYKMA | 605 | 644 | CBPB2 | NEO |
| P02679-2 | FIBG-Isoform2_HUMAN | YVATRDNCCILDER | LLFLSSTCVA | 27 | 437 |  | NEO |
| P02749 | APC inhibitor | GRTCPKPDDLPFSTVVPLKTFYEPGEEITYSCKPGYVSR | SSFLCHVAIA | 20 | 345 |  | MATURE |
| P02749 | APC inhibitor | GRTCPKPDDLPFSTVVPLK | SSFLCHVAIA | 20 | 345 |  | MATURE |
| P02753 | Retinol-binding protein 4 | VHNGYCDGRSER | LCLARQYRLI | 187 | 201 |  | NEO |
| P02763 | Alpha-1-acid glycoprotein 1 | GGQEHFAHLLILR | RENGTISRYV | 111 | 201 |  | NEO |
| P02763 | Alpha-1-acid glycoprotein 1 | GGQEHFAHLLILRDTK | RENGTISRYV | 111 | 201 |  | NEO |
| P02765 | Alpha-2-Z-globulin | GSPSGEVSHPR | RHTFMGVVSL | 327 | 367 |  | NEO |
| P02765 | Alpha-2-Z-globulin | APHGPGLIYRQPNCDDPETEEAALVAIDYINQNLPWGYK | CLAQLWGCHS | 19 | 367 |  | MATURE |
| P02765 | Alpha-2-Z-globulin | VVQPSVGAAAGPVVPPCPGR | EVSHPRKTRT | 342 | 367 |  | NEO |
| P02765 | Alpha-2-Z-globulin | HGPGLIYRQPNCDDPETEEAALVAIDYINQNLPWGYK | AQLWGCHSAP | 21 | 367 |  | MATURE |
| P02766 | Transthyretin | GPTGTGESKCPLMVKVLDAVR | LAGLVFVSEA | 21 | 147 |  | MATURE |
| P02766 | Transthyretin | NVAVHVFRK | LDAVRGSPAI | 47 | 147 |  | MATURE |
| P02766 | Transthyretin | DAVRGSPAINVAVHVFR | SKCPLMVKVL | 38 | 147 |  | MATURE |
| P02766 | Transthyretin | TGTGESKCPLMVK | GLVFVSEAGP | 23 | 147 |  | MATURE |
| P02766 | Transthyretin | AVHVFRK | AVRGSPAINV | 49 | 147 |  | MATURE |
| P02766 | Transthyretin | GPTGTGESKCPLMVK | LAGLVFVSEA | 21 | 147 |  | MATURE |
| P02766 | Transthyretin | PAINVAVHVFRK | VKVLDAVRGS | 44 | 147 |  | MATURE |
| P02766 | Transthyretin | GISPFHEHAEVVFTANDSGPRR | IDTKSYWKAL | 103 | 147 |  | MATURE |
| P02766 | Transthyretin | PAINVAVHVFR | VKVLDAVRGS | 44 | 147 |  | MATURE |
| P02766 | Transthyretin | GISPFHEHAEVVFTANDSGPR | IDTKSYWKAL | 103 | 147 |  | MATURE |
| P02766 | Transthyretin | KVLDAVRGSPAINVAVHVFR | TGESKCPLMV | 35 | 147 |  | MATURE |
| P02766 | Transthyretin | PTGTGESKCPLMVK | AGLVFVSEAG | 22 | 147 |  | MATURE |
| P02766 | Transthyretin | INVAVHVFR | VLDAVRGSPA | 46 | 147 |  | MATURE |
| P02766 | Transthyretin | TIAALLSPYSYSTTAVVTNPKE | TANDSGPRRY | 126 | 147 |  | MATURE |
| P02766 | Transthyretin | ALLSPYSYSTTAVVTNPKE | DSGPRRYTIA | 129 | 147 |  | MATURE |
| P02768 | ALBU_HUMAN | VLIAFAQYLQQCPFEDHVK | DLGEENFKAL | 47 | 609 |  | NEO |
| P02768 | ALBU_HUMAN | PTLVEVSRNLGK | YTKKVPQVST | 445 | 609 |  | NEO |
| P02768 | ALBU_HUMAN | FTFHADICTLSEKER | YVPKEFNAET | 531 | 609 |  | NEO |
| P02768 | ALBU_HUMAN | STPTLVEVSR | VRYTKKVPQV | 443 | 609 |  | NEO |
| P02768 | ALBU_HUMAN | NPNLPRLVRPEVDVMCTAFHDNEETFLKK | NECFLQHKDD | 133 | 609 |  | NEO |
| P02768 | ALBU_HUMAN | LVKHKPK | QIKKQTALVE | 556 | 609 |  | NEO |
| P02768 | ALBU_HUMAN | QAADKAACLLPK | RYKAAFTECC | 194 | 609 |  | NEO |
| P02768 |  | SIQKFGER |  |  |  |  | NEO |
| P02768 | ALBU_HUMAN | MDDFAAFVEKCCK | KATKEQLKAV | 572 | 609 |  | NEO |
| P02768 |  | IQKFGER |  |  |  |  | NEO |
| P02768 | ALBU_HUMAN | FYAPELLFFAKRYK | LYEIARRHPY | 173 | 609 | BACE2;MEP1B | NEO |
| P02768 | ALBU_HUMAN | PELLFFAKRYK | IARRHPYFYA | 176 | 609 | MEP1B;BACE2 | NEO |
| P02768 | ALBU_HUMAN | CENQDSISSKLKECCEKPLLEK | DDRADLAKYI | 289 | 609 |  | NEO |
| P02768 | ALBU_HUMAN | KCASLQKFGER | GKASSAKQRL | 223 | 609 |  | NEO |
| P02768 | ALBU_HUMAN | AKTYETTLEK | DYSVVLLLRL | 374 | 609 |  | NEO |
| P02768 | ALBU_HUMAN | PHECYAKVFDEFKPLVEEPQNLIK | TLEKCCAAAD | 390 | 609 |  | NEO |
| P02768 | ALBU_HUMAN | RPEVDVMCTAFHDNEETFLKKYLYEIAR | DDNPNLPRLV | 141 | 609 |  | NEO |
| P02768 | ALBU_HUMAN | LNQLCVLHEKTPVSDRVTK | PCAEDYLSVV | 481 | 609 |  | NEO |
| P02768 | ALBU_HUMAN | FAQYLQQCPFEDHVK | ENFKALVLIA | 51 | 609 |  | NEO |
| P02768 | ALBU_HUMAN | AFHDNEETFLKKYLYEIAR | VRPEVDVMCT | 150 | 609 |  | NEO |
| P02768 | ALBU_HUMAN | DKETCFAEEGKK | AFVEKCCKAD | 587 | 609 |  | NEO |
| P02768 | ALBU_HUMAN | ETFTFHADICTLSEKER | ETYVPKEFNA | 529 | 609 |  | NEO |
| P02768 | ALBU_HUMAN | PADLPSLAADFVESKDVCKNYAEAK | CIAEVENDEM | 323 | 609 |  | NEO |
| P02768 | ALBU_HUMAN | FTECCQAADKAACLLPK | LFFAKRYKAA | 189 | 609 |  | NEO |
| P02768 | ALBU_HUMAN | SQRFPKAEFAEVSK | AFKAWAVARL | 244 | 609 |  | NEO |
| P02768 | ALBU_HUMAN | ECADDRADLAK | HTECCHGDLL | 276 | 609 |  | NEO |
| P02768 | ALBU_HUMAN | SVVLNQLCVLHEKTPVSDRVTK | KRMPCAEDYL | 478 | 609 |  | NEO |
| P02768 | ALBU_HUMAN | YFYAPELLFFAKR | YLYEIARRHP | 172 | 609 | BACE2;MEP1B | NEO |
| P02768 | ALBU_HUMAN | YSVVLLLR | LYEYARRHPD | 365 | 609 |  | NEO |
| P02768 | ALBU_HUMAN | PERNECFLQHKDDNPNLPR | EMADCCAKQE | 120 | 609 |  | NEO |
| P02768 | ALBU_HUMAN | VELVKHKPKATK | ERQIKKQTAL | 554 | 609 |  | NEO |
| P02768 | ALBU_HUMAN | VLNQLCVLHEKTPVSDRVTK | MPCAEDYLSV | 480 | 609 |  | NEO |
| P02768 | ALBU_HUMAN | KAVMDDFAAFVEKCCK | HKPKATKEQL | 569 | 609 |  | NEO |
| P02768 | ALBU_HUMAN | PELLFFAK | IARRHPYFYA | 176 | 609 | MEP1B;BACE2 | NEO |
| P02768 | ALBU_HUMAN | KETCFAEEGKK | FVEKCCKADD | 588 | 609 |  | NEO |
| P02768 | ALBU_HUMAN | VLNQLCVLHEK | MPCAEDYLSV | 480 | 609 |  | NEO |
| P02768 | ALBU_HUMAN | CAEDYLSVVLNQLCVLHEKTPVSDRVTK | CKHPEAKRMP | 472 | 609 |  | NEO |
| P02768 | ALBU_HUMAN | RPEVDVMCTAFHDNEETFLK | DDNPNLPRLV | 141 | 609 |  | NEO |
| P02768 | ALBU_HUMAN | KDLGEENFK | AHKSEVAHRF | 36 | 609 |  | NEO |
| P02768 | ALBU_HUMAN | YAEAKDVFLGMFLYEYAR | FVESKDVCKN | 343 | 609 |  | NEO |
| P02768 | ALBU_HUMAN | FLQHKDDNPNLPR | AKQEPERNEC | 126 | 609 |  | NEO |
| P02768 | ALBU_HUMAN | HDNEETFLKKYLYEIAR | PEVDVMCTAF | 152 | 609 |  | NEO |
| P02768 | ALBU_HUMAN | VELVKHKPK | ERQIKKQTAL | 554 | 609 |  | NEO |
| P02768 | ALBU_HUMAN | AAFVEKCCK | EQLKAVMDDF | 576 | 609 |  | NEO |
| P02768 | ALBU_HUMAN | PHECYAK | TLEKCCAAAD | 390 | 609 |  | NEO |
| P02768 | ALBU_HUMAN | FAQYLQQCPFEDHVKLVNEVTEFAK | ENFKALVLIA | 51 | 609 |  | NEO |
| P02768 | ALBU_HUMAN | DEMPADLPSLAADFVESK | KSHCIAEVEN | 320 | 609 |  | NEO |
| P02768 | ALBU_HUMAN | PTLVEVSR | YTKKVPQVST | 445 | 609 |  | NEO |
| P02768 | ALBU_HUMAN | TESLVNRRPCFSALEVDETYVPK | PVSDRVTKCC | 502 | 609 |  | NEO |
| P02768 | ALBU_HUMAN | TFTFHADICTLSEKER | TYVPKEFNAE | 530 | 609 |  | NEO |
| P02768 | ALBU_HUMAN | DYSVVLLLR | FLYEYARRHP | 364 | 609 |  | NEO |
| P02768 | ALBU_HUMAN | PYFYAPELLFFAKR | KYLYEIARRH | 171 | 609 | BACE2;MEP1B | NEO |
| P02768 | ALBU_HUMAN | RPEVDVMCTAFHDNEETFLKK | DDNPNLPRLV | 141 | 609 |  | NEO |
| P02768 | ALBU_HUMAN | TECCHGDLLECADDRADLAK | KLVTDLTKVH | 267 | 609 |  | NEO |
| P02768 | ALBU_HUMAN | ELVKHKPK | RQIKKQTALV | 555 | 609 |  | NEO |
| P02768 | ALBU_HUMAN | AHKSEVAHRFK | AYSRGVFRRD | 26 | 609 | PCSK5;PCSK7;KLK4;NEC1;PCSK4;FURIN;PCSK6 | MATURE |
| P02768 | ALBU_HUMAN | HEKTPVSDRVTK | SVVLNQLCVL | 488 | 609 |  | NEO |
| P02768 | ALBU_HUMAN | GERAFKAWAVAR | RLKCASLQKF | 231 | 609 |  | NEO |
| P02768 | ALBU_HUMAN | CTESLVNRRPCFSALEVDETYVPK | TPVSDRVTKC | 501 | 609 |  | NEO |
| P02768 | ALBU_HUMAN | HGDLLECADDRADLAK | DLTKVHTECC | 271 | 609 |  | NEO |
| P02768 | ALBU_HUMAN | KAWAVAR | SLQKFGERAF | 236 | 609 |  | NEO |
| P02768 | ALBU_HUMAN | AADKAACLLPK | YKAAFTECCQ | 195 | 609 |  | NEO |
| P02768 | ALBU_HUMAN | CTAFHDNEETFLKKYLYEIAR | RLVRPEVDVM | 148 | 609 |  | NEO |
| P02768 | ALBU_HUMAN | LYEIARR | DNEETFLKKY | 163 | 609 |  | NEO |
| P02768 | ALBU_HUMAN | FKAWAVAR | ASLQKFGERA | 235 | 609 |  | NEO |
| P02768 | ALBU_HUMAN | AAADPHECYAK | TYETTLEKCC | 386 | 609 |  | NEO |
| P02768 | ALBU_HUMAN | PDYSVVLLLR | MFLYEYARRH | 363 | 609 |  | NEO |
| P02768 | ALBU_HUMAN | NQLCVLHEKTPVSDRVTK | CAEDYLSVVL | 482 | 609 |  | NEO |
| P02768 | ALBU_HUMAN | VLHEKTPVSDRVTK | YLSVVLNQLC | 486 | 609 |  | NEO |
| P02768 | ALBU_HUMAN | PHECYAKVFDEFKPLVEEPQNLIKQNCELFEQLGEYK | TLEKCCAAAD | 390 | 609 |  | NEO |
| P02768 | ALBU_HUMAN | HADICTLSEKER | KEFNAETFTF | 534 | 609 |  | NEO |
| P02768 | ALBU_HUMAN | CTESLVNR | TPVSDRVTKC | 501 | 609 |  | NEO |
| P02768 | ALBU_HUMAN | QYLQQCPFEDHVKLVNEVTEFAK | FKALVLIAFA | 53 | 609 |  | NEO |
| P02768 | ALBU_HUMAN | HKSEVAHRFK | YSRGVFRRDA | 27 | 609 | PCSK5;PCSK7;KLK4;NEC1;PCSK4;FURIN;PCSK6 | MATURE |
| P02768 | ALBU_HUMAN | CKNYAEAK | AADFVESKDV | 340 | 609 |  | NEO |
| P02768 | ALBU_HUMAN | MCTAFHDNEETFLKK | PRLVRPEVDV | 147 | 609 |  | NEO |
| P02768 | ALBU_HUMAN | TPTLVEVSR | RYTKKVPQVS | 444 | 609 |  | NEO |
| P02768 | ALBU_HUMAN | KCASLQK | GKASSAKQRL | 223 | 609 |  | NEO |
| P02768 | ALBU_HUMAN | VSDRVTK | QLCVLHEKTP | 493 | 609 |  | NEO |
| P02768 | ALBU_HUMAN | IAFAQYLQQCPFEDHVKLVNEVTEFAK | GEENFKALVL | 49 | 609 |  | NEO |
| P02768 | ALBU_HUMAN | IAFAQYLQQCPFEDHVK | GEENFKALVL | 49 | 609 |  | NEO |
| P02768 | ALBU_HUMAN | NCELFEQLGEYKFQNALLVR | VEEPQNLIKQ | 415 | 609 |  | NEO |
| P02768 | ALBU_HUMAN | TFHADICTLSEKER | VPKEFNAETF | 532 | 609 |  | NEO |
| P02768 | ALBU_HUMAN | DELRDEGKASSAK | DKAACLLPKL | 207 | 609 |  | NEO |
| P02768 | ALBU_HUMAN | ECFLQHKDDNPNLPR | CCAKQEPERN | 124 | 609 |  | NEO |
| P02768 | ALBU_HUMAN | YLQQCPFEDHVK | KALVLIAFAQ | 54 | 609 |  | NEO |
| P02768 | ALBU_HUMAN | LGMFLYEYAR | KNYAEAKDVF | 351 | 609 |  | NEO |
| P02768 | ALBU_HUMAN | QLCVLHEKTPVSDRVTK | AEDYLSVVLN | 483 | 609 |  | NEO |
| P02768 | ALBU_HUMAN | TAFHDNEETFLKKYLYEIAR | LVRPEVDVMC | 149 | 609 |  | NEO |
| P02768 | ALBU_HUMAN | DELRDEGK | DKAACLLPKL | 207 | 609 |  | NEO |
| P02768 | ALBU_HUMAN | CELFEQLGEYKFQNALLVR | EEPQNLIKQN | 416 | 609 |  | NEO |
| P02768 | ALBU_HUMAN | KECCEKPLLEK | ENQDSISSKL | 300 | 609 |  | NEO |
| P02768 | ALBU_HUMAN | YFYAPELLFFAK | YLYEIARRHP | 172 | 609 | BACE2;MEP1B | NEO |
| P02768 | ALBU_HUMAN | PYFYAPELLFFAK | KYLYEIARRH | 171 | 609 | BACE2;MEP1B | NEO |
| P02768 | ALBU_HUMAN | TKKVPQVSTPTLVEVSR | KFQNALLVRY | 436 | 609 |  | NEO |
| P02768 | ALBU_HUMAN | LPSLAADFVESK | EVENDEMPAD | 326 | 609 |  | NEO |
| P02768 | ALBU_HUMAN | CVLHEKTPVSDR | DYLSVVLNQL | 485 | 609 |  | NEO |
| P02768 | ALBU_HUMAN | GEYKFQNALLVR | KQNCELFEQL | 423 | 609 |  | NEO |
| P02768 | ALBU_HUMAN | ARRHPDYSVVLLLR | VFLGMFLYEY | 359 | 609 |  | NEO |
| P02768 | ALBU_HUMAN | AETFTFHADICTLSEKER | DETYVPKEFN | 528 | 609 |  | NEO |
| P02768 | ALBU_HUMAN | CADDRADLAK | TECCHGDLLE | 277 | 609 |  | NEO |
| P02768 | ALBU_HUMAN | AQYLQQCPFEDHVK | NFKALVLIAF | 52 | 609 |  | NEO |
| P02768 | ALBU_HUMAN | CAAADPHECYAK | KTYETTLEKC | 385 | 609 |  | NEO |
| P02768 | ALBU_HUMAN | FSALEVDETYVPK | TESLVNRRPC | 512 | 609 |  | NEO |
| P02768 | ALBU_HUMAN | EVDETYVPKEFNAETFTFHADICTLSEKER | VNRRPCFSAL | 516 | 609 |  | NEO |
| P02768 | ALBU_HUMAN | YAPELLFFAKR | YEIARRHPYF | 174 | 609 | BACE2;MEP1B | NEO |
| P02768 | ALBU_HUMAN | RLVRPEVDVMCTAFHDNEETFLKK | QHKDDNPNLP | 138 | 609 |  | NEO |
| P02768 | ALBU_HUMAN | CVLHEKTPVSDRVTK | DYLSVVLNQL | 485 | 609 |  | NEO |
| P02768 | ALBU_HUMAN | YLQQCPFEDHVKLVNEVTEFAK | KALVLIAFAQ | 54 | 609 |  | NEO |
| P02768 | ALBU_HUMAN | LLECADDRADLAK | KVHTECCHGD | 274 | 609 |  | NEO |
| P02768 | ALBU_HUMAN | KAVMDDFAAFVEK | HKPKATKEQL | 569 | 609 |  | NEO |
| P02768 | ALBU_HUMAN | CTAFHDNEETFLK | RLVRPEVDVM | 148 | 609 |  | NEO |
| P02768 | ALBU_HUMAN | TLFGDKLCTVATLR | SAENCDKSLH | 92 | 609 |  | NEO |
| P02768 | ALBU_HUMAN | RLVRPEVDVMCTAFHDNEETFLK | QHKDDNPNLP | 138 | 609 |  | NEO |
| P02768 | ALBU_HUMAN | LLPKLDELRDEGK | CCQAADKAAC | 202 | 609 |  | NEO |
| P02768 | ALBU_HUMAN | QYLQQCPFEDHVK | FKALVLIAFA | 53 | 609 |  | NEO |
| P02768 | ALBU_HUMAN | FYAPELLFFAK | LYEIARRHPY | 173 | 609 | BACE2;MEP1B | NEO |
| P02768 | ALBU_HUMAN | CTAFHDNEETFLKK | RLVRPEVDVM | 148 | 609 |  | NEO |
| P02768 | ALBU_HUMAN | FYAPELLFFAKR | LYEIARRHPY | 173 | 609 | BACE2;MEP1B | NEO |
| P02768 | ALBU_HUMAN | FSALEVDETYVPKEFNAETFTFHADICTLSEKER | TESLVNRRPC | 512 | 609 |  | NEO |
| P02768 | ALBU_HUMAN | AQYLQQCPFEDHVKLVNEVTEFAK | NFKALVLIAF | 52 | 609 |  | NEO |
| P02768 | ALBU_HUMAN | AEDYLSVVLNQLCVLHEKTPVSDRVTK | KHPEAKRMPC | 473 | 609 |  | NEO |
| P02768 | ALBU_HUMAN | ELFEQLGEYKFQNALLVR | EPQNLIKQNC | 417 | 609 |  | NEO |
| P02768 | ALBU_HUMAN | AFAQYLQQCPFEDHVK | EENFKALVLI | 50 | 609 |  | NEO |
| P02768 | ALBU_HUMAN | RRHPDYSVVLLLR | FLGMFLYEYA | 360 | 609 |  | NEO |
| P02768 | ALBU_HUMAN | LFEQLGEYKFQNALLVR | PQNLIKQNCE | 418 | 609 |  | NEO |
| P02768 | ALBU_HUMAN | AEDYLSVVLNQLCVLHEKTPVSDR | KHPEAKRMPC | 473 | 609 |  | NEO |
| P02768 | ALBU_HUMAN | LQQCPFEDHVK | ALVLIAFAQY | 55 | 609 |  | NEO |
| P02768 | ALBU_HUMAN | NAETFTFHADICTLSEKER | VDETYVPKEF | 527 | 609 |  | NEO |
| P02768 | ALBU_HUMAN | HDNEETFLKK | PEVDVMCTAF | 152 | 609 |  | NEO |
| P02768 | ALBU_HUMAN | CAAADPHECYAKVFDEFKPLVEEPQNLIK | KTYETTLEKC | 385 | 609 |  | NEO |
| P02768 | ALBU_HUMAN | DDFAAFVEK | ATKEQLKAVM | 573 | 609 |  | NEO |
| P02768 | ALBU_HUMAN | RRHPYFYAPELLFFAK | FLKKYLYEIA | 168 | 609 | BACE2 | NEO |
| P02768 | ALBU_HUMAN | AAADPHECYAKVFDEFKPLVEEPQNLIK | TYETTLEKCC | 386 | 609 |  | NEO |
| P02768 | ALBU_HUMAN | DDKETCFAEEGKK | AAFVEKCCKA | 586 | 609 |  | NEO |
| P02769 |  | LQQCPFDEHVK |  |  |  |  | NEO |
| P02774-3 | Isoform 3 of Vitamin D-binding protein | SDFASNCCSINSPPLYCDSEIDAELKNIL | KELAKLVNKR | 465 | 493 |  | NEO |
| P02774-3 | Isoform 3 of Vitamin D-binding protein | SDFASNCCSINSPPLYCDSEIDAELK | KELAKLVNKR | 465 | 493 |  | NEO |
| P02787 | Serotransferrin | EEYANCHLAR | LCLDGTRKPV | 591 | 698 |  | NEO |
| P02787 | Serotransferrin | YCDLPEPR | AGWNIPIGLL | 155 | 698 |  | NEO |
| P02787 | Serotransferrin | GGKNPDPWAK | VKHQTVPQNT | 562 | 698 |  | NEO |
| P02787 | Serotransferrin | VPDKTVRWCAVSEHEATK | VCAVLGLCLA | 20 | 698 |  | MATURE |
| P02787 | Serotransferrin | WDNLKGKK | VVKKSASDLT | 460 | 698 |  | NEO |
| P02787 | Serotransferrin | PIGLLYCDLPEPR | GLGRSAGWNI | 150 | 698 |  | NEO |
| P02787 | Serotransferrin | TWDNLKGKK | AVVKKSASDL | 459 | 698 |  | NEO |
| P02787 | Serotransferrin | SDLTWDNLKGK | FAIAVVKKSA | 456 | 698 |  | NEO |
| P02787 | Serotransferrin | LFRDDTVCLAKLHDR | CLFRSETKDL | 649 | 698 |  | NEO |
| P02787 | Serotransferrin | IPSDGPSVACVKK | QSFRDHMKSV | 49 | 698 |  | NEO |
| P02787 | Serotransferrin | KYLGEEYVKAVGNLR | AKLHDRNTYE | 668 | 698 |  | NEO |
| P02787 | Serotransferrin | PEAPTDECKPVKWCALSHHER | AIRNLREGTC | 351 | 698 |  | NEO |
| P02787 | Serotransferrin | TSSLLEACTFRRP | KAVGNLRKCS | 686 | 698 |  | NEO |
| P02787 | Serotransferrin | FRDDTVCLAK | LFRSETKDLL | 650 | 698 |  | NEO |
| P02787 | Serotransferrin | PTDECKPVKWCALSHHER | NLREGTCPEA | 354 | 698 |  | NEO |
| P02787 | Serotransferrin | LFRDDTVCLAK | CLFRSETKDL | 649 | 698 |  | NEO |
| P02787 | Serotransferrin | PNHAVVTRKDK | EYANCHLARA | 602 | 698 | LGMN | NEO |
| P02787 | Serotransferrin | SLDGGFVYIAGK | KIMNGEADAM | 409 | 698 |  | NEO |
| P02787 | Serotransferrin | KMYLGYEYVTAIR | FLKVPPRMDA | 331 | 698 |  | NEO |
| P02787 | Serotransferrin | KSVIPSDGPSVACVK | TKCQSFRDHM | 46 | 698 |  | NEO |
| P02787 | Serotransferrin | IPSDGPSVACVK | QSFRDHMKSV | 49 | 698 |  | NEO |
| P02787 | Serotransferrin | PQTFYYAVAVVKK | VAEFYGSKED | 110 | 698 |  | NEO |
| P02787 | Serotransferrin | CAVSEHEATK | LAVPDKTVRW | 28 | 698 |  | MATURE |
| P02787 | Serotransferrin | KIMNGEADAMSLDGGFVYIAGK | SAETTEDCIA | 399 | 698 |  | NEO |
| P02787 | Serotransferrin | PNHAVVTR | EYANCHLARA | 602 | 698 | LGMN | NEO |
| P02787 | Serotransferrin | SGNFCLFR | HLFGSNVTDC | 635 | 698 |  | NEO |
| P02787 | Serotransferrin | CMGSGLNLCEPNNKEGYYGYTGAFR | SKKDSSLCKL | 517 | 698 |  | NEO |
| P02787 | Serotransferrin | NHAVVTR | YANCHLARAP | 603 | 698 | LGMN | NEO |
| P02787 | Serotransferrin | AVSEHEATK | AVPDKTVRWC | 29 | 698 |  | MATURE |
| P02787 | Serotransferrin | SDGPSVACVKK | FRDHMKSVIP | 51 | 698 |  | NEO |
| P02787 | Serotransferrin | DAGLVYDAYLAPNNLKPVVAEFYGSKEDPQTFYYAVAVVKK | AANEADAVTL | 82 | 698 |  | NEO |
| P02787 | Serotransferrin | MGSGLNLCEPNNKEGYYGYTGAFR | KKDSSLCKLC | 518 | 698 |  | NEO |
| P02787 | Serotransferrin | SFRDHMK | VSEHEATKCQ | 40 | 698 |  | MATURE |
| P02787 | Serotransferrin | KINHCRFDEFFSEGCAPGSK | WNIPMGLLYN | 489 | 698 |  | NEO |
| P02787 | Serotransferrin | GEEYVKAVGNLRK | HDRNTYEKYL | 671 | 698 |  | NEO |
| P02787 | Serotransferrin | SVGKIECVSAETTEDCIAK | RLKCDEWSVN | 381 | 698 |  | NEO |
| P02790 | Beta-1B-glycoprotein | HGNVAEGETKPDPDVTER | IATPLPPTSA | 32 | 462 |  | MATURE |
| P02790 | Beta-1B-glycoprotein | SVFLIKGDK | VDAAFRQGHN | 107 | 462 |  | MATURE |
| P02790 | Beta-1B-glycoprotein | LPPTSAHGNVAEGETKPDPDVTER | LCWSLAIATP | 26 | 462 |  | MATURE |
| P02790 | Beta-1B-glycoprotein | VAEGETKPDPDVTER | PLPPTSAHGN | 35 | 462 |  | MATURE |
| P02790 | Beta-1B-glycoprotein | STHHGPEYMR | GHRNGTGHGN | 247 | 462 |  | NEO |
| P02790 | Beta-1B-glycoprotein | TPLPPTSAHGNVAEGETKPDPDVTER | WSLCWSLAIA | 24 | 462 |  | MATURE |
| P03952 | Plasma prekallikrein | GCLTQLYENAFFR | FISLFATVSC | 20 | 638 |  | MATURE |
| P04196 | Histidine-proline-rich glycoprotein Short=HPRG | VSPTDCSAVEPEAEKALDLINKR | LLITLQYSCA | 19 | 525 |  | MATURE |
| P04217 | A1BG_HUMAN | LETPDFQLFKNGVAQEPVHLDSPAIKHQFLLTGDTQGR | ANVTLTCQAH | 53 | 495 |  | MATURE |
| P04217 | A1BG_HUMAN | LETPDFQLFKNGVAQEPVHLDSPAIK | ANVTLTCQAH | 53 | 495 |  | MATURE |
| P04217 | A1BG_HUMAN | LETPDFQLFK | ANVTLTCQAH | 53 | 495 |  | MATURE |
| P04433 | Ig kappa chain V-III region VG | EIVLTQSPATLSLSPGERATLSCR | LLLWLPDTTG | 21 | 115 |  | MATURE |
| P04433 | Ig kappa chain V-III region VG | IVLTQSPATLSLSPGER | LLWLPDTTGE | 22 | 115 |  | MATURE |
| P04433 | Ig kappa chain V-III region VG | EIVLTQSPATLSLSPGER | LLLWLPDTTG | 21 | 115 |  | MATURE |
| P05156 | C3B/C4B inactivator | KVTYTSQEDLVEK | LFLCFHLRFC | 19 | 583 |  | MATURE |
| P05156 | C3B/C4B inactivator | KVTYTSQEDLVEKK | LFLCFHLRFC | 19 | 583 |  | MATURE |
| P06310 | Ig kappa chain V-II region RPMI 6410 | DVVMTQSPLSLPVTLGQPASISCR | LMLWVPGSSG | 21 | 120 |  | MATURE |
| P06312 | Ig kappa chain V-IV region | DIVMTQSPDSLAVSLGERATINCK | LLLWISGAYG | 21 | 121 |  | MATURE |
| P06312 | Ig kappa chain V-IV region | DIVMTQSPDSLAVSLGER | LLLWISGAYG | 21 | 121 |  | MATURE |
| P06727 | Apolipoprotein A4 | PFATELHER | YAGDLQKKLV | 82 | 396 |  | NEO |
| P06727 | Apolipoprotein A4 | EVSADQVATVMWDYFSQLSNNAKEAVEHLQK | ALVAVAGARA | 21 | 396 |  | MATURE |
| P06727 | Apolipoprotein A4 | EVSADQVATVMWDYFSQLSNNAK | ALVAVAGARA | 21 | 396 |  | MATURE |
| P07339 | Cathepsin D light chain Contains: | LVRIPLHKFTSIR | LCLLAAPASA | 21 | 412 |  | MATURE |
| P07602 | Prosaposin | GPVLGLKECTR | LASLLGAALA | 17 | 524 |  | MATURE |
| P08571 | CD14_HUMAN | TTPEPCELDDEDFR | LLLPLVHVSA | 20 | 375 |  | MATURE |
| P08697 | Alpha-2-antiplasmin | NQEQVSPLTLLK | PLGRQLTSGP | 40 | 491 | SEPR | MATURE |
| P0C0L5 | Complement C4-B | GGNSKGTLKVLR | SLGSKINVKV | 1371 | 1744 |  | NEO |
| P0DOY3 |  | NKYAASSYLSLTPEQWK |  |  |  |  | NEO |
| P10645 | Pituitary secretory protein I Short=SP-I | LPVNSPMNKGDTEVMK | LLLCAGQVTA | 19 | 457 |  | MATURE |
| P10745 | Retinol-binding protein 3 | VWEPLQDTEHLIMDLR | GVLAPYVLRQ | 464 | 1247 |  | MATURE |
| P10745 | Retinol-binding protein 3 | LCSYFFEAEPR | PGSYSTAIPL | 796 | 1247 |  | MATURE |
| P10745 | Retinol-binding protein 3 | AVDLESLASQLTADLQEVSGDHR | AKLAQGAYRT | 676 | 1247 |  | MATURE |
| P10745 | Retinol-binding protein 3 | THLFQPSLVLDMAK | SVLLCGLAGP | 20 | 1247 |  | MATURE |
| P10745 | Retinol-binding protein 3 | GPTHLFQPSLVLDMAK | LMSVLLCGLA | 18 | 1247 |  | MATURE |
| P10745 | Retinol-binding protein 3 | FQPSLVLDMAK | LCGLAGPTHL | 23 | 1247 |  | MATURE |
| P10745 | Retinol-binding protein 3 | AEDIAHILK | VLTSSQTRGV | 239 | 1247 |  | MATURE |
| P10909-2 | Isoform 2 of Clusterin | DQTVSDNELQEMSNQGSK | LTWESGQVLG | 75 | 501 | ATS5 | NEO |
| P10909-2 | Isoform 2 of Clusterin | NEQFNWVSR | LNTSSLLEQL | 415 | 501 |  | NEO |
| P10909-2 | Isoform 2 of Clusterin | ENDRQQTHMLDVMQDHFSR | MNGDRIDSLL | 216 | 501 |  | NEO |
| P10909-2 | Isoform 2 of Clusterin | IEKTNEER | VNGVKQIKTL | 112 | 501 | MMP12 | NEO |
| P10909-2 | Isoform 2 of Clusterin | DQTVSDNELQEMSNQGSKYVNK | LTWESGQVLG | 75 | 501 | ATS5 | NEO |
| P10909-2 | Isoform 2 of Clusterin | DQTVSDNELQEMSNQGSKYVNKEIQNAVNGVK | LTWESGQVLG | 75 | 501 | ATS5 | NEO |
| P10909-2 | Isoform 2 of Clusterin | SLPHRRPHFFFPK | PQDTYHYLPF | 262 | 501 |  | NEO |
| P10909-2 | Isoform 2 of Clusterin | MDIHFHSPAFQHPPTEFIR | LEMIHEAQQA | 310 | 501 | MMP3;MMP12 | NEO |
| P10909-2 | Isoform 2 of Clusterin | SHTSDSDVPSGVTEVVVK | QYYLRVTTVA | 443 | 501 |  | NEO |
| P10909-2 | Isoform 2 of Clusterin | MFQPFLEMIHEAQQAMDIHFHSPAFQHPPTEFIR | SPYEPLNFHA | 295 | 501 |  | NEO |
| P10909-2 | Isoform 2 of Clusterin | HFHSPAFQHPPTEFIR | IHEAQQAMDI | 313 | 501 | MMP3;MMP12;MEP1B | NEO |
| P13645 | Cytokeratin-10 Short=CK-10 | SSSKGSLGGGFSSGGFSGGSFSR | GGGGVSSLRI | 37 | 584 |  | NEO |
| P14151-2 | Isoform 2 of L-selectin | WTYHYSEKPMNWQR | DFLAHHGTDC | 52 | 385 |  | NEO |
| P15586 | N-acetylglucosamine-6-sulfatase | TRRPNVVLLLTDDQDEVLGGMTPLKK | CLGVFGVAAG | 44 | 552 |  | MATURE |
| P16035 | Metalloproteinase inhibitor 2 | CSCSPVHPQQAFCNADVVIR | LATLLRPADA | 27 | 220 |  | MATURE |
| P17900 | Ganglioside GM2 activator | HLKKPSQLSSFSWDNCDEGKDPAVIR | GLLLAAPAQA | 24 | 193 |  | MATURE |
| P19022 | CDw325 | SGEIALCK | AALLQASVEA | 26 | 906 |  | MATURE |
| P19022 |  | LLLLLLL |  |  |  |  | NEO |
| P19827 | Inter-alpha-trypsin inhibitor heavy chain H1 | MSLDYGFVTPLTSMSIR | RANLSSQALQ | 596 | 911 |  | NEO |
| P23142-4 | FBLN1-Isoform4_HUMAN | DVLLEACCADGHR | LALLAAGVDA | 30 | 683 |  | NEO |
| P24592 | IBP6_HUMAN | RCPGCGQGVQAGCPGGCVEEEDGGSPAEGCAEAEGCLRR | LAASPGGALA | 28 | 240 |  | MATURE |
| P24593 | IBP5_HUMAN | LGSFVHCEPCDEK | LAAYAGPAQS | 21 | 272 | CAN1 | MATURE |
| P27797 | Endoplasmic reticulum resident protein 60 Short=ERp60 | EPAVYFKEQFLDGDGWTSR | LLGLLGLAVA | 18 | 417 |  | MATURE |
| P35527 | Cytokeratin-9 Short=CK-9 | SRSGGGGGGGLGSGGSIR | SCRQFSSSYL | 12 | 623 |  | NEO |
| P36955 | Pigment epithelium-derived factor | TGKPIKLTQVEHR | LFDSPDFSKI | 347 | 418 |  | NEO |
| P36980-2 | Isoform Short of Complement factor H-related protein 2 | EAMFCDFPK | LISRISSVGG | 19 | 243 |  | NEO |
| P41222 | Prostaglandin-H2 D-isomerase | SVSVVETDYDQYALLYSQGSKGPGEDFR | YRSPHWGSTY | 117 | 190 |  | NEO |
| P41222 | Prostaglandin-H2 D-isomerase | APEAQVSVQPNFQQDKFLGR | LLGVLGDLQA | 23 | 190 |  | MATURE |
| P41222 | Prostaglandin-H2 D-isomerase | DLQAAPEAQVSVQPNFQQDKFLGR | MGLALLGVLG | 19 | 190 |  | NEO |
| P41222 | Prostaglandin-H2 D-isomerase | APEAQVSVQPNFQQDK | LLGVLGDLQA | 23 | 190 |  | MATURE |
| P41222 | Prostaglandin-H2 D-isomerase | PEAQVSVQPNFQQDKFLGR | LGVLGDLQAA | 24 | 190 |  | MATURE |
| P41222 | Prostaglandin-H2 D-isomerase | AQVSVQPNFQQDKFLGR | VLGDLQAAPE | 26 | 190 |  | MATURE |
| P41222 | Prostaglandin-H2 D-isomerase | CKAQGFTEDTIVFLPQTDKCMTEQ | AELKEKFTAF | 167 | 190 |  | NEO |
| P41222 | Prostaglandin-H2 D-isomerase | EAQVSVQPNFQQDKFLGR | GVLGDLQAAP | 25 | 190 |  | MATURE |
| P49190 | PTH2R_HUMAN | AFVAAWAVAR | FILIGWGFPA | 289 | 550 |  | MATURE |
| P51693-2 | Isoform 2 of Amyloid-like protein 1 | SLAGGSPGAAEAPGSAQVAGLCGR | LLLRAQPAIG | 39 | 651 |  | NEO |
| P61626 | Lysozyme C | KVFERCELAR | LVLLSVTVQG | 19 | 148 |  | MATURE |
| P61916 | Epididymal secretory protein E1 | EPVQFKDCGSVDGVIKEVNVSPCPTQPCQLSK | LLALSTAAQA | 20 | 151 |  | MATURE |
| P61916 | Epididymal secretory protein E1 | EPVQFKDCGSVDGVIK | LLALSTAAQA | 20 | 151 |  | MATURE |
| P80748 | Ig lambda chain V-III region LOI | YVLTQPPSVSVAPGQTAR | LSHCTGSVTS | 21 | 117 |  | NEO |
| P81605-2 | Isoform 2 of Dermcidin | YDPEAASAPGSGNPCHEASAAQKENAGEDPGLAR | TALAGALVCA | 20 | 121 | GRAM | NEO |
| Q06481 | APPH | DVKEMIFNAER | DENMVIDETL | 660 | 763 |  | NEO |
| Q06481 | APPH | GYIEALAANAGTGFAVAEPQIAMFCGK | GLTAPALALA | 32 | 763 |  | MATURE |
| Q14624 | Inter-alpha-trypsin inhibitor heavy chain H4 | EKNGIDIYSLTVDSR | LLAIHQTTTA | 29 | 930 |  | MATURE |
| Q15582 | Transforming growth factor-beta-induced protein ig-h3 | GPAKSPYQLVLQHSR | LALGPAATLA | 24 | 683 |  | MATURE |
| Q16270 | Insulin-like growth factor-binding protein 7 | SSSDTCGPCEPASCPPLPPLGCLLGETR | LLLLLPLSSS | 27 | 282 |  | MATURE |
| Q17R60 | Interphotoreceptor matrix proteoglycan of 150 kDa Short=IPM-150 | TKDISINIYHSETK | VFWIFLQVQG | 21 | 797 |  | MATURE |
| Q86UX2 | Inter-alpha-trypsin inhibitor heavy chain H5 | PHFVVDFPLSR | KISKTSVDGD | 682 | 942 |  | MATURE |
| Q8IZJ3-2 | Isoform 2 of C3 and PZP-like alpha-2-macroglobulin domain-containing protein 8 | AQPQAPGYLIAAPSVFR | LLSARDGVRA | 26 | 1815 |  | NEO |
| Q8N3Z0 | PRS35_HUMAN | SEMEWDFMWHLR | FTPGWTLIDG | 21 | 413 |  | MATURE |
| Q8NCX0-2 | Isoform 2 of Coiled-coil domain-containing protein 150 | LDEANFRSVEVSR | QREVAELKKA | 344 | 417 |  | NEO |
| Q92765 | Secreted frizzled-related protein 3 | AACEPVRIPLCK | CLLRVPGARA | 33 | 325 |  | MATURE |
| Q92823-2 |  | LLLLLLLL |  |  |  |  | NEO |
| Q96FE7-2 | P3IP1-Isoform2_HUMAN | YGSGGCFWDNGHLYREDQTSPAPGLR | LVSNMLLAEA | 20 | 234 |  | NEO |
| Q96FE7-2 | P3IP1-Isoform2_HUMAN | SGGCFWDNGHLYREDQTSPAPGLR | SNMLLAEAYG | 22 | 234 |  | NEO |
| Q96IY4 | Carboxypeptidase U Short=CPU | FQSGQVLAALPR | VLFCEQHVFA | 23 | 423 |  | MATURE |
| Q96PD5 | N-acetylmuramoyl-L-alanine amidase | SLPLLMDSVIQALAELEQKVPAAKTR | LLLWSDPGTA | 22 | 576 |  | MATURE |
| Q96PD5 | N-acetylmuramoyl-L-alanine amidase | SLPLLMDSVIQALAELEQKVPAAK | LLLWSDPGTA | 22 | 576 |  | MATURE |
| Q9BZV3 | Interphotoreceptor matrix proteoglycan of 200 kDa Short=IPM 200 | TYLSIEEIQEPK | EGDFPSLTAQ | 31 | 1241 |  | NEO |
| Q9H0Q3-2 | FXYD6-Isoform2_HUMAN | SAAEKEKEMDPFHYDYQTLR | CSLLAPMVLA | 19 | 112 |  | NEO |
| Q9HBM0-2 | VEZA-Isoform2_HUMAN | EQISEEEAHNFTDGFSLPALKVLFQLWVAQSSEFFRR | FKELGLGLSE | 264 | 569 |  | NEO |
| Q9HCB6 | Spondin-1 | LAFSDETLDKVPK | LALALPLAAA | 27 | 807 |  | NEO |
| Q9UBP4 | Dickkopf-related protein 3 | LLFPVCTPLPVEGELCHDPASR | PGLCCAFQRG | 226 | 350 |  | NEO |
| Q9UHI8 | METH-1 | LGRPSEEDEELVVPELERAPGHGTTR | AAALLAVSDA | 50 | 967 |  | MATURE |
| Q9UN30 | Sex comb on midleg-like protein 1 | KPEFVNKEPNIVSDASCNTEEQLK | DNTILYAYET | 33 | 329 |  | NEO |
| Q9Y287-2 | Isoform 2 of Integral membrane protein 2B | SNCFAIR | TIKGIQKREA | 140 | 160 |  | NEO |
| Q9Y2I7 | 1-phosphatidylinositol 3-phosphate 5-kinase | KLRGGSDYELAR | GCPQHLGCTI | 840 | 2098 |  | NEO |
| Q9Y5W5 | Wnt inhibitory factor 1 | GPPQEESLYLWIDAHQAR | LCLLALRAEA | 29 | 379 |  | MATURE |
| Q9Y5Y7 | Lymphatic vessel endothelial hyaluronic acid receptor 1 | SLRAEELSIQVSCR | IWTTRLLVQG | 24 | 322 |  | MATURE |
| Q9Y6R7 | IgGFc-binding protein | PHYTTFDGRR | STAVCRAQGD | 478 | 5405 |  | MATURE |
| Q9Y6R7 | IgGFc-binding protein | PHYHSFDGR | YEATCWLWGD | 1679 | 5405 |  | NEO |

**Supplementary Table 5.** Mature and neo N-termini identified (FDR≤0.01) as exclusive of RRD VH (based on identification in ≥50% subjects) or upregulated (highlight in light red) in RRD VH (RRD/Control ratio Log_2_FC≤0.57, p.mod≤0.05). Table reports the UniProt accession number, the protein description, the aminoacidic sequence of the peptide identified, the P10 to P1 residues, the P1’ position, protein length, previous evidence of proteolysis by a protease (when available) and peptide features (mature vs neo).

| **Accession** | **Description** | **Peptide Sequence** | **P10 to P1** | **P1' Position** | **Protein length (aa)** | **Proteases** | **N-terminal Features** |
| --- | --- | --- | --- | --- | --- | --- | --- |
| O00292-2 | Isoform 2 of Left-right determination factor 2 | LTEEQLLGSLLR | VLPLAGPGAA | 22 | 332 |  | NEO |
| O75882-2 | ATRN-Isoform2_HUMAN | AAAAAAVSGSAAAEAK | LLLLPCEAEA | 84 | 1272 |  | NEO |
| P00450 | Ferroxidase | TGDKVYVHLK | GFLGPIIKAE | 101 | 1065 |  | MATURE |
| P00736 | Complement C1r subcomponent | HSCQAECSSELYTEASGYISSLEYPR | RPGYELQEDT | 187 | 705 |  | NEO |
| P00751 | Complement factor B | SLARPQGSCSLEGVEIKGGSFR | LSGGVTTTPW | 29 | 764 |  | MATURE |
|  |  | TTPWSLARPQGSCSLEGVEIK | ILGLLSGGVT | 25 | 764 |  | NEO |
| P01009 | Alpha-1 protease inhibitor | GADLSGVTEEAPLKLSK | QLGITKVFSN | 339 | 418 |  | NEO |
| P01009 | Alpha-1 protease inhibitor | LAEFAFSLYR | HPTFNKITPN | 54 | 418 |  | MATURE |
| P01011 | Alpha-1-antichymotrypsin | HPNSPLDEENLTQENQDRGTHVDLGLASANVDFAFSLYK | AAGFCPAVLC | 24 | 423 |  | MATURE |
| P01023 | C3 and PZP-like alpha-2-macroglobulin domain-containing protein 5 | SVSGKPQYMVLVPSLLHTETTEK | LLLVLLPTDA | 24 | 1474 |  | MATURE |
|  |  | ILRLESEETMVLEAHDAQGDVPVTVTVHDFPGK | SPMYSIITPN | 33 | 1663 |  | MATURE |
|  |  | SITTDFIPSFR | PGQDLVVLPL | 520 | 1663 |  | MATURE |
| P01034 | Cystatin-3 | CSFQIYAVPWQGTMTLSK | DQPHLKRKAF | 123 | 146 |  | NEO |
|  |  | GKPPRLVGGPMDASVEEEGVR | AVSPAAGSSP | 30 | 146 |  | MATURE |
|  |  | QIYAVPWQGTMTLSK | HLKRKAFCSF | 126 | 146 | CATD | NEO |
|  |  | RLVGGPMDASVEEEGVR | AAGSSPGKPP | 34 | 146 | MMP2;MEP1A;MEP1B;ELNE | MATURE |
|  |  | SFQIYAVPWQGTMTLSK | QPHLKRKAFC | 124 | 146 |  | NEO |
| P01597 | Ig kappa chain V-I region DEE | YLNWYQQKPGKAPK | TCRASQSISS | 54 | 117 |  | NEO |
| P01602 | Ig kappa chain V-I region HK102 | DIQMTQSPSTLSASVGDR | LLLWLPGAKC | 23 | 117 |  | MATURE |
| P01699 | Ig lambda chain V-I region VOR | TVNWYQQLPGTAPK | SGSSSNIGSN | 52 | 117 |  | NEO |
| P01703 | Ig lambda chain V-I region NEWM | QSVLTQPPSVSGAPGQR | LLAHCTGSWA | 20 | 118 |  | NEO |
| P01718 | Ig lambda chain V-IV region Kern | YELTQPSSVSVSPGQTAR | LILCTVSVAS | 21 | 113 |  | MATURE |
| P01721 | Ig lambda chain V-VI region AR | NFMLTQPHSVSESPGK | LLAHCTGSWA | 20 | 117 |  | MATURE |
| P01834 | Ig kappa chain C region | CLLNNFYPR | QLKSGTASVV | 27 | 107 |  | NEO |
|  |  | EVTHQGLSSPVTK | DYEKHKVYAC | 88 | 107 |  | NEO |
| A0A0B4J1V6 | Ig heavy variable 3-73 | EVQLVESGGGLVQPGGSLK | LVAILKGVQCE | 20 | 38 |  | NEO |
| P02647 | Apolipoprotein A1 | STLSEKAKPALEDLR | EYHAKATEHL | 225 | 267 | MMP12;MMP7 | NEO |
| P02671-2 | FIBA-Isoform2_HUMAN | ADSGEGDFLAEGGGVR | VLSVVGTAWT | 20 | 644 | MMP8;MMP12;MMP13 | NEO |
|  |  | DSGEGDFLAEGGGVR | LSVVGTAWTA | 21 | 644 | MMP8;MMP12;MMP13 | NEO |
| P02765 | Alpha-2-Z-globulin | SLGSPSGEVSHPR | DLRHTFMGVV | 325 | 367 |  | NEO |
| P02765 | Alpha-2-Z-globulin | SPSGEVSHPR | HTFMGVVSLG | 328 | 367 |  | NEO |
| P02766 | Transthyretin | STTAVVTNPKE | IAALLSPYSY | 136 | 147 |  | NEO |
|  |  | AINVAVHVFR | KVLDAVRGSP | 45 | 147 |  | MATURE |
|  |  | LLSPYSYSTTAVVTNPKE | SGPRRYTIAA | 130 | 147 |  | MATURE |
|  |  | LSPYSYSTTAVVTNPKE | GPRRYTIAAL | 131 | 147 |  | MATURE |
|  |  | NVAVHVFR | LDAVRGSPAI | 47 | 147 |  | MATURE |
| P02768 | Albumin | VLIAFAQYLQQCPFEDHVKLVNEVTEFAK | DLGEENFKAL | 46 | 609 |  | NEO |
|  |  | AEDYLSVVLNQLCVLHEK | KHPEAKRMPC | 472 | 609 |  | NEO |
|  |  | ADDRADLAK | ECCHGDLLEC | 278 | 609 |  | NEO |
|  |  | AFHDNEETFLKK | VRPEVDVMCT | 150 | 609 |  | NEO |
|  |  | ALEVDETYVPK | SLVNRRPCFS | 514 | 609 |  | NEO |
|  |  | ALEVDETYVPKEFNAETFTFHADICTLSEKER | SLVNRRPCFS | 514 | 609 |  | NEO |
|  |  | APELLFFAKR | EIARRHPYFY | 175 | 609 | MEP1B;BACE2 | NEO |
|  |  | AQYLQQCPFEDHVKLVNEVTEFAKTCVADESAENCDK | NFKALVLIAF | 52 | 609 |  | NEO |
|  |  | CDKSLHTLFGDKLCTVATLR | TCVADESAEN | 86 | 609 |  | NEO |
|  |  | CHGDLLECADDR | TDLTKVHTEC | 270 | 609 |  | NEO |
|  |  | CQAADKAACLLPK | KRYKAAFTEC | 193 | 609 |  | NEO |
|  |  | CTVATLR | SLHTLFGDKL | 99 | 609 |  | NEO |
|  |  | DNPNLPR | RNECFLQHKD | 132 | 609 |  | NEO |
|  |  | DNPNLPRLVRPEVDVMCTAFHDNEETFLK | RNECFLQHKD | 132 | 609 |  | NEO |
|  |  | ELRDEGKASSAK | KAACLLPKLD | 208 | 609 |  | NEO |
|  |  | EVDETYVPK | VNRRPCFSAL | 516 | 609 |  | NEO |
|  |  | FHADICTLSEKER | PKEFNAETFT | 533 | 609 |  | NEO |
|  |  | FHDNEETFLK | RPEVDVMCTA | 151 | 609 |  | NEO |
|  |  | FSALEVDETYVPKEFNAETFTFHADICTLSEK | TESLVNRRPC | 512 | 609 |  | NEO |
|  |  | GMFLYEYAR | NYAEAKDVFL | 352 | 609 |  | NEO |
|  |  | IAEVENDEMPADLPSLAADFVESK | EKPLLEKSHC | 314 | 609 |  | NEO |
|  |  | ICTLSEKER | NAETFTFHAD | 537 | 609 |  | NEO |
|  |  | KLCTVATLR | DKSLHTLFGD | 97 | 609 |  | NEO |
|  |  | KLVTDLTKVHTECCHGDLLECADDR | FPKAEFAEVS | 257 | 609 |  | NEO |
|  |  | KPLLEKSHCIAEVENDEMPADLPSLAADFVESK | ISSKLKECCE | 305 | 609 |  | NEO |
|  |  | LHTLFGDK | DESAENCDKS | 90 | 609 |  | NEO |
|  |  | LQQCPFEDHVKLVNEVTEFAK | ALVLIAFAQY | 55 | 609 |  | NEO |
|  |  | LRDEGKASSAK | AACLLPKLDE | 209 | 609 |  | NEO |
|  |  | LRETYGEMADCCAK | FGDKLCTVAT | 104 | 609 |  | NEO |
|  |  | MDDFAAFVEK | KATKEQLKAV | 572 | 609 |  | NEO |
|  |  | NDEMPADLPSLAADFVESKDVCKNYAEAK | EKSHCIAEVE | 319 | 609 |  | NEO |
|  |  | NEETFLKK | VDVMCTAFHD | 154 | 609 |  | NEO |
|  |  | QLCVLHEKTPVSDR | AEDYLSVVLN | 483 | 609 |  | NEO |
|  |  | QQCPFEDHVK | LVLIAFAQYL | 56 | 609 |  | NEO |
|  |  | RRPCFSALEVDETYVPK | TKCCTESLVN | 508 | 609 |  | NEO |
|  |  | SALEVDETYVPK | ESLVNRRPCF | 513 | 609 |  | NEO |
|  |  | SVVLNQLCVLHEKTPVSDR | KRMPCAEDYL | 478 | 609 |  | NEO |
|  |  | TAFHDNEETFLK | LVRPEVDVMC | 149 | 609 |  | NEO |
|  |  | TALVELVK | SEKERQIKKQ | 551 | 609 |  | NEO |
|  |  | TDLTKVHTECCHGDLLECADDRADLAK | AEFAEVSKLV | 260 | 609 |  | NEO |
|  |  | TKVHTECCHGDLLECADDRADLAK | AEVSKLVTDL | 263 | 609 |  | NEO |
|  |  | TLFGDKLCTVATLRETYGEMADCCAK | SAENCDKSLH | 92 | 609 |  | NEO |
|  |  | VADESAENCDKSLHTLFGDKLCTVATLR | NEVTEFAKTC | 78 | 609 |  | NEO |
|  |  | VENDEMPADLPSLAADFVESKDVCKNYAEAK | LLEKSHCIAE | 317 | 609 |  | NEO |
|  |  | VLNQLCVLHEKTPVSDR | MPCAEDYLSV | 480 | 609 |  | NEO |
|  |  | VMCTAFHDNEETFLKK | LPRLVRPEVD | 146 | 609 |  | NEO |
|  |  | VRPEVDVMCTAFHDNEETFLKK | KDDNPNLPRL | 140 | 609 |  | NEO |
|  |  | VRPEVDVMCTAFHDNEETFLKKYLYEIAR | KDDNPNLPRL | 140 | 609 |  | NEO |
|  |  | VSTPTLVEVSR | LVRYTKKVPQ | 442 | 609 |  | NEO |
|  |  | VVLNQLCVLHEKTPVSDRVTK | RMPCAEDYLS | 479 | 609 |  | NEO |
| P02774-3 | Isoform 3 of Vitamin D-binding protein | KVLEPTLK | RTHLPEVFLS | 382 | 493 |  | NEO |
| P02787 | Serotransferrin | AGWNIPMGLLYNKINHCR | KSCHTAVGRT | 477 | 698 |  | NEO |
|  |  | ARSMGGKEDLIWELLNQAQEHFGK | LAQVPSHTVV | 272 | 698 |  | NEO |
|  |  | CLDGTRKPVEEYANCHLAR | NLNEKDYELL | 582 | 698 |  | NEO |
|  |  | ETTEDCIAKIMNGEADAMSLDGGFVYIAGK | SVGKIECVSA | 391 | 698 |  | NEO |
|  |  | LAPNNLKPVVAEFYGSKEDPQTFYYAVAVVK | LDAGLVYDAY | 91 | 698 |  | NEO |
|  |  | LGYEYVTAIR | VPPRMDAKMY | 334 | 698 |  | NEO |
|  |  | MKSVIPSDGPSVACVK | ATKCQSFRDH | 45 | 698 |  | NEO |
|  |  | NHAVVTRK | YANCHLARAP | 603 | 698 | LGMN | NEO |
|  |  | NLCEPNNKEGYYGYTGAFR | LCKLCMGSGL | 523 | 698 |  | NEO |
|  |  | PNHAVVTRK | EYANCHLARA | 602 | 698 | LGMN | NEO |
|  |  | RFDEFFSEGCAPGSKK | GLLYNKINHC | 494 | 698 |  | NEO |
|  |  | SLLEACTFR | VGNLRKCSTS | 688 | 698 |  | NEO |
|  |  | VEKGDVAFVK | YGYTGAFRCL | 544 | 698 |  | NEO |
|  |  | VKHQTVPQNTGGKNPDPWAK | CLVEKGDVAF | 552 | 698 |  | NEO |
| P02790 | Beta-1B-glycoprotein | GPGLYLIHGPNLYCYSDVEKLNAAK | SLGPNSCSAN | 422 | 462 |  | NEO |
|  |  | GYTLVSGYPK | TQVYVFLTKG | 334 | 462 |  | NEO |
|  |  | PLPPTSAHGNVAEGETKPDPDVTER | SLCWSLAIAT | 25 | 462 |  | MATURE |
|  |  | SANGPGLYLIHGPNLYCYSDVEKLNAAK | MEKSLGPNSC | 419 | 462 |  | NEO |
| P04004 | Vitronectin | DWLVPATCEPIQSVFFFSGDKYYR | GANNYDDYRM | 423 | 478 |  | NEO |
| P04196 | Histidine-proline-rich glycoprotein Short=HPRG | VSPTDCSAVEPEAEK | LLITLQYSCA | 19 | 525 |  | MATURE |
| P04264 | 67 kDa cytokeratin | FASFIDKVR | REQIKSLNNQ | 191 | 644 |  | NEO |
| P04433 | Ig kappa chain V-III region VG | IVLTQSPATLSLSPGERATLSCR | LLWLPDTTGE | 22 | 115 |  | MATURE |
| P05060 | Secretogranin-1 | MPVDNRNHNEGMVTR | GAVGLAAVNS | 21 | 677 |  | MATURE |
| P06396 | AGEL | PFAQGALKSEDCFILDHGKDGK | MSVSLVADEN | 320 | 782 |  | NEO |
| P06727 | Apolipoprotein A4 | RAEVSADQVATVMWDYFSQLSNNAK | TLALVAVAGA | 19 | 396 |  | NEO |
| P07333 | CSF-1 receptor Short=CSF-1-R Short=CSF-1R Short=M-CSF-R EC=2.7.10.1 | IPVIEPSVPELVVKPGATVTLR | LVATAWHGQG | 20 | 972 |  | MATURE |
| P07711 | Major excreted protein Short=MEP | TLTFDHSLEAQWTKWK | AAFCLGIASA | 18 | 333 | CATL1 | MATURE |
| P07998 | Ribonuclease pancreatic | EGSPYVPVHFDASVEDST | PKERHIIVAC | 139 | 156 |  | NEO |
| P08253 | Matrix metalloproteinase-2 Short=MMP-2 | APSPIIKFPGDVAPK | LGCLLSHAAA | 30 | 660 |  | MATURE |
| P08294 | Extracellular superoxide dismutase [Cu-Zn] | CVVGVCGPGLWER | NGNAGRRLAC | 208 | 240 |  | NEO |
|  |  | WTGEDSAEPNSDSAEWIRDMYAK | LLLAAGASDA | 19 | 240 |  | MATURE |
| P0DJI8 | Serum amyloid A-1 protein | SFLGEAFDGAR | VLGVSSRSFF | 23 | 122 | CATL1;CATB;MMP3 | MATURE |
| P10451-4 | Isoform D of Osteopontin | IPVKQADSGSSEEK | CFCLLGITCA | 17 | 292 |  | NEO |
| P10619-2 | Isoform 2 of Lysosomal protective protein | APDQDEIQRLPGLAKQPSFR | LVSWASRGEA | 29 | 463 |  | NEO |
| P10745 | Retinol-binding protein 3 | ADNYASAELGAK | TVLQTAGKLV | 949 | 1247 |  | MATURE |
|  |  | ASVLTAGVQSSLNDPR | ILSISDPQTL | 71 | 1247 |  | MATURE |
|  |  | GSAAEAFAHTMQDLQR | DLYILMSHTS | 849 | 1247 |  | MATURE |
|  |  | SLSEEELLAWLQR | EPPPQVPALT | 107 | 1247 |  | MATURE |
| P10909-2 | Isoform 2 of Clusterin | ASHTSDSDVPSGVTEVVVK | DQYYLRVTTV | 442 | 501 |  | NEO |
|  |  | IHEAQQAMDIHFHSPAFQHPPTEFIR | HAMFQPFLEM | 303 | 501 |  | NEO |
|  |  | MALWEECKPCLK | ELPGVCNETM | 159 | 501 |  | NEO |
|  |  | MDIHFHSPAFQHPPTEFIREGDDDR | LEMIHEAQQA | 310 | 501 | MMP3;MMP12 | NEO |
|  |  | NFHAMFQPFLEMIHEAQQAMDIHFHSPAFQHPPTEFIR | LMPFSPYEPL | 291 | 501 |  | NEO |
|  |  | PITVTVPVEVSR | EVVVKLFDSD | 466 | 501 |  | NEO |
|  |  | SSIIDELFQDR | DVMQDHFSRA | 236 | 501 |  | NEO |
|  |  | THMLDVMQDHFSR | DSLLENDRQQ | 222 | 501 |  | NEO |
| P20396 | Pro-thyrotropin-releasing hormone | YGQAGLLLGLLDDLSR | LGGPCGPQGA | 203 | 242 |  | NEO |
| P22692 | IBP4_HUMAN | DEAIHCPPCSEEK | LAAGPGPSLG | 22 | 258 | KLK5;CAN1 | MATURE |
|  |  | DEAIHCPPCSEEKLAR | LAAGPGPSLG | 22 | 258 | KLK5;CAN1 | MATURE |
|  |  | ARCPGCGQGVQAGCPGGCVEEEDGGSPAEGCAEAEGCLRR | LLAASPGGAL | 27 | 240 |  | NEO |
| P31025 | Lipocalin-1 | HHLLASDEEIQDVSGTWYLK | SLGLIAALQA | 19 | 176 |  | MATURE |
| P32119 | Natural killer cell-enhancing factor B Short=NKEF-B | LFIIDGKGVLRQITVNDLPVGR | KTDEGIAYRG | 129 | 198 |  | NEO |
| P35613-2 | BASI-Isoform2_HUMAN | AAGTVFTTVEDLGSK | ALLGTHGASG | 22 | 269 |  | NEO |
| P36222 | 39 kDa synovial protein | YKLVCYYTSWSQYR | VLVLLQCCSA | 22 | 383 |  | MATURE |
| P36955 | Pigment epithelium-derived factor | AHFKGQWVTKFDSR | DEISILLLGV | 211 | 418 |  | NEO |
|  |  | GKPIKLTQVEHR | FDSPDFSKIT | 348 | 418 |  | NEO |
|  |  | HLTFPLDYHLNQPFIFVLR | TTPSPGLQPA | 381 | 418 |  | NEO |
|  |  | QNPASPPEEGSPDPDSTGALVEEEDPFFKVPVNK | IGALLGHSSC | 20 | 418 |  | MATURE |
| P41222 | Prostaglandin-H2 D-isomerase | CKAQGFTEDTIVFLPQTDK | AELKEKFTAF | 167 | 190 |  | NEO |
|  |  | FQQDKFLGR | PEAQVSVQPN | 34 | 190 |  | MATURE |
|  |  | VSVQPNFQQDKFLGR | GDLQAAPEAQ | 28 | 190 |  | MATURE |
| P43251-2 | Isoform 2 of Biotinidase | AHTGEESVADHHEAEYYVAAVYEHPSILSLNPLALISR | LCGCYVVALG | 44 | 545 |  | NEO |
| P47972 | Neuronal pentraxin II Short=NP-II | DLPRDPGHVVEQLSR | AGATGKDTMG | 115 | 431 |  | MATURE |
| P61769 | Beta-2-microglobulin form pI 5.3 Flags: Precursor | SRHPAENGKSNFLNCYVSGFHPSDIEVDLLK | IQRTPKIQVY | 31 | 119 |  | NEO |
| P61812-2 | Isoform B of Transforming growth factor beta-2 | LSTCSTLDMDQFMR | ILHLVTVALS | 21 | 442 |  | NEO |
| P63211 | Guanine nucleotide-binding protein G(T) subunit gamma-T1 | PEDKNPFKELK | SGEDPLVKGI | 58 | 74 |  | NEO |
| P81172 | Liver-expressed antimicrobial peptide 1 Short=LEAP-1 | SVFPQQTGQLAELQPQDR | LLLLASLTSG | 25 | 84 |  | MATURE |
| P98160 | Basement membrane-specific heparan sulfate proteoglycan core protein | DAPGQYGAYFHDDGFLAFPGHVFSR | DWHLEGSGGN | 4197 | 4391 | BMP1 | NEO |
| Q14767 | LTBP2_HUMAN | FEGLQAEECGILNGCENGR | VASHPEPPAG | 1729 | 1821 |  | NEO |
|  |  | FREQDAPVAGLQPVER | AAAAAKVYSL | 72 | 1821 |  | MATURE |
|  |  | LQAEECGILNGCENGR | HPEPPAGFEG | 1732 | 1821 |  | NEO |
| Q15149-2 | PLEC-Isoform2_HUMAN | MNKVYRQTNLENLDQAFSVAER | IHRHKPLLID | 234 | 4574 |  | NEO |
| Q15846 | Retinal-specific clusterin-like protein | APTWKDKTAISENLK | CLLWLKDSHC | 21 | 466 |  | MATURE |
| Q6MZW2 | Follistatin-related protein 4 | WMDPGTSRGPDVGVGESQAEEPR | LGASLPAALG | 23 | 842 |  | MATURE |
| Q86VZ4-2 | Isoform 2 of Low-density lipoprotein receptor-related protein 11 | ALPPAAPLSELHAQLSGVEQLLEEFRR | LCLWLPSGRA | 38 | 237 |  | NEO |
| Q8WXD2 | Secretogranin-3 | FPKPGGSQDKSLHNR | ILVLVLPIQA | 20 | 468 |  | MATURE |
| Q96JP9 | Photoreceptor cadherin Short=prCAD | KIDITDAETLSR | SPSFSTTALL | 673 | 859 |  | NEO |
| Q96PD5 | N-acetylmuramoyl-L-alanine amidase | AFLNGALDGVILGDYLSR | LDPKASLLTM | 271 | 576 |  | NEO |
|  |  | LPLLMDSVIQALAELEQKVPAAK | LLWSDPGTAS | 23 | 576 |  | MATURE |
| Q9BQT9-2 | Isoform 2 of Calsyntenin-3 | VLSSQQFLHR | SMNRVAHPSH | 825 | 968 | ADA10;ADA17 | NEO |
| Q9BZV3 | Interphotoreceptor matrix proteoglycan of 200 kDa Short=IPM 200 | YLSIEEIQEPK | GDFPSLTAQT | 32 | 1241 |  | MATURE |
| Q9P2V4 | Retina-specific protein PAL | FCPSQCSCSLHIMGDGSK | ALAWPPQARG | 22 | 623 |  | MATURE |
| Q9UBM4 | Opticin | FCDPEEHKHTR | NLIETMQRDV | 288 | 332 |  | NEO |
| Q9UM22-2 | EPDR1-Isoform2_HUMAN | APRPCQAPQQWEGR | CGLCSLGAVG | 38 | 90 |  | NEO |

**Supplementary Table 6.** C-termini identified (FDR≤0.01) in VH (based on identification in ≥50% subjects per group). Table reports the UniProt accession number, the protein description, the aminoacidic sequence of the peptide identified, the P1’ to P10’ residues, the P1 position, protein length, condition, previous evidence of proteolysis by a protease (when available) and peptide features (mature vs neo).

| **Accession** | **Description** | **Peptide Sequence** | **P1' to P10'** | **P1 Position** | **Protein length (aa)** | **Peptide condition** | **Proteases** | **C-terminal Features** |
| --- | --- | --- | --- | --- | --- | --- | --- | --- |
| B9A064 | Immunoglobulin lambda-like polypeptide 5 | SYSCQVTHEGSTVEKTVAPTEC | S | 213 | 214 | Shared |  | NEO |
| O15240 | Neuroendocrine regulatory peptide-1 | LADLASDLLLQYLLQGGARQ | RGLGGRGLQE | 343 | 615 | RRD |  | NEO |
| O75326 | Semaphorin-7A | EAQHWQLLPEDGIMAEH | LLGHACALAA | 639 | 666 | Shared |  | NEO |
| O94985-2 | Isoform 2 of Calsyntenin-1 | SFVDLSGHNLANPHP | FAVVPSTATV | 842 | 971 | Shared |  | NEO |
|  |  | SFVDLSGHNLANPHPF | AVVPSTATVV | 843 | 971 | Shared |  | NEO |
|  |  | SFVDLSGHNLANPHPFAVVPS | TATVVIVVCV | 848 | 971 | Shared |  | NEO |
|  |  | SFVDLSGHNLANPHPFAVVPSTA | TVVIVVCVSF | 850 | 971 | Shared |  | NEO |
|  |  | SFVDLSGHNLANPHPFAVVPSTAT | VVIVVCVSFL | 851 | 971 | Shared |  | NEO |
|  |  | SFVDLSGHNLANPHPFAVVPSTATVV | IVVCVSFLVF | 853 | 971 | Shared |  | NEO |
|  |  | YISNEFKVEVNVIHTANPMEHANH | MAAQPQFVHP | 814 | 971 | Shared |  | NEO |
| P00441 | Superoxide dismutase [Cu-Zn] | AVCVLKGDGPVQGIINFEQKES | NGPVKVWGSI | 26 | 154 | Shared |  | NEO |
| P00738 | Zonulin | AVGDKLPECEAVCGKPKNPANPVQ | RILGGHLDAK | 160 | 406 | Shared | C1RL | NEO |
| P01008 | Antithrombin-III | SLNPNRVTF | KANRPFLVFI | 434 | 464 | RRD |  | NEO |
| P01023 | C3 and PZP-like alpha-2-macroglobulin domain-containing protein 5 | ASVSVLGDILGSAMQNTQNLLQMPYGCGE | QNMVLFAPNI | 974 | 1474 | Shared |  | NEO |
| P01024 | C3 and PZP-like alpha-2-macroglobulin domain-containing protein 1 | LKHLIVTPSGCGE | QNMIGMTPTV | 1012 | 1663 | RRD |  | NEO |
|  |  | ADIGCTPGSGKDYAGVFSDAGLTFTSSSGQQTAQRAELQCPQPAA | RRRRSVQLTE | 667 | 1663 | Shared |  | NEO |
|  |  | HLIVTPSGCGE | QNMIGMTPTV | 1012 | 1663 | Shared |  | NEO |
|  |  | ILLQGTPVAQMTEDAVDAERLKHLIVTPSGCGE | QNMIGMTPTV | 1012 | 1663 | Shared |  | NEO |
|  |  | TLDPERLG | REGVQKEDIP | 953 | 1663 | Shared |  | NEO |
|  |  | AELQCPQPAA | RRRRSVQLTE | 667 | 1663 | CTRL |  | NEO |
| P01034 | Cystatin-3 | KAFCSFQIY | AVPWQGTMTL | 128 | 146 | Shared | CATD | NEO |
| P01344 | Somatomedin-A | SCDLALLETYCATPAKSE | RDVSTPPTVL | 91 | 180 | Shared | IDE;PCSK5;PCSK7;NEC2;PCSK4;FURIN;PCSK6 | NEO |
| P01857-1 | Isoform 1 of Immunoglobulin heavy constant gamma 1 | SRWQQGNVFSC | SVMHEALHNH | 309 | 330 | RRD |  | NEO |
|  |  | WQQGNVFSCSVMHEALHNHYTQKSLSLSPG | K | 329 | 330 | RRD |  | NEO |
|  |  | SRWQQGNVFSCSVMHEALHNHYTQKSLSLSPG | K | 329 | 330 | Shared |  | NEO |
|  |  | TPEVTCVVVDVSHED | PEVKFNWYVD | 153 | 330 | Shared |  | NEO |
| P01861-1 | Immunoglobulin heavy constant gamma 4 | WQEGNVFSCSVMHEALHNHYTQKSLSLSLG | K | 326 | 327 | Shared |  | NEO |
| P02489 | Heat shock protein beta-4 | AIPVSREEKPTSAPS | S | 172 | 173 | CTRL |  | NEO |
|  |  | FVIFLDVKHF | SPEDLTVKVQ | 80 | 173 | CTRL |  | NEO |
|  |  | HFSPEDLTVKV | QDDFVEIHGK | 89 | 173 | CTRL |  | NEO |
|  |  | HFSPEDLTVKVQD | DFVEIHGKHN | 91 | 173 | CTRL |  | NEO |
|  |  | SDRDKFVIFL | DVKHFSPEDL | 75 | 173 | CTRL |  | NEO |
|  |  | TLGPFYPSRL | FDQFFGEGLF | 22 | 173 | CTRL |  | NEO |
|  |  | TLGPFYPSRLFD | QFFGEGLFEY | 24 | 173 | CTRL |  | NEO |
|  |  | TLGPFYPSRLFDQ | FFGEGLFEYD | 25 | 173 | CTRL |  | NEO |
|  |  | TLGPFYPSRLFDQFF | GEGLFEYDLL | 27 | 173 | CTRL |  | NEO |
|  |  | TLGPFYPSRLFDQFFG | EGLFEYDLLP | 28 | 173 | CTRL |  | NEO |
|  |  | TLGPFYPSRLFDQFFGEG | LFEYDLLPFL | 30 | 173 | CTRL |  | NEO |
|  |  | TVLDSGISEVRS | DRDKFVIFLD | 66 | 173 | CTRL |  | NEO |
|  |  | TVLDSGISEVRSD | RDKFVIFLDV | 67 | 173 | CTRL |  | NEO |
|  |  | YRLPSNVDQSALSCSLSADGMLTFCGPKIQ | TGLDATHAER | 147 | 173 | CTRL |  | NEO |
| P02511 | Alpha(B)-crystallin | DRFSVNLDVKHF | SPEELKVKVL | 84 | 175 | CTRL |  | NEO |
|  |  | KYRIPADVD | PLTITSSLSS | 129 | 175 | CTRL | MMP9 | NEO |
|  |  | KYRIPADVDPLTI | TSSLSSDGVL | 133 | 175 | CTRL | MMP9 | NEO |
|  |  | KYRIPADVDPLTIT | SSLSSDGVLT | 134 | 175 | CTRL | MMP9 | NEO |
|  |  | KYRIPADVDPLTITS | SLSSDGVLTV | 135 | 175 | CTRL | MMP9 | NEO |
|  |  | KYRIPADVDPLTITSSL | SSDGVLTVNG | 137 | 175 | CTRL | MMP9 | NEO |
|  |  | KYRIPADVDPLTITSSLS | SDGVLTVNGP | 138 | 175 | CTRL | MMP9 | NEO |
|  |  | LFDQFFGEH | LLESDLFPTS | 31 | 175 | CTRL | MMP9 | NEO |
|  |  | LFDQFFGEHLL | ESDLFPTSTS | 33 | 175 | CTRL | MMP9 | NEO |
|  |  | LFDQFFGEHLLESDLFPT | STSLSPFYLR | 40 | 175 | CTRL | MMP9 | NEO |
|  |  | LFDQFFGEHLLESDLFPTS | TSLSPFYLRP | 41 | 175 | CTRL | MMP9 | NEO |
|  |  | LFDQFFGEHLLESDLFPTST | SLSPFYLRPP | 42 | 175 | CTRL | MMP9 | NEO |
|  |  | LFDQFFGEHLLESDLFPTSTSL | SPFYLRPPSF | 44 | 175 | CTRL | MMP9 | NEO |
|  |  | RPFFPFH | SPSRLFDQFF | 18 | 175 | CTRL | MMP9 | NEO |
|  |  | RPFFPFHSPSRLFD | QFFGEHLLES | 25 | 175 | CTRL | MMP9 | NEO |
|  |  | RPFFPFHSPSRLFDQFFGEHLLE | SDLFPTSTSL | 34 | 175 | CTRL | MMP9 | NEO |
|  |  | TIPITREEKPAVTAA | PKK | 172 | 175 | CTRL | MMP9 | NEO |
|  |  | VLGDVIEVHGKH | EERQDEHGFI | 104 | 175 | CTRL | MMP9 | NEO |
|  |  | YRIPADVD | PLTITSSLSS | 129 | 175 | CTRL | MMP9 | NEO |
|  |  | YRIPADVDPLTITSSL | SSDGVLTVNG | 137 | 175 | CTRL | MMP9 | NEO |
| P02647 | Apolipoprotein A1 | LSPLGEEMRD | RARAHVDALR | 174 | 267 | RRD |  | NEO |
|  |  | VSFLSALEEYTKKLNT | Q | 266 | 267 | Shared |  | NEO |
| P02649 | Apolipoprotein E | LGPLVEQGRV | RAATVGSLAG | 208 | 317 | RRD |  | NEO |
| P02652 | Apolipoprotein A2 | KAGTELVNFLSYFVELGTQPA | TQ | 98 | 100 | RRD |  | NEO |
|  |  | AGTELVNFLSYFVELGTQPA | TQ | 98 | 100 | Shared |  | NEO |
|  |  | AGTELVNFLSYFVELGTQPAT | Q | 99 | 100 | Shared |  | NEO |
|  |  | KAGTELVNFLSYFVELGTQPAT | Q | 99 | 100 | Shared |  | NEO |
| P02748 | Complement component C9a | QKISEGLPALEFPNE | K | 558 | 559 | Shared |  | NEO |
| P02753 | Retinol-binding protein 4 | LIVHNGYCDGRSERN | LL | 199 | 201 | CTRL |  | NEO |
| P02760 | Alpha-1-microglobulin | VVAQGVGIPEDSIFTMADRGECVPGEQEPEPILIPRV | RRAVLPQEEE | 203 | 352 | Shared |  | NEO |
| P02765 | Alpha-2-Z-globulin | HTFMGVVSLGSPSGEVSHP | RKTRTVVQPS | 336 | 367 | Shared |  | NEO |
|  |  | HTFMGVVSLGSPSGEVSHPRKT | RTVVQPSVGA | 339 | 367 | Shared |  | NEO |
| P02766 | Transthyretin | ALGISPFHEHAEVVFTAN | DSGPRRYTIA | 118 | 147 | RRD |  | NEO |
|  |  | RYTIAALLSPYSY | STTAVVTNPK | 136 | 147 | RRD |  | NEO |
|  |  | RYTIAALLSPYSYSTTAVVTN | PKE | 144 | 147 | Shared |  | NEO |
| P02768 | Albumin | AAFTECCQAADKAAC | LLPKLDELRD | 201 | 609 | RRD |  | NEO |
|  |  | ALVLIAFAQ | YLQQCPFEDH | 53 | 609 | RRD |  | NEO |
|  |  | ALVLIAFAQYLQQCPFED | HVKLVNEVTE | 62 | 609 | RRD |  | NEO |
|  |  | ALVLIAFAQYLQQCPFEDHVKLVN | EVTEFAKTCV | 68 | 609 | RRD |  | NEO |
|  |  | CCTESLVNRRPCFSALEVDETYVPKEF | NAETFTFHAD | 526 | 609 | RRD |  | NEO |
|  |  | DVCKNYAEAKDVFLGM | FLYEYARRHP | 353 | 609 | RRD |  | NEO |
|  |  | DVCKNYAEAKDVFLGMFLYEY | ARRHPDYSVV | 358 | 609 | RRD |  | NEO |
|  |  | DVFLGMFLYEY | ARRHPDYSVV | 358 | 609 | RRD |  | NEO |
|  |  | RHPYFYAPEL | LFFAKRYKAA | 178 | 609 | RRD |  | NEO |
|  |  | RMPCAEDYLSVVLN | QLCVLHEKTP | 482 | 609 | RRD |  | NEO |
|  |  | RMPCAEDYLSVVLNQLCVLH | EKTPVSDRVT | 488 | 609 | RRD |  | NEO |
|  |  | SHCIAEVENDEMPADLPSLAADFVESKDVCKN | YAEAKDVFLG | 342 | 609 | RRD |  | NEO |
|  |  | SLHTLFGDKLCTVATLRE | TYGEMADCCA | 106 | 609 | RRD | CMA1 | NEO |
|  |  | TCVADESAENCDKSLHTLFGDKLCTVAT | LRETYGEMAD | 103 | 609 | RRD |  | NEO |
|  |  | TCVADESAENCDKSLHTLFGDKLCTVATL | RETYGEMADC | 104 | 609 | RRD |  | NEO |
|  |  | VFDEFKPLVEEPQNLIKQNCELFEQLGEYKFQNA | LLVRYTKKVP | 430 | 609 | RRD |  | NEO |
|  |  | ALVLIAFAQYLQQ | CPFEDHVKLV | 57 | 609 | Shared |  | NEO |
|  |  | AVMDDFAAFVEKC | CKADDKETCF | 582 | 609 | Shared |  | NEO |
|  |  | AVMDDFAAFVEKCC | KADDKETCFA | 583 | 609 | Shared |  | NEO |
|  |  | CCTESLVNRRPCFSAL | EVDETYVPKE | 515 | 609 | Shared |  | NEO |
|  |  | CCTESLVNRRPCFSALEVDETYVPKEFN | AETFTFHADI | 527 | 609 | Shared |  | NEO |
|  |  | CCTESLVNRRPCFSALEVDETYVPKEFNAETF | TFHADICTLS | 531 | 609 | Shared |  | NEO |
|  |  | CCTESLVNRRPCFSALEVDETYVPKEFNAETFTF | HADICTLSEK | 533 | 609 | Shared |  | NEO |
|  |  | DVCKNYAEAKDVFLGMF | LYEYARRHPD | 354 | 609 | Shared |  | NEO |
|  |  | DVCKNYAEAKDVFLGMFL | YEYARRHPDY | 355 | 609 | Shared |  | NEO |
|  |  | FKDLGEENFKAL | VLIAFAQYLQ | 46 | 609 | Shared |  | NEO |
|  |  | FQNALLVRY | TKKVPQVSTP | 435 | 609 | Shared |  | NEO |
|  |  | HPYFYAPELL | FFAKRYKAAF | 179 | 609 | Shared |  | NEO |
|  |  | HPYFYAPELLF | FAKRYKAAFT | 180 | 609 | Shared |  | NEO |
|  |  | HPYFYAPELLFF | AKRYKAAFTE | 181 | 609 | Shared |  | NEO |
|  |  | KLVAASQAAL | GL | 607 | 609 | Shared |  | NEO |
|  |  | KLVAASQAALG | L | 608 | 609 | Shared |  | NEO |
|  |  | LAKTYETTLEKC | CAAADPHECY | 384 | 609 | Shared |  | NEO |
|  |  | LAKTYETTLEKCC | AAADPHECYA | 385 | 609 | Shared |  | NEO |
|  |  | LAKTYETTLEKCCAAAD | PHECYAKVFD | 389 | 609 | Shared |  | NEO |
|  |  | LKECCEKPLLEKSH | CIAEVENDEM | 312 | 609 | Shared |  | NEO |
|  |  | LKECCEKPLLEKSHC | IAEVENDEMP | 313 | 609 | Shared |  | NEO |
|  |  | LKECCEKPLLEKSHCIAEVENDEMPAD | LPSLAADFVE | 325 | 609 | Shared |  | NEO |
|  |  | LKECCEKPLLEKSHCIAEVENDEMPADLPSLAADFV | ESKDVCKNYA | 334 | 609 | Shared |  | NEO |
|  |  | LVAASQAAL | GL | 607 | 609 | Shared |  | NEO |
|  |  | LVAASQAALG | L | 608 | 609 | Shared |  | NEO |
|  |  | LVRPEVDVM | CTAFHDNEET | 147 | 609 | Shared |  | NEO |
|  |  | LVRPEVDVMC | TAFHDNEETF | 148 | 609 | Shared |  | NEO |
|  |  | LVRPEVDVMCT | AFHDNEETFL | 149 | 609 | Shared |  | NEO |
|  |  | LVRPEVDVMCTAF | HDNEETFLKK | 151 | 609 | Shared |  | NEO |
|  |  | LVRPEVDVMCTAFH | DNEETFLKKY | 152 | 609 | Shared |  | NEO |
|  |  | LVRPEVDVMCTAFHD | NEETFLKKYL | 153 | 609 | Shared |  | NEO |
|  |  | LVRPEVDVMCTAFHDN | EETFLKKYLY | 154 | 609 | Shared |  | NEO |
|  |  | LVRPEVDVMCTAFHDNEETFLKKY | LYEIARRHPY | 162 | 609 | Shared |  | NEO |
|  |  | LVRPEVDVMCTAFHDNEETFLKKYLY | EIARRHPYFY | 164 | 609 | Shared |  | NEO |
|  |  | LVRPEVDVMCTAFHDNEETFLKKYLYEIA | RRHPYFYAPE | 167 | 609 | Shared | BACE2 | NEO |
|  |  | LVTDLTKVHTEC | CHGDLLECAD | 269 | 609 | Shared |  | NEO |
|  |  | MPCAEDYLSVVLNQL | CVLHEKTPVS | 484 | 609 | Shared |  | NEO |
|  |  | NYAEAKDVFLGMF | LYEYARRHPD | 354 | 609 | Shared |  | NEO |
|  |  | QNCELFEQLGEYKFQN | ALLVRYTKKV | 429 | 609 | Shared |  | NEO |
|  |  | QNCELFEQLGEYKFQNAL | LVRYTKKVPQ | 431 | 609 | Shared |  | NEO |
|  |  | QNCELFEQLGEYKFQNALLVRY | TKKVPQVSTP | 435 | 609 | Shared |  | NEO |
|  |  | RHPDYSVVL | LLRLAKTYET | 369 | 609 | Shared |  | NEO |
|  |  | RHPDYSVVLL | LRLAKTYETT | 370 | 609 | Shared |  | NEO |
|  |  | RHPYFYAPELL | FFAKRYKAAF | 179 | 609 | Shared |  | NEO |
|  |  | RHPYFYAPELLF | FAKRYKAAFT | 180 | 609 | Shared |  | NEO |
|  |  | RHPYFYAPELLFF | AKRYKAAFTE | 181 | 609 | Shared |  | NEO |
|  |  | RMPCAEDYLSVVLNQL | CVLHEKTPVS | 484 | 609 | Shared |  | NEO |
|  |  | RMPCAEDYLSVVLNQLCVLHEKTPVSD | RVTKCCTESL | 495 | 609 | Shared |  | NEO |
|  |  | RPCFSALEVDETYVPKEFNAETFTF | HADICTLSEK | 533 | 609 | Shared |  | NEO |
|  |  | SHCIAEVENDEMPADLPSLAAD | FVESKDVCKN | 332 | 609 | Shared |  | NEO |
|  |  | SLHTLFGDKLC | TVATLRETYG | 99 | 609 | Shared |  | NEO |
|  |  | SLHTLFGDKLCTVAT | LRETYGEMAD | 103 | 609 | Shared |  | NEO |
|  |  | TCVADESAENCDKSLHTLF | GDKLCTVATL | 94 | 609 | Shared |  | NEO |
|  |  | TCVADESAENCDKSLHTLFGDKLC | TVATLRETYG | 99 | 609 | Shared |  | NEO |
|  |  | TYETTLEKC | CAAADPHECY | 384 | 609 | Shared |  | NEO |
|  |  | TYETTLEKCC | AAADPHECYA | 385 | 609 | Shared |  | NEO |
|  |  | TYETTLEKCCAAAD | PHECYAKVFD | 389 | 609 | Shared |  | NEO |
|  |  | VFDEFKPLVEEPQNLIKQNCELFEQLGEYKFQN | ALLVRYTKKV | 429 | 609 | Shared |  | NEO |
|  |  | VFDEFKPLVEEPQNLIKQNCELFEQLGEYKFQNAL | LVRYTKKVPQ | 431 | 609 | Shared |  | NEO |
|  |  | YKAAFTEC | CQAADKAACL | 192 | 609 | Shared |  | NEO |
|  |  | YKAAFTECCQAADKAA | CLLPKLDELR | 200 | 609 | Shared |  | NEO |
|  |  | YKAAFTECCQAADKAAC | LLPKLDELRD | 201 | 609 | Shared |  | NEO |
|  |  | YLYEIARRHPY | FYAPELLFFA | 172 | 609 | Shared | BACE2;MEP1B | NEO |
|  |  | CCTESLVNRRPC | FSALEVDETY | 511 | 609 | CTRL |  | NEO |
|  |  | DVCKNYAEAKDVFLGMFLYE | YARRHPDYSV | 357 | 609 | CTRL |  | NEO |
|  |  | FKDLGEENFKALVL | IAFAQYLQQC | 48 | 609 | CTRL |  | NEO |
|  |  | NYAEAKDVFLGMFL | YEYARRHPDY | 355 | 609 | CTRL |  | NEO |
| P02787 | Serotransferrin | EGTCPEAPTDECKPVKWCAL | SHHERLKCDE | 366 | 698 | RRD |  | NEO |
|  |  | HSTIFENLAN | KADRDQYELL | 235 | 698 | RRD |  | NEO |
|  |  | KCSTSSLLEACTF | RRP | 695 | 698 | RRD |  | NEO |
|  |  | NLREGTCPEAPTDECKPVKWC | ALSHHERLKC | 364 | 698 | RRD |  | NEO |
|  |  | SAGWNIPIGLLY | CDLPEPRKPL | 155 | 698 | RRD |  | NEO |
|  |  | TAGWNIPMGLLYNKINH | CRFDEFFSEG | 492 | 698 | RRD |  | NEO |
|  |  | TAGWNIPMGLLYNKINHC | RFDEFFSEGC | 493 | 698 | RRD |  | NEO |
|  |  | AIAANEADAVTLDAGLVYDAYLAPNNLKPVVAEFYGSKED | PQTFYYAVAV | 109 | 698 | Shared |  | NEO |
|  |  | CLVEKGDVAF | VKHQTVPQNT | 551 | 698 | Shared |  | NEO |
|  |  | EGTCPEAPTDECKPVKWC | ALSHHERLKC | 364 | 698 | Shared |  | NEO |
|  |  | HQTVPQNTGGKNPD | PWAKNLNEKD | 567 | 698 | Shared |  | NEO |
|  |  | SAGWNIPIGLL | YCDLPEPRKP | 154 | 698 | Shared |  | NEO |
| P02790 | Beta-1B-glycoprotein | NFPSPVDAAFRQGHN | SVFLIKGDKV | 106 | 462 | Shared |  | NEO |
|  |  | WKNFPSPVDAAFRQGHN | SVFLIKGDKV | 106 | 462 | Shared |  | NEO |
|  |  | CSPHLVLSALTSDN | HGATYAFSGT | 270 | 462 | CTRL |  | NEO |
| P04004 | Vitronectin | IYISGMAPRPSLA | KKQRFRHRNR | 366 | 478 | Shared |  | NEO |
|  |  | IYISGMAPRPSLAKKQ | RFRHRNRKGY | 369 | 478 | Shared |  | NEO |
|  |  | SIAQYWLGCPAPGH | L | 477 | 478 | Shared |  | NEO |
| P05060 | Secretogranin-1 | AYFMSDTREE | KRFLGEGHHR | 437 | 677 | CTRL |  | NEO |
|  |  | FQDKQYSSHHTAE | KRKRLGELFN | 513 | 677 | CTRL |  | NEO |
| P05813 | Beta-crystallin A3, isoform A1, Delta4 form | GEYPRWDAW | SGSNAYHIER | 99 | 215 | CTRL |  | NEO |
|  |  | GEYPRWDAWSG | SNAYHIERLM | 101 | 215 | CTRL |  | NEO |
|  |  | GYQYILECDH | HGGDYKHWRE | 187 | 215 | CTRL |  | NEO |
|  |  | IQSGAWVCYQYPGYRGYQYILECDH | HGGDYKHWRE | 187 | 215 | CTRL |  | NEO |
|  |  | IQSGAWVCYQYPGYRGYQYILECDHHG | GDYKHWREWG | 189 | 215 | CTRL |  | NEO |
|  |  | LMSFRPIC | SANHKESKMT | 117 | 215 | CTRL |  | NEO |
| P07315 | Gamma-crystallin 2-1 | LYEREDHKGLMMEL | SEDCPSIQDR | 105 | 174 | CTRL |  | NEO |
|  |  | LYEREDHKGLMMELSED | CPSIQDRFHL | 108 | 174 | CTRL |  | NEO |
|  |  | RGEYPDYQQWMGL | SDSIRSCCLI | 72 | 174 | CTRL |  | NEO |
|  |  | RGEYPDYQQWMGLSD | SIRSCCLIPQ | 74 | 174 | CTRL |  | NEO |
|  |  | SLHVLEGCWVL | YELPNYRGRQ | 133 | 174 | CTRL |  | NEO |
| P07320 | Gamma-crystallin 4 | EDYRGQMIEFTED | CSCLQDRFRF | 108 | 174 | CTRL |  | NEO |
|  |  | LYEREDYRGQMIEFTED | CSCLQDRFRF | 108 | 174 | CTRL |  | NEO |
| P08493 | Cell growth-inhibiting gene 36 protein | YAMVYGYNAAYNRYF | RKRRGTK | 96 | 103 | RRD |  | NEO |
| P08603 | Complement factor H | TTCWDGKLEYPTCA | KR | 1229 | 1231 | Shared |  | NEO |
| P0C0L5 | Complement C4-B | DDPDAPLQPVTPLQLFEGRRN | RRRREAPKVV | 1449 | 1744 | Shared |  | NEO |
|  |  | VTASDPLDTLGSEGALSPGGVASLLRLPRGCGE | QTMIYLAPTL | 1012 | 1744 | Shared |  | NEO |
| P10451-4 | Isoform D of Osteopontin | GKDSYETSQLDDQSAETHSHKQS | RLYKRKANDE | 221 | 292 | Shared |  | NEO |
| P10745 | Retinol-binding protein 3 | AKEMLQHNQLRV | KRSPGLQDHL | 1237 | 1247 | RRD |  | NEO |
|  |  | TAVDLESLASQLTADLQEV | SGDHRLLVFH | 693 | 1247 | RRD |  | NEO |
|  |  | YGADKDVVVLT | SSQTRGVAED | 231 | 1247 | RRD |  | NEO |
|  |  | AKEMLQHNQL | RVKRSPGLQD | 1235 | 1247 | Shared |  | NEO |
|  |  | EMLQHNQLRV | KRSPGLQDHL | 1237 | 1247 | Shared |  | NEO |
|  |  | FDSFADASVLGVLAPYVLRQ | VWEPLQDTEH | 463 | 1247 | Shared |  | NEO |
|  |  | SVGASDGSSWEGVGVTPHVVVPAEEALARAKEMLQHNQL | RVKRSPGLQD | 1235 | 1247 | Shared |  | NEO |
|  |  | IGESDFFFTVPV | SRSLGPLGGG | 282 | 1247 | CTRL |  | NEO |
| P10909-2 | Isoform 2 of Clusterin | ASSIIDELFQDRFFTREPQD | TYHYLPFSLP | 254 | 501 | RRD |  | NEO |
|  |  | ASSIIDELFQDRFFTREPQDTYHYLPF | SLPHRRPHFF | 261 | 501 | RRD |  | NEO |
|  |  | ASSIIDELFQDRF | FTREPQDTYH | 247 | 501 | Shared |  | NEO |
|  |  | ASSIIDELFQDRFFTREPQDT | YHYLPFSLPH | 255 | 501 | Shared |  | NEO |
|  |  | EILSVDCSTNNPSQAKL | RRELDESLQV | 375 | 501 | Shared |  | NEO |
|  |  | FFTREPQDT | YHYLPFSLPH | 255 | 501 | Shared |  | NEO |
|  |  | FFTREPQDTYHYLPF | SLPHRRPHFF | 261 | 501 | Shared |  | NEO |
|  |  | LFDSDPITVTVPVEV | SRKNPKFMET | 475 | 501 | Shared |  | NEO |
|  |  | RPHFFFPKS | RIVRSLMPFS | 275 | 501 | Shared | ATS4 | NEO |
|  |  | VTTVASHTSDSDVPSGVTEV | VVKLFDSDPI | 457 | 501 | Shared |  | NEO |
|  |  | ASSIIDELFQDRFFTREPQDTYHYLPFSLPH | RRPHFFFPKS | 265 | 501 | CTRL | ATS4 | NEO |
|  |  | FFTREPQDTYHYLPFSLPHRRPHF | FFPKSRIVRS | 270 | 501 | CTRL |  | NEO |
|  |  | VTTVASHTSDSDVPSGVTEVVVKLFDSDPITVTVPVEV | SRKNPKFMET | 475 | 501 | CTRL |  | NEO |
| P13521 | Secretogranin-2 | TNEIVEEQYTPQSLATLESVFQELGKLTGPNNQ | KRERMDEEQK | 214 | 617 | Shared |  | NEO |
| P16870-2 | Isoform 2 of Carboxypeptidase E | AYSSFNPAMSDPNRPPCRKNDDDSSFVDGT | TNGGAWYSVP | 277 | 440 | CTRL |  | NEO |
| P19022 | Cadherin-2 | LLLLLLL | VLMFVVWMKR | 738 | 906 | Shared |  | NEO |
|  |  | QFSKHSGHLQRQ | KRDWVIPPIN | 157 | 906 | Shared | KLK4 | NEO |
| P22914 | Gamma-crystallin S | AVHLPSGGQYKIQIFEKGDF | SGQMYETTED | 104 | 178 | CTRL |  | NEO |
|  |  | AVHLPSGGQYKIQIFEKGDFSGQMYETTED | CPSIMEQFHM | 114 | 178 | CTRL |  | NEO |
|  |  | CNSIKVEGGTWAVYERPN | FAGYMYILPQ | 54 | 178 | CTRL |  | NEO |
|  |  | CNSIKVEGGTWAVYERPNFAGY | MYILPQGEYP | 58 | 178 | CTRL |  | NEO |
|  |  | CNSIKVEGGTWAVYERPNFAGYMY | ILPQGEYPEY | 60 | 178 | CTRL |  | NEO |
|  |  | EIHSCKVLEGVWIF | YELPNYRGRQ | 139 | 178 | CTRL |  | NEO |
|  |  | EYRKPIDWGAASPAVQ | SFRRIVE | 171 | 178 | CTRL |  | NEO |
|  |  | IQIFEKGDF | SGQMYETTED | 104 | 178 | CTRL |  | NEO |
|  |  | IQIFEKGDFSG | QMYETTEDCP | 106 | 178 | CTRL |  | NEO |
|  |  | IQIFEKGDFSGQMYETTED | CPSIMEQFHM | 114 | 178 | CTRL |  | NEO |
|  |  | KPIDWGAA | SPAVQSFRRI | 166 | 178 | CTRL |  | NEO |
|  |  | KPIDWGAASPAVQ | SFRRIVE | 171 | 178 | CTRL |  | NEO |
|  |  | VLEGVWIFYELPN | YRGRQYLLDK | 144 | 178 | CTRL |  | NEO |
| P36955 | Pigment epithelium-derived factor | AGFEWNEDGAGTTPSPGLQPAHLTFPLDYHLNQPF | IFVLRDTDTG | 394 | 418 | RRD |  | NEO |
| P41222 | Prostaglandin-H2 D-isomerase | TMLLQPAGSLGSYSYRSPHWGSTY | SVSVVETDYD | 116 | 190 | Shared |  | NEO |
| P43320 | Beta-crystallin Bp | GLQYLLEKG | DYKDSSDFGA | 169 | 205 | CTRL |  | NEO |
|  |  | GLQYLLEKGD | YKDSSDFGAP | 170 | 205 | CTRL |  | NEO |
|  |  | GLQYLLEKGDYKD | SSDFGAPHPQ | 173 | 205 | CTRL |  | NEO |
|  |  | GLQYLLEKGDYKDS | SDFGAPHPQV | 174 | 205 | CTRL |  | NEO |
|  |  | GLQYLLEKGDYKDSSDFGAPHPQVQ | SVRRIRDMQW | 185 | 205 | CTRL |  | NEO |
|  |  | IIIFEQENFQGHSHELNGPCPN | LKETGVEKAG | 40 | 205 | CTRL |  | NEO |
|  |  | KMEIIDDDVPSFHAH | GYQEKVSSVR | 135 | 205 | CTRL |  | NEO |
|  |  | RIRDMQWH | QRGAFHPSN | 196 | 205 | CTRL |  | NEO |
|  |  | RTDSLSSLRPIKVD | SQEHKIILYE | 103 | 205 | CTRL |  | NEO |
| P49908 | Selenoprotein P | CINQLLCKLPTDSELAPRS | UCCHCRHLIF | 299 | 381 | Shared |  | NEO |
| P53673 | Beta-A4 crystallin | EWGSHAPTFQVQ | SIRRIQQ | 189 | 196 | CTRL |  | NEO |
|  |  | GFQYVLECDHH | SGDYKHFREW | 169 | 196 | CTRL |  | NEO |
|  |  | GFQYVLECDHHSGDYKHFREWG | SHAPTFQVQS | 180 | 196 | CTRL |  | NEO |
|  |  | LTSFRPAA | CANHRDSRLT | 98 | 196 | CTRL |  | NEO |
|  |  | SLKVLSGAWVGFEHAG | FQGQQYILER | 61 | 196 | CTRL |  | NEO |
|  |  | VLSGAWVGFEHAGF | QGQQYILERG | 62 | 196 | CTRL |  | NEO |
| P53674 | Beta-B1 crystallin | GEMFILEKG | EYPRWNTWSS | 119 | 252 | CTRL |  | NEO |
|  |  | GYQYLLEPGDFRHWN | EWGAFQPQMQ | 217 | 252 | CTRL |  | NEO |
|  |  | LMSFRPIKMDA | QEHKISLFEG | 146 | 252 | CTRL |  | NEO |
|  |  | LMSFRPIKMDAQ | EHKISLFEGA | 147 | 252 | CTRL |  | NEO |
|  |  | LRDKQWHLE | GSFPVLATEP | 240 | 252 | CTRL |  | NEO |
|  |  | LRDKQWHLEG | SFPVLATEPP | 241 | 252 | CTRL |  | NEO |
|  |  | LRDKQWHLEGSFPVL | ATEPPK | 246 | 252 | CTRL |  | NEO |
|  |  | LRDKQWHLEGSFPVLA | TEPPK | 247 | 252 | CTRL |  | NEO |
|  |  | LRDKQWHLEGSFPVLATE | PPK | 249 | 252 | CTRL |  | NEO |
|  |  | QWHLEGSFPVLA | TEPPK | 247 | 252 | CTRL |  | NEO |
|  |  | RAEFSGECSNLADRG | FDRVRSIIVS | 87 | 252 | CTRL |  | NEO |
|  |  | RAEFSGECSNLADRGF | DRVRSIIVSA | 88 | 252 | CTRL |  | NEO |
|  |  | SDRLMSFRPIKMD | AQEHKISLFE | 145 | 252 | CTRL |  | NEO |
|  |  | SIIVSAGPWVAFEQ | SNFRGEMFIL | 106 | 252 | CTRL |  | NEO |
| P63261 | Gamma-actin | VAPEEHPVLLTEAPLN | PKANREKMTQ | 111 | 375 | CTRL |  | NEO |
| P68363 | Tubulin alpha-1B chain | EDMAALEKDYEEVGVDSVEGEGE | EEGEEY | 445 | 451 | RRD |  | NEO |
| P69905 | Hemoglobin subunit alpha | VGAHAGEYGAEALERM | FLSFPTTKTY | 33 | 142 | RRD | CATD | NEO |
| Q06481 | Amyloid beta precursor like protein 2 | NKVDENMVIDETL | DVKEMIFNAE | 659 | 763 | RRD |  | NEO |
|  |  | NKVDENMVIDET | LDVKEMIFNA | 658 | 763 | Shared |  | NEO |
| Q12934 | Lens fiber cell beaded-filament structural protein CP 115 | AAEPERPADEGWAG | ATSLAALQGL | 37 | 665 | CTRL |  | NEO |
|  |  | AAEPERPADEGWAGA | TSLAALQGLG | 38 | 665 | CTRL |  | NEO |
|  |  | AAEPERPADEGWAGAT | SLAALQGLGE | 39 | 665 | CTRL |  | NEO |
|  |  | IIEIEGNRLTSAFIETPIPLF | TQSHGVSLST | 332 | 665 | CTRL |  | NEO |
|  |  | IIEIEGNRLTSAFIETPIPLFTQ | SHGVSLSTGS | 334 | 665 | CTRL |  | NEO |
|  |  | IIEIEGNRLTSAFIETPIPLFTQSH | GVSLSTGSGG | 336 | 665 | CTRL |  | NEO |
|  |  | LTSAFIETPIPLF | TQSHGVSLST | 332 | 665 | CTRL |  | NEO |
|  |  | LTSAFIETPIPLFT | QSHGVSLSTG | 333 | 665 | CTRL |  | NEO |
| Q13515 | Phakinin | ALGISSVFLQGLRS | SGLATVPAPG | 90 | 415 | CTRL |  | NEO |
|  |  | DVASYHALLDREE | SG | 413 | 415 | CTRL |  | NEO |
|  |  | LLHKQLAG | CELEQMDAPI | 254 | 415 | CTRL |  | NEO |
|  |  | MDLESQIESLKEELGSL | SRNYEEDVKL | 237 | 415 | CTRL |  | NEO |
|  |  | RALGISSVFLQGLRS | SGLATVPAPG | 90 | 415 | CTRL |  | NEO |
|  |  | SSGLATVPAPGLERDHG | AVEDLGGCLV | 106 | 415 | CTRL |  | NEO |
|  |  | SSGLATVPAPGLERDHGAVED | LGGCLVEYMA | 110 | 415 | CTRL |  | NEO |
|  |  | SSGLATVPAPGLERDHGAVEDLGG | CLVEYMAKVH | 113 | 415 | CTRL |  | NEO |
| Q14515-2 | Isoform 2 of SPARC-like protein 1 | KLSENTDFLAPGVSSFTDSNQQESIT | KREENQEQPR | 33 | 539 | CTRL |  | NEO |
| Q15904 | V-type proton ATPase subunit S1 | EVLTGNDEVIGQVLSTLKSEDVPYTAALTAVRPSRVA | RDVAVVAGGL | 230 | 470 | Shared |  | NEO |
|  |  | SEDVPYTAALTAVRPSRVA | RDVAVVAGGL | 230 | 470 | CTRL |  | NEO |
| Q17R60 | Interphotoreceptor matrix proteoglycan 1 | KFQNQQNNKVIS | KRNSELLTVE | 775 | 797 | RRD |  | NEO |
| Q8IZJ3-2 | Isoform 2 of C3 and PZP-like alpha-2-macroglobulin domain-containing protein 8 | GNIVLSGQQPAHTTQQRS | KRAAPALEKP | 509 | 1815 | Shared |  | NEO |
| Q92823-2 | Neuronal cell adhesion molecule - Isoform 2 | LLLLLLLL | VCFIRRNKGG | 1186 | 1236 | Shared |  | NEO |
| Q96PD5 | N-acetylmuramoyl-L-alanine amidase | TDCPGDALFDLLRTWPHFTATVKPRPA | RSVSKRSRRE | 554 | 576 | Shared |  | NEO |
|  |  | TWPHFTATVKPRPA | RSVSKRSRRE | 554 | 576 | Shared |  | NEO |
| Q99969 | Retinoic acid receptor responder protein 2 | AGEDPHSFYFPGQFA | FSKALPRS | 155 | 163 | Shared | ELNE;CATG;PLMN;TRYB1 | NEO |
| Q9BSG5-2 | Isoform 2 of Retbindin | HCFNISISAVPRPRPGRRG | REAPSRRSRS | 230 | 261 | CTRL |  | NEO |
| Q9BZV3 | Interphotoreceptor matrix proteoglycan 2 | ETERQWLI | RRRRSILFPN | 77 | 1241 | CTRL |  | NEO |
| Q9HCQ7 | Neuropeptide NPSF | KIDDAELKQE | K | 195 | 196 | CTRL |  | NEO |
| Q9NP84 | Tumor necrosis factor receptor superfamily member 12A | LLWPILGGALS | LTFVLGLLSG | 87 | 129 | RRD |  | NEO |
| Q9UHG2 | Proprotein convertase subtilisin/kexin type 1 inhibitor | AADHDVGSELPPEGVLGALLRV | KRLETPAPQV | 242 | 260 | RRD |  | NEO |
| Q9UHL4 | Dipeptidyl peptidase II | KLEATIIGEWVKA | ARREQQPALR | 475 | 492 | CTRL |  | NEO |
| Q9Y287-2 | Isoform 2 of Integral membrane protein 2B | EASNCFAIRH | FENKFAVETL | 147 | 160 | Shared |  | NEO |
|  |  | HFENKFA | VETLICS | 153 | 160 | Shared |  | NEO |
|  |  | HFENKFAVET | LICS | 156 | 160 | CTRL |  | NEO |
| Q9Y5W5 | Wnt inhibitory factor 1 | RYEASLIHALRPAGAQ | LRQHTPSLKK | 355 | 379 | Shared |  | NEO |
|  |  | YEASLIHALRPAGAQ | LRQHTPSLKK | 355 | 379 | Shared |  | NEO |
| Q9Y6R7 | IgGFc-binding protein | CREGGEVSCEPSSCGPHETCRPSGGSLGCVAVGSTTCQASGD | PHYTTFDGRR | 2458 | 5405 | Shared |  | NEO |
