# Supplementary material for: Altered pattern of proteolysis in rhegmatogenous retinal detachment by mining of N-termini datasets from vitreous humor proteome
Source: Sci Rep. 2025 Oct 14;15:35848. doi: 10.1038/s41598-025-19857-z (PMC12521590; doi:10.1038/s41598-025-19857-z)
Supplement: Supplementary file 2 — Supplementary Material 2 [file 41598_2025_19857_MOESM2_ESM.docx]

**SUPPLEMENTARY FIGURES**

A


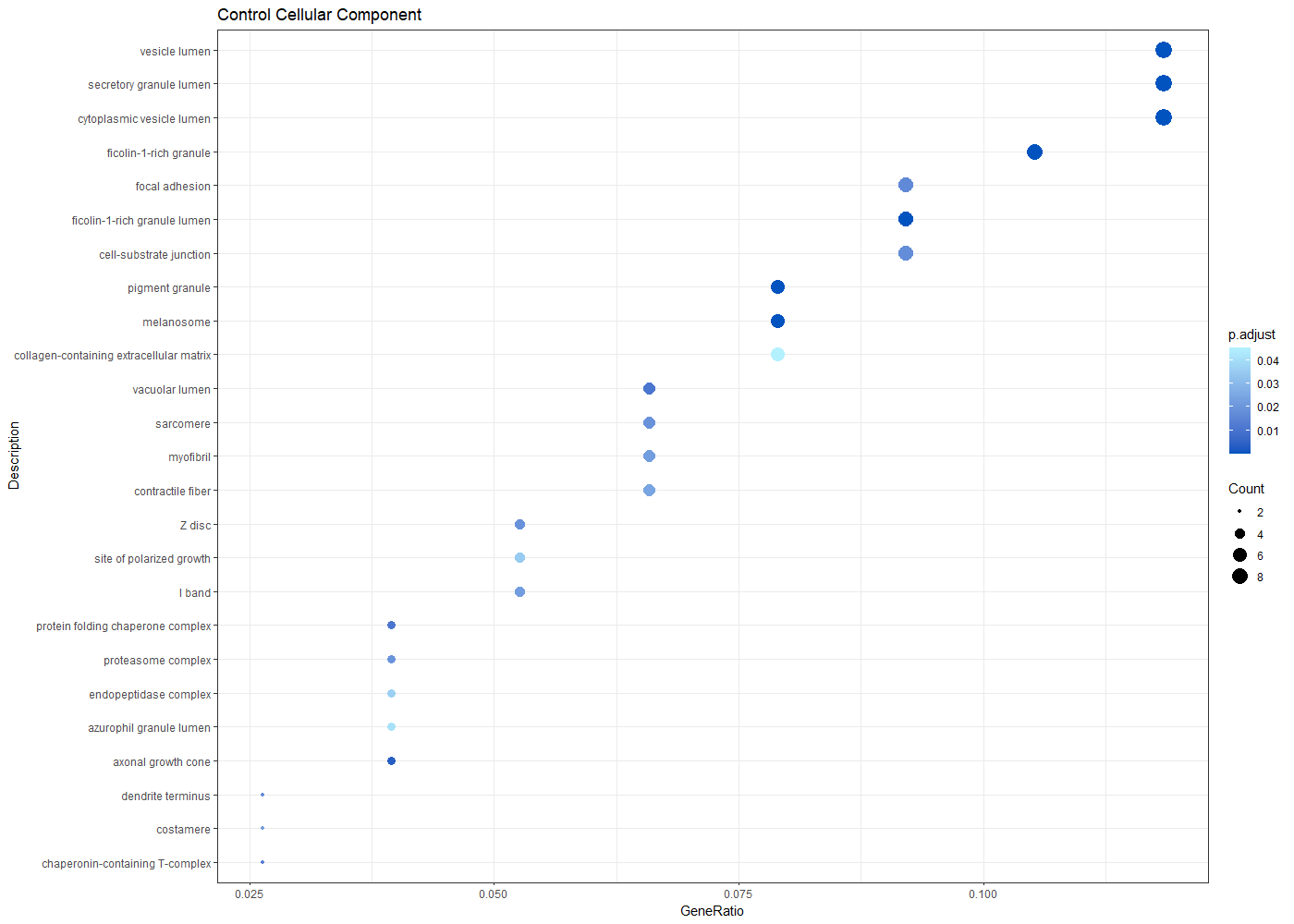


B


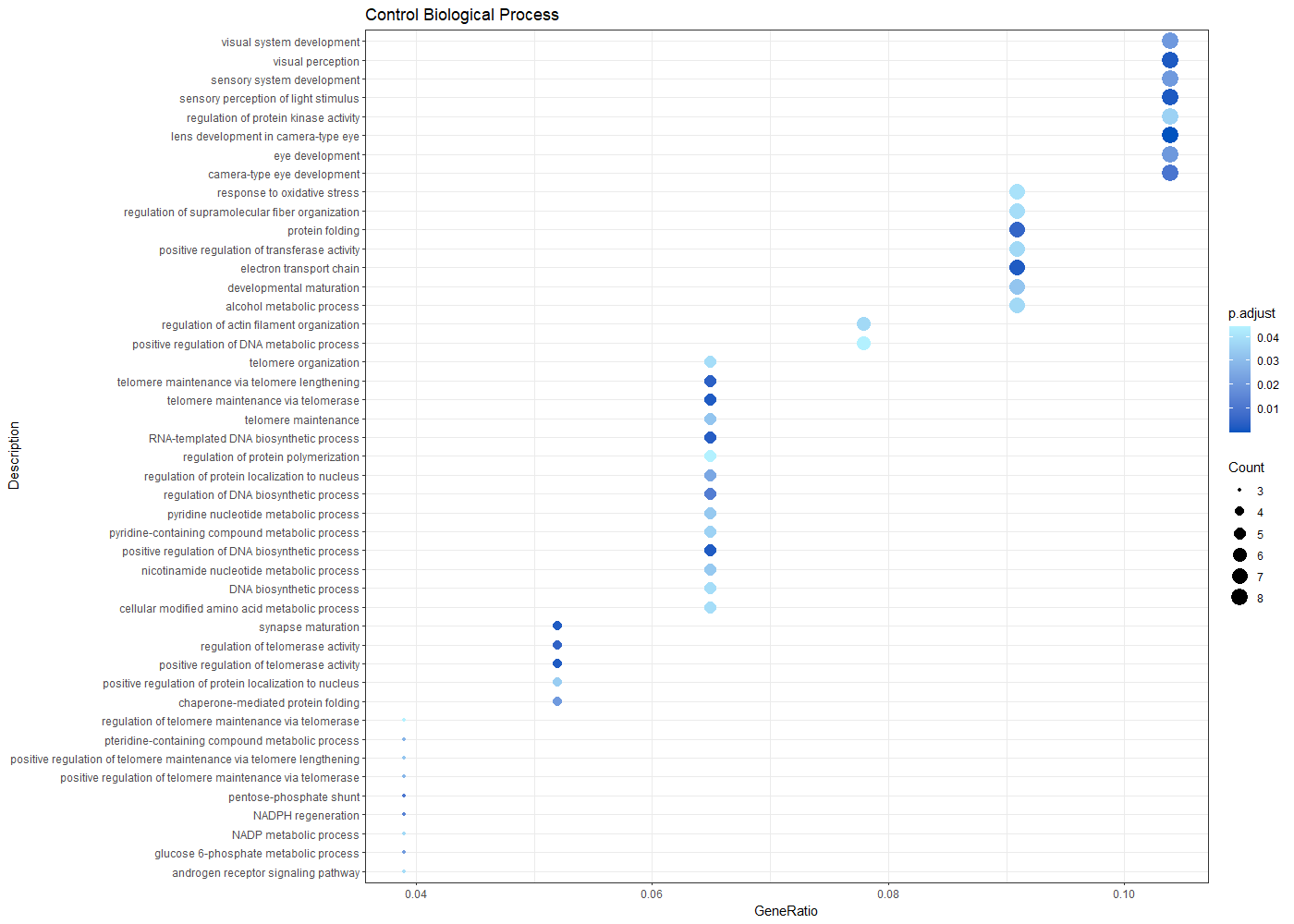


**Supplementary Figure 1.** (A) Cellular Component (CC); (B) Biological Processes (BP) charts found enriched in proteins upregulated or exclusive of Ctrl VH. GeneRatio was calculated and data filtered for p≤0.05 after BH correction. The table shows terms with count ≥3 for BP.

A


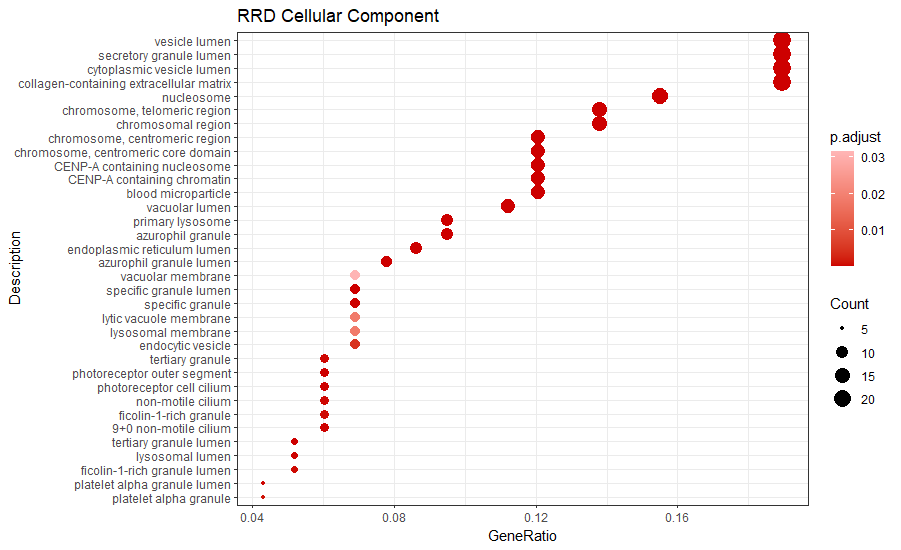


B


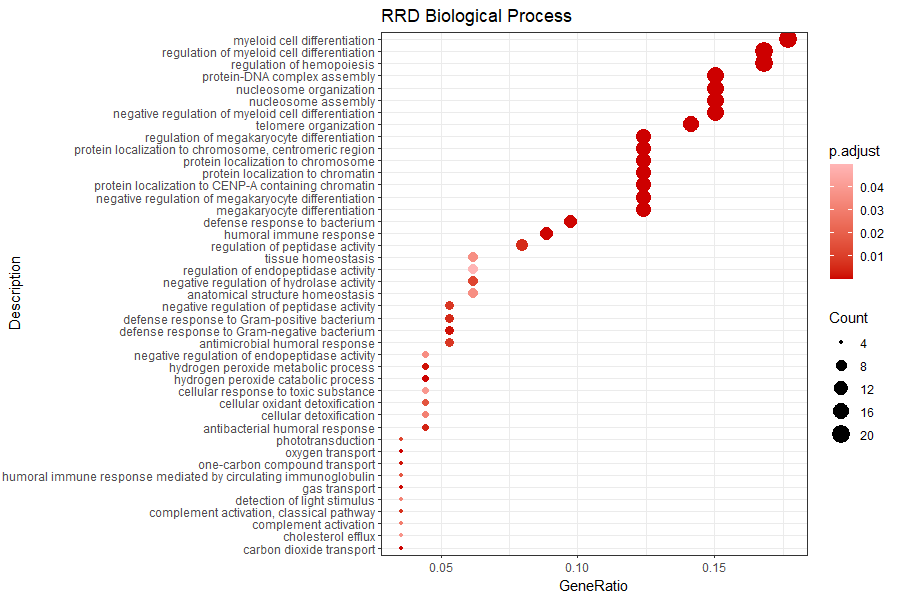


**Supplementary Figure 2.** (A) Cellular Component (CC); (B) Biological Processes (BP) charts found enriched in proteins upregulated or exclusive of RRD VH. GeneRatio was calculated and data filtered for p≤0.05 after BH correction. The table shows terms with count ≥5 and 6, for A and B figures, respectively.





**Supplementary Figure 3.** Volcano plot showing differentially expressed N-termini. X-axis reports the fold change expressed as log2 fold-change (Log2FC); Y-axis reported the -log10 p.mod value. Statistical significance was set for p.mod≤0.05. Dashed lines highlighted the cut-off set for log2FC and log_10_ of p.mod significance, namely: ±0.57 and 1.3, respectively. N-termini upregulated and downregulated in RRD vs Ctrl VH are represented in red and turquoise, respectively.


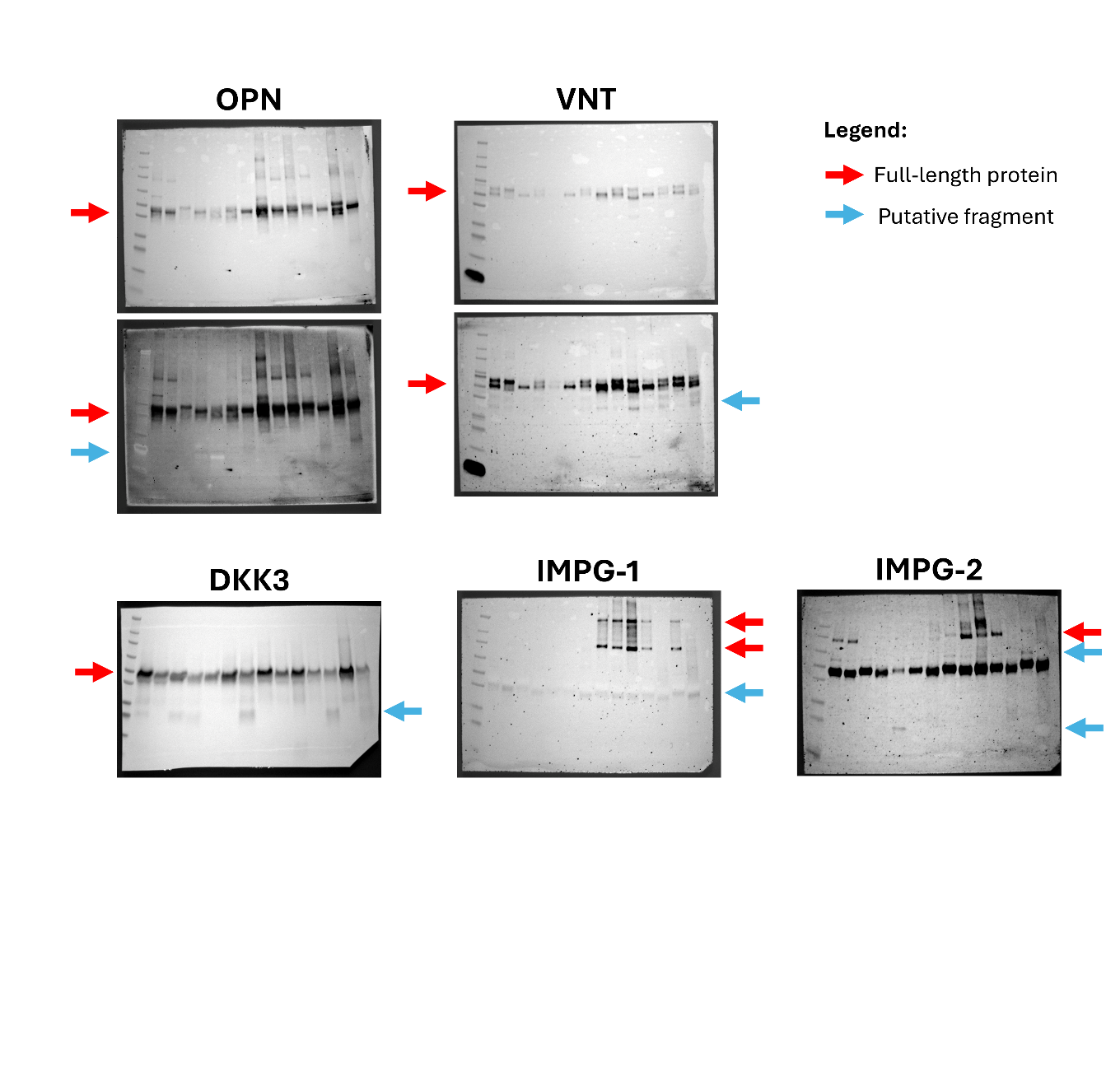


**Supplementary Figure 4.** Uncropped Western blotting figures used in this study. As indicated in the legend, red arrows point to the full-length protein, blue arrows to putative cleavage fragments.
